# Supplementary material for: Intercropping on Mars: A promising system to optimise fresh food production in future martian colonies
Source: PLoS One. 2024 May 1;19(5):e0302149. doi: 10.1371/journal.pone.0302149 (PMC11062560; doi:10.1371/journal.pone.0302149)
Supplement: S1 Appendix — Values are given in weight percentage (wt%). The table contains compiled data taken from multiple sources and own measurements. (DOCX) [file pone.0302149.s001.docx]

# **Appendix 1**

| **Comparison of the mineralogical, physical and chemical properties between the Mars regolith from the Rocknest eolian deposit in the Gale crater on Mars and the MMS-1 Mars regolith simulant.** Values are given in weight percentage (wt%). The table contains compiled data taken from multiple sources and own measurements. | | |
| --- | --- | --- |
| **Concentration**  **(wt%)** | **Mars regolith**  **(Rocknest bulk)** | **Mars regolith simulant**  **(MMS-1)** |
| SiO2 | 43 | 49.4 |
| TiO2 | 1.2 | 1.09 |
| Al2O3 | 9.4 | 17.1 |
| Cr2O3 | 0.5 | 0.05 |
| Fe2O3 | – | 10.87 |
| FeO | 19.2 | – |
| MnO | 0.4 | 0.17 |
| MgO | 8.7 | 6.08 |
| CaO | 7.3 | 10.45 |
| Na2O | 2.7 | 3.28 |
| K2O | 0.5 | 0.48 |
| P2O5 | 1 | 0.17 |
| SO3 | 5.5 | 0.1 |
| Cl | 0.7 | – |
| Total composition % | 100 | 100 |
| pH | 7.7 ± 0.5^1^ | 7.28 +/− 0.1^2^ |
| Particle Size | <2mm | <1mm |
| Source | [1] | [2] |
| ^1^The pH value is absent from Gale crater data. The value of 7.7± 0.5 is from data collected at the Mars Phoenix Lander site [3]. ^2^This pH value was measured directly from the regolith that we used, before the start of the experiment. | | |

# **References**

1. Achilles CN, Downs RT, Ming DW, Rampe EB, Morris R V., Treiman AH, et al. Mineralogy of an active eolian sediment from the Namib dune, Gale crater, Mars. J Geophys Res Planets. 2017;122: 2344–2361. doi:10.1002/2017JE005262

2. Peters GH, Abbey W, Bearman GH, Mungas GS, Smith JA, Anderson RC, et al. Mojave Mars simulant-Characterization of a new geologic Mars analog. Icarus. 2008;197: 470–479. doi:10.1016/j.icarus.2008.05.004

3. Hecht MH, Kounaves SP, Quinn RC, West SJ, Young SMM, Ming DW, et al. Detection of Perchlorate and the Soluble Chemistry of Martian Soil at the Phoenix Lander Site. Science (1979). 2009;325.
