# Supplementary material for: Intercropping on Mars: A promising system to optimise fresh food production in future martian colonies
Source: PLoS One. 2024 May 1;19(5):e0302149. doi: 10.1371/journal.pone.0302149 (PMC11062560; doi:10.1371/journal.pone.0302149)
Supplement: S2 Appendix — (DOCX) [file pone.0302149.s002.docx]

# **Appendix 2**

| **pH, EC and nutrient contents of Hoagland nutrient solution.** | | | | | | | | | | | | | | | | | | |
| --- | --- | --- | --- | --- | --- | --- | --- | --- | --- | --- | --- | --- | --- | --- | --- | --- | --- | --- |
| **pH** | **EC** | **NH_4_** | **K** | **Na** | **Ca** | **Mg** | **NO3** | **Cl** | **S** | **HCO_3_** | **P** | **Si** | **Fe** | **Mn** | **Zn** | **B** | **Cu** | **Mo** |
|  | (mS/cm) | (mmol/l) | | | | | | | | | | | (µmol/l) | | | | | |
| 6,0 | 1,9 | 0,8 | 6,4 | 0,6 | 3,6 | 1,2 | 10,2 | 0,4 | 1,8 | 0,9 | 2,07 | 0,15 | 51 | 13 | 5,2 | 28 | 1,0 | 0,5 |
