# Supplementary material for: Intercropping on Mars: A promising system to optimise fresh food production in future martian colonies
Source: PLoS One. 2024 May 1;19(5):e0302149. doi: 10.1371/journal.pone.0302149 (PMC11062560; doi:10.1371/journal.pone.0302149)
Supplement: Attachment — Submitted filename: Peer review for Manuscript PONE.pdf [file pone.0302149.s003.pdf]

Peer review for Manuscript PONE-D-23-12961

Overall, the authors present a novel study with an interesting premise, good study design, and overall suitable metrics to measure their results. The growth of plants in-situ in regolith is a complex and relatively new topic and with limited studies on this subject, these studies will lay groundwork for later experiments. The use of agricultural metrics with intercropping in this paper bring a fresh approach to regolith studies and the use of a plant-rhizobium symbiosis builds on existing literature. The question and build up is mostly there to give context for intercropping in the introduction and the sources cited are relevant articles. However, one concern I have with this sort of research is that it does not address some of the crucial issues that growing things in-situ on Mars would encounter. That does not make this any less useful, but it maybe good to mention some of the other things that would need to be dealt with just to highlight where the focus of this research is. The data provided adequately supports the authors claims, but the discussion could use some elaboration to better understand how it fits the context of the field as a whole. The paper is an important contribution to a growing field and with a few minor revisions I would recommend this paper for publication. I have below outlined proposed revisions and additions to the article.

Major revisions:

None

Minor revisions:

Introduction

Generally speaking, it may be good to address some of the current literature on problems with simulant experiments that have been brought up. Issues like presence of perchlorates and other obstacles that maybe an issue should be briefly mentioned. On this note, addressing the soil properties of regolith a bit more in depth would help readers to understand why it is important to reestablish agricultural knowledge in this medium.

Line	Revisions recommended
160-163	A justification for why Rhizobium Leguminosarum (RL) was used maybe helpful. This study does not address the specifics of the symbiosis nor study it in detail. There is nothing wrong with this as the focus of the study was elsewhere, but I would alter the language and explain the choice of using RL as a symbiote over something like Sinorhizobium meliloti which has been tested in multiple studies. Also, the species “meliloti” is spelled incorrectly in the manuscript.

Methods

Line	Revisions recommended
257	Hoagland solution misspelled as Hoogland solution

Discussion

One thing to acknowledge in the discussion is the use of Hoagland solution and addition of soil to the regolith and sand. Native Martian regolith (and most simulants) have no nitrogen in them. Adding fertilizer like this inherently alters the fertility of the regolith and thus the focal study system. This maybe needed to support the targets of your study, but it needs to be acknowledged.

Line	Revisions recommended
524-543	Finding some more sources about bacteria or bacterial symbiosis in regolith to help support this section would be excellent. It has a good start and I would say it is almost there but I think there are a few key aspects missing such as pH and presence of toxins in regolith.

Conclusions

Line	Revisions recommended
596-598	I think this line needs some qualifying elements to it, as it is it overextends a bit. Under what conditions did this experiment occur? This is not regolith as such, there are issues to overcome. Something along the lines of "with the addition of X nutrients".

Appendix

Line	Revisions recommended
883	Table unreadable in PDF format
