# Supplementary material for: Intercropping on Mars: A promising system to optimise fresh food production in future martian colonies
Source: PLoS One. 2024 May 1;19(5):e0302149. doi: 10.1371/journal.pone.0302149 (PMC11062560; doi:10.1371/journal.pone.0302149)
Supplement: Attachment — Submitted filename: PONE-D-23-12961_R1_reviewer.pdf [file pone.0302149.s005.pdf]

PLOS ONE

Intercropping on Mars: A Promising System to Optimise Fresh Food Production in Future Martian Colonies

--Manuscript Draft--

**Manuscript Number:**	PONE-D-23-12961R1
**Article Type:**	Research Article
**Full Title:**	Intercropping on Mars: A Promising System to Optimise Fresh Food Production in Future Martian Colonies
**Short Title:**	Intercropping on Mars
**Corresponding Author:**	Rebeca Gonçalves, MSc<br>Wageningen University & Research<br>Wageningen, NETHERLANDS
**Keywords:**	mixed cropping; resource use efficiency; resource optimization; astrobiology; exobiology
**Abstract:**	Future colonists on Mars will need to produce fresh food locallyto acquire key nutrients lost in food dehydration, the primary technique for sending food to space. Martian colonies will operate under limited space, energy, and Earth supplies. We aimed to test the viability and prospect of applying an intercropping system as a method for soil-based food production in Martian colonies, to optimise resource use and enhance colony self-sustainability. A likely early Martian agricultural setting was simulated using small pots, a controlled greenhouse environment, and species compliant with space mission requirements. Pea (Pisum sativum), carrot (Daucus carota) and tomato (Solanum lycopersicum) were grown in three types of soil (“MMS-1” Mars regolith simulant, potting soil and sand), planted either mixed (intercropping) or separate (monocropping). Rhizobia bacteria (Rhizobium leguminosarum) was added as the pea symbiont for Nitrogen-fixing. Plant performance was measured as above-ground biomass, yield, harvest index, and Nitrogen, Phosphorus and Potassium content in yield. The overall intercropping system performance was calculated as total relative yield (RYT). Intercropping had clear effects on plant performance in Mars regolith, being beneficial for tomato but mostly detrimental for pea and carrot, ultimately giving an overall yield disadvantage compared to monocropping (RYT = 0.93). This effect likely resulted from the observed absence of Rhizobia nodulation in Mars regolith, negating Nitrogen-fixation and preventing intercropped plants from fully leveraging their complementary properties. Adverse regolith conditions—high pH, elevated compactness and nutrient deficiencies—presumably restricted Rhizobia survival/nodulation. In sand, where more favourable soil conditions promoted extremely effective nodulation, intercropping significantly outperformed monocropping (RYT = 1.32). Given this, we suggest that with simple improvements to the regolith, enhancing conditions for nodulation, intercropping’s potential could be effectively unlocked, showing promise for the use of this method to optimise food production in Martian colonies. Specific regolith ameliorations are proposed for future research.
**Order of Authors:**	Rebeca Gonçalves, MSc
	G. W. Wieger Wamelink, MSc, PhD
	Peter van der Putten, BSc
	Jochem B. Evers, MSc, PhD
**Response to Reviewers:**	Dear Dr. Harris,<br><br>Thank you for your kind feedback on our submitted manuscript. In our attached rebuttal letter, we have indicated in blue the actions taken to address each of your suggested revisions.<br><br>We hope to have properly addressed your concerns.<br><br>Kind regards,<br>Rebeca Goncalves, on behalf of my Co-Authors

Powered by Editorial Manager® and ProduXion Manager® from Aries Systems Corporation

Additional Information:	
**Question**	**Response**
**Financial Disclosure**<br><br>Enter a financial disclosure statement that describes the sources of funding for the work included in this submission. Review the submission guidelines for detailed requirements. View published research articles from *PLOS ONE* for specific examples.<br><br>This statement is required for submission and **will appear in the published article** if the submission is accepted. Please make sure it is accurate.<br><br>**Unfunded studies**<br>Enter: *The author(s) received no specific funding for this work.*<br><br>**Funded studies**<br>Enter a statement with the following details:<br>• Initials of the authors who received each award<br>• Grant numbers awarded to each author<br>• The full name of each funder<br>• URL of each funder website<br>• Did the sponsors or funders play any role in the study design, data collection and analysis, decision to publish, or preparation of the manuscript?<br>• **NO** - Include this sentence at the end of your statement: *The funders had no role in study design, data collection and analysis, decision to publish, or preparation of the manuscript.*<br>• **YES** - Specify the role(s) played.<br><br>* typeset	The author(s) received no specific funding for this work.
**Competing Interests**<br><br>Use the instructions below to enter a competing interest statement for this submission. On behalf of all authors, disclose any competing interests that could be perceived to bias this work—acknowledging all financial support and any other relevant financial or non-financial competing interests.	The authors have declared that no competing interests exist.

Powered by Editorial Manager® and ProduXion Manager® from Aries Systems Corporation

This statement is required for submission and **will appear in the published article** if the submission is accepted. Please make sure it is accurate and that any funding sources listed in your Funding Information later in the submission form are also declared in your Financial Disclosure statement.<br><br>View published research articles from *PLOS ONE* for specific examples.<br><br>**NO authors have competing interests**<br><br>Enter: *The authors have declared that no competing interests exist.*<br><br>**Authors with competing interests**<br><br>Enter competing interest details beginning with this statement:<br><br>*I have read the journal's policy and the authors of this manuscript have the following competing interests: [insert competing interests here]*<br><br>* typeset	
**Ethics Statement**<br><br>Enter an ethics statement for this submission. This statement is required if the study involved:<br><br>• Human participants<br>• Human specimens or tissue<br>• Vertebrate animals or cephalopods<br>• Vertebrate embryos or tissues<br>• Field research<br><br>Write "N/A" if the submission does not require an ethics statement.<br><br>General guidance is provided below. Consult the submission guidelines for detailed instructions. **Make sure that all information entered here is included in the**	N/A

**Methods section of the manuscript.**<br><br>**Format for specific study types**<br><br>**Human Subject Research (involving human participants and/or tissue)**<br>• Give the name of the institutional review board or ethics committee that approved the study<br>• Include the approval number and/or a statement indicating approval of this research<br>• Indicate the form of consent obtained (written/oral) or the reason that consent was not obtained (e.g. the data were analyzed anonymously)<br><br>**Animal Research (involving vertebrate animals, embryos or tissues)**<br>• Provide the name of the Institutional Animal Care and Use Committee (IACUC) or other relevant ethics board that reviewed the study protocol, and indicate whether they approved this research or granted a formal waiver of ethical approval<br>• Include an approval number if one was obtained<br>• If the study involved *non-human primates*, add *additional details* about animal welfare and steps taken to ameliorate suffering<br>• If anesthesia, euthanasia, or any kind of animal sacrifice is part of the study, include briefly which substances and/or methods were applied<br><br>**Field Research**<br><br>Include the following details if this study involves the collection of plant, animal, or other materials from a natural setting:<br>• Field permit number<br>• Name of the institution or relevant body that granted permission	
**Data Availability**<br><br>Authors are required to make all data underlying the findings described fully available, without restriction, and from the time of publication. PLOS allows rare exceptions to address legal and ethical concerns. See the PLOS Data Policy and FAQ for detailed information.	Yes - all data are fully available without restriction

A Data Availability Statement describing where the data can be found is required at submission. Your answers to this question constitute the Data Availability Statement and **will be published in the article**, if accepted.<br><br>**Important:** Stating 'data available on request from the author' is not sufficient. If your data are only available upon request, select 'No' for the first question and explain your exceptional situation in the text box.<br><br>Do the authors confirm that all data underlying the findings described in their manuscript are fully available without restriction?	
**Describe where the data may be found in full sentences. If you are copying our sample text, replace any instances of XXX with the appropriate details.**<br><br>• If the data are **held or will be held in a public repository**, include URLs, accession numbers or DOIs. If this information will only be available after acceptance, indicate this by ticking the box below. For example: *All XXX files are available from the XXX database (accession number(s) XXX, XXX.).*<br>• If the data are all contained **within the manuscript and/or Supporting Information files**, enter the following: *All relevant data are within the manuscript and its Supporting Information files.*<br>• If neither of these applies but you are able to provide **details of access elsewhere**, with or without limitations, please do so. For example:<br><br>*Data cannot be shared publicly because of [XXX]. Data are available from the XXX Institutional Data Access / Ethics Committee (contact via XXX) for researchers who meet the criteria for access to confidential data.*<br><br>*The data underlying the results*	All relevant data are within the manuscript.

presented in the study are available from (include the name of the third party and contact information or URL).<br>• This text is appropriate if the data are owned by a third party and authors do not have permission to share the data.<br><br>* typeset	
Additional data availability information:	

Powered by Editorial Manager® and ProduXion Manager® from Aries Systems Corporation

Manuscript (revised clean copy) Click here to access/download;Manuscript;MANUSCRIPT (revised clean copy).docx

Intercropping on Mars: A Promising System to Optimise Fresh Food Production in Future Martian Colonies

Rebeca Gonçalves[1]*, G.W. Wieger Wamelink[2], Peter van der Putten[3], Jochem B. Evers[4].

[1] Centre for Crop System Analysis, Wageningen University & Research, Wageningen, The Netherlands

[2] Wageningen Environmental Research, Wageningen University & Research, Wageningen, The Netherlands. Wieger.wamelink@wur.nl

[3] Centre for Crop System Analysis, Wageningen University & Research, Wageningen, The Netherlands

[4] Centre for Crop System Analysis, Wageningen University & Research, Wageningen, The Netherlands

*Corresponding author

E-mail: rebeca.rgoncalves@gmail.com

Abstract

Future colonists on Mars will need to produce fresh food locally to acquire key nutrients lost in food dehydration, the primary technique for sending food to space. Martian colonies will operate under limited space, energy, and Earth supplies. We aimed to test the viability and prospect of applying an intercropping system as a method for soil-based food production in Martian colonies, to optimise resource use and enhance colony self-sustainability. A likely early Martian agricultural setting was simulated using small pots, a controlled greenhouse environment, and species compliant with space mission requirements. Pea (*Pisum sativum)*, carrot (*Daucus carota*) and tomato (*Solanum lycopersicum*) were grown in three types of soil ("MMS-1" Mars regolith simulant, potting soil and sand), planted either mixed (intercropping) or separate (monocropping). Rhizobia bacteria (*Rhizobium leguminosarum*) was added as the pea symbiont for Nitrogen-fixing. Plant performance was measured as above-ground biomass, yield, harvest index, and Nitrogen, Phosphorus and Potassium content in yield. The overall intercropping system performance was calculated as total relative yield (RYT). Intercropping had clear effects on plant performance in Mars regolith, being beneficial for tomato but mostly detrimental for pea and carrot, ultimately giving an overall yield disadvantage compared to monocropping (RYT = 0.93). This effect likely resulted from the observed absence of Rhizobia nodulation in Mars regolith, negating

Nitrogen-fixation and preventing intercropped plants from fully leveraging their complementary properties. Adverse regolith conditions—high pH, elevated compactness and nutrient deficiencies—presumably restricted Rhizobia survival/nodulation. In sand, where more favourable soil conditions promoted extremely effective nodulation, intercropping significantly outperformed monocropping (RYT = 1.32). Given this, we suggest that with simple improvements to the regolith, enhancing conditions for nodulation, intercropping's potential could be effectively unlocked, showing promise for the use of this method to optimise food production in Martian colonies. Specific regolith ameliorations are proposed for future research.

Key words: Mixed cropping, resource use efficiency, resource optimization, astrobiology, exobiology

Introduction

As we enter a new age of space exploration, having a permanent settlement on Mars is now a reality in the not-so-distant future. Conquering the red planet is an exciting endeavour, but also one that brings several scientific, economic, and strategic benefits to our own planet and species.

Whilst rovers have begun to pave our way on Mars, the next stage of exploration and research would need to involve sending humans to perform the work themselves, as humans are much more autonomous and efficient at accomplishing field tasks and carrying out research (1). However, having humans on Mars means they need to have their basic necessities covered, and this includes food. To supply and resupply from Earth all the food needs of a long-term settlement is both impractical as well as economically inviable, which means that the crew will have to make use of local Martian resources to produce at least part of their food needs locally (2).

The production of fresh food would in fact be an indispensable factor for any Martian colony. A bioregenerative food system, one where all resources are provided and produced *in situ* with minimal or no resupply from Earth, carries many advantages over the pre-packaged dehydrated foods that astronauts usually eat. As well as having the need for less food to be brought aboard the spacecraft (a factor that also significantly reduces the financial cost of the mission), a chief advantage of fresh food over dehydrated food is the retainment of nutrients essential to human health, especially antioxidants such as vitamin C and β-carotene, both of which are partially reduced or completely destroyed in the process of dehydration (3–5). Although supplementary pills could be considered, fresh food provides a much better bioavailability of nutrients in the body, mediated via a complex array of other compounds present (6), and it further decreases the need to rely on resupplies from Earth for essential dietary requirements. The act of growing fresh food itself can also have a great positive impact on the mental well-being of colonists. Research on long-duration space missions, as well as analogue isolation simulations on Earth, have identified psychological and psychosocial factors as two of the most critical problems astronauts will face in their off-world missions (7,8). Practising gardening activities has been repeatedly shown to be a significant and effective reliever of stress, anxiety and depression (9,10), and astronauts themselves have in the past expressed deep satisfaction at tending to plants in space (11).

Prospects and challenges of Martian agriculture

Several methods for implementing bioregenerative food systems on Mars have been studied, most of which were focused on artificial growing media such as hydroponics and aquaponics. However, soil-based systems, which would involve making use of the available *in situ* Martian regolith, have recently come to new light as having fundamental features for the long-term sustainability of a colony on Mars. Such a system confers many practical and economic advantages over artificial growing media, such as offering better buffering capacity for operational errors and technical breaks (12), and harbouring a microbiome environment that could compensate for toxic build-up of trace gases in the growth chamber (11). Moreover, inedible crop parts and food waste, as well as human waste, can be composted and added to the soil to provide nutrients for the next harvest. This adds a circularity factor to the system that would minimize, and eventually eliminate, any need for expensive resupplies of nutrients from Earth (13).

Gardening on Mars *is* viable. Although Mars has a very thin atmosphere (1% that of Earth), it contains the necessary elements for plant growth, such as carbon dioxide (CO_2) and nitrogen (N_2) (14), and the planet is abundant in (frozen) water present both in the polar caps and in several places under the Martian surface (15). Because of its thin atmosphere, Mars has high radiation levels on the surface which can be harmful to both plants and humans (16), and the planet holds an average global temperature of -65°C (17). To counteract these conditions, colonies on Mars will likely consist of closed habitats that can shield both plants and humans from harmful radiation, and that can maintain the optimum temperature and atmospheric conditions for life within the colony (18). Gravity on Mars is about 38% that of Earth (17). However, experiments done on plant growth under microgravity conditions aboard the International Space Station (ISS) showed that plants, including crop species such as lettuce, cabbage and mustard, could be successfully grown, harvested and safely consumed by astronauts (19), showing promise that the lower gravity will not be a major impediment for normal plant development on Mars.

There are some potential obstacles with the Mars regolith that need to be overcome in order to successfully grow plants using *in situ* resources. For example, samples from Mars missions have shown the presence of heavy metals and perchlorates in the Martian regolith across different landing sites (20), harmful to both plants and humans. However, phytoremediation and bioremediation offer potential solutions to this, where specific plants or microbes can be cultivated to remove such elements from the soil with up to 100% efficacy (21–23). Moreover, there’s the suggestion that perchlorates would not be present in deeper layers of Martian regolith, since their formation on Mars is proposed to be largely due

to the regolith interaction with cosmic radiation and other atmospheric processes (24). Additional concerns include the regolith's high pH (~8.5) and nutrient deficiency (especially of nitrogen), both detrimental to plant growth and health. To address these, the addition of compost to the soil could lower the pH (25) while also providing the lacking nutrients. An initial batch of fertilizer could be incorporated in first missions' cargo supply, until a cyclic bioregenerative system could be established. Such system could use inedible parts from the crops grown *in situ* as compost for the next crop generation, or make use of human faeces as manure (26).

Optimizing resource use in Martian colonies

On Earth, it is possible to test the viability of using Martian soil (and thus of having a Martian soil-based system) for crop growth using Mars regolith simulants available. Several crop growth experiments have been successfully carried out on Earth using such simulants, with over 20 different species of crops reported to be able to grow and produce yield (27–29). However, the main focus of these pioneering experiments was to test whether plants *could* germinate, grow and produce yield in the Mars regolith. No attention has yet been given to developing the *efficiency* of the system. Since colonies on Mars will probably operate under a limited amount of energy, space and other resources, any crop growth method that increases the productivity and circularity of the system, in a way that optimizes the use of such space and resources, will be paramount to the success and viability of the mission.

Intercropping is an ancient technique used on Earth that involves growing two or more crop species simultaneously in the same field (30). This differs from the "traditional" monocropping system, where only a single crop species is planted in the same field. Intercropping has long been known to produce several beneficial effects compared to monocropping, such as increasing plant biomass and yield of one or more of the component species, improving the nutritional value of yield, and optimizing the use of resources in the system (31–33). Such effects have been observed both in the field as well as in greenhouse pot experiments (34–36). The beneficial effects of intercropping are often measured as a more efficient use of resources compared to the performance of each separate species in a monocropping system.

One way intercrop productivity benefits can arise is when the interspecific competition (competition between different species) in the intercropping system is *lower* than the intraspecific competition

(competition amongst the same species) (37). Another way is by means of *species complementarity*, where both (or all) intercropped species can mutually benefit each other within the system. Complementarity can work either by 1) *niche differentiation*, where companion species that are adapted to different niches (i.e. a shallow root species together with a deep root species) can make a more complete use of all available resources in the system, resulting in less competition for resources; or by 2) *resource facilitation*, where one companion species provides or facilitates the up-take of a limiting resource to the other companion species (38). A classic example of resource facilitation in intercropping is seen through legumes, which can provide nitrogen (N) into the intercropping system thanks to their symbiotic relationship with rhizobia bacteria. The bacteria infect the legume´s roots to form "root nodules", wherein they turn atmospheric nitrogen (N_2) into the usable form of ammonia (NH_3) via a process called N fixation (39). This process helps relieve the competitive pressure on the intercropping system, either via the addition of N in soil (40) or via direct N transfer from the legume to the non-legume (41), and often results in the optimization of resources such as lower N fertilizer requirements (31).

The benefits of intercropping towards optimizing yield and resource use make it a prime candidate method to be applied on a soil-based system on Mars. To date, no experiments have ever been done to investigate the effects of intercropping on crops grown in Mars regolith. In this study, we aim to test the viability and prospect of implementing an intercropping system as a potential food production method in future Mars colonies.

Study objective and research question

Our study objective is to investigate the effects of intercropping on crop performance grown in a Mars regolith simulant (from now on also referred to as just "Mars regolith"). Here we quantify plant performance as above-ground biomass, yield, harvest index, and nutritional content of yield as levels of nitrogen (N), phosphorus (P) and potassium (K). We chose three companion species, namely pea (*Pisum sativum*), carrot (*Daucus carota*) and tomato (*Solanum lycopersicum*), for their complementary and nutritional properties, and for the fact that they have all been previously shown to be able to grow and produce yield in Mars regolith simulant (29). Nodulation by rhizobia bacteria in pea roots will also be a key variable observed in this experiment, to interpret the effects that an N-fixing species would have on the interspecific competition within the intercropping system.

Our research question was whether we would see any beneficial effects of intercropping on the performance of pea, carrot and tomato grown in Mars regolith simulant. If plants perform better when intercropped, by making use of the exact same resource input as when they are monocropped, this could prove the efficacy of intercropping as a method for increasing the efficiency of the system.

Materials and Methods

Greenhouse conditions

We performed a pot experiment in a greenhouse on Earth, in Wageningen (51.9692° N, 5.6654° E). During the experimental period, average temperature in the greenhouse was 20.4 ± 1.7°C, relative humidity was 60.9 ± 15%, and day/night cycle was 16/8h. Ambient air was used and no extra CO_2 was added. Lamps yielding 600 watt (HPS 230 volt) were switched on if sunlight intensity was below 150 watt/m^2, and switched off if sunlight intensity was above 250 watt/m^2.

Mars regolith simulant

The regolith analogue used in this experiment was the MMS-1 Mars Regolith Simulant, of unsorted grade (grain <3.17mm, density 1.25g/cm^3), provided by The Martian Garden Company (Austin, USA). This simulant was manufactured under commission by researchers at the National Aeronautics and Space Administration (NASA)'s Jet Propulsion Laboratory (JPL) (42). MMS-1 was designed to be the closest simulate of the regolith found on the Martian surface, especially when in contact with water. For a comparison of physical and chemical properties of the MMS-1 simulant and actual Martian regolith see Appendix 1.

Earth soils and soil preparation

Two "Earth" soils were included in the experimental design: (1) common potting soil, composed mainly of peat with fertilizer (PGmix 15%N-10%P2O5-20%K2O), and (2) river sand. The potting soil was sieved to remove lumpy matter and improve homogeneity. Sand was specially chosen for it being nutrient poor similar to Mars regolith (see also (28)), to test if they would produce comparable results.

For the Mars regolith and the sand treatments, a small amount of organic soil (derived from the sieved potting soil) was mixed at 10% of the total volume, to improve root aggregation and water retention (43). Table 1 shows the pH and Nutrient analysis from samples of the three soils.

Table 1. Nutrient composition and pH for the three soil treatments (MMS-1 Mars regolith simulant, sand and potting soil), collected at the start of the experiment (day 0). Results shown for Mars regolith simulant and sand include a 10% volume of organic matter mixed with each.

**Nutrient (mg/kg)**	**Mars regolith simulant (MMS-1)**	**Sand**	**Potting soil**
N-NH4	4,34	3,71	343,16
P-PO4	0,1	0,1	41,77
N-NO3	15,94	5,27	180,70
Nmin	20,3	9	523,9
pH	8.5 ± 0.2	7.5 ± 0.2	5.5 ± 0.2

Species selection

The species selected for this experiment were pea (*Pisum sativum* cv. 'Prince Albert', Tuin Plus), carrot (*Daucus carota* cv. 'Nantes 2', Buzzy), and dwarf cherry tomato (*Solanum lycopersicum* cv. 'Tiny Tim', Buzzy). These species represent 3 out of the 27 potential food candidates to be incorporated into a space habitat, as proposed in NASA's Life Support Values and Assumptions Document (44), based on various selection criteria such as potential yield, harvest index, horticultural requirements and macronutrient content needed to supplement a crew's diet.

These species have also been chosen for their particular complementary properties which can be mutually beneficial to each other. As a legume, pea can serve as N-fixers in the system in symbiosis with rhizobia bacteria. Carrot helps aerate the soil, which can improve water and nutrient uptake by companion plants (45). Tomato can provide shade for the temperature sensitive carrot and support for the climbing pea (46), and both carrot and tomato release root exudates such as flavonoids that can promote root nodule formation in pea (47). Furthermore, all three species have compostable crop waste that can be mixed with the soil to provide key nutrients to the subsequent crop, reducing the need for fertilizers and promoting circularity in the system (48).

Finally, these species were chosen for their nutrient composition. All three species, in particular carrot and tomato, are high in antioxidants such as vitamin C and β-carotene (49–51). These are an essential

part of the human diet, but most importantly are amongst the few key nutrients that are mostly or completely lost in dehydrated foods (4).

Rhizobia bacteria addition

To provide the peas with their symbionts for N-fixing, 0,75ml of *Rhizobium leguminosarum* (biovar *viciae* strain 248 OD600 ~ 0.784) was added to every pot, including pots not containing pea plants. Inoculation was done 19 days after sowing. The selection of *R. leguminosarum* was based on its well-documented symbiotic relationship with *P. sativum* (52). This particular strain was chosen due to its prior successful nodulation with *P. sativum* in a previous in-house experiment, where the same Mars regolith simulant (MMS-1) was used, thus aiming to control the potential variable of the bacteria not adapting to Martian regolith conditions.

Experimental design

We used a randomised complete block design with the three experimental species (pea, carrot and tomato) grown in three types of soil (MMS-1 Mars regolith simulant, sand and potting soil), divided into two cropping systems (intercropping and monocropping). This gave 12 different treatments with five replicas for each treatment, resulting in 60 pots. The experimental design and treatments can be seen in Fig 1.

Fig 1. Experimental design. A: Overview of experimental treatments. B: Randomised complete block design with five replicas, and intercropping treatment pots randomly rotated. C: Photo of the experiment (day 48).

For the monocropping treatments, three plants of the same species were grown in each pot, whilst for the intercropping treatments, one plant of each species was grown in each pot. 5L Pots were used, 16cm deep and 20cm wide at the top. Pots were filled to a 13cm depth, with 4.55Kg Mars regolith, 5Kg sand and 1.3Kg potting soil. A mesh was placed on the bottom of each pot to keep the soil in place. Stakes were placed to provide climbing support for the peas, with three stakes placed in every pot.

Seeds were sown on 1st December 2021 and the experiment ran for 105 days, with the harvest taking place on 16th March 2022. Seeds were over-sown to ensure the required germination. Thinning of germinated seeds was carried out 10-14 days later. The configuration of the intercropping sowing was always the same, but pots were then randomly rotated, so that the final configuration was fully random.

Watering and nutrient solution

Water saturation point for each soil was measured before the start of the experiment (Mars regolith = 35.7%, potting soil = 119%, sand = 27%). Watering was done once a day, every day, by pouring water on the saucer beneath the pot. For this, a random sample of three pots from each soil type was weighed and the amount of water to be given was determined to be just below the saturation point for that soil. Since the soils had different water retention properties, the amount of water needed per soil differed, and this was done in order to ensure that every treatment received the same amount of water relative to its saturation point.

100ml of 2EC Hoagland nutrient solution was given once a week for the first 68 days, then increased to twice a week for the remainder of days to compensate for the increased biomass from the growing plants. The solution was poured on the topsoil surface. Solution contents can be found in Appendix 2.

Harvesting and Measurements

At harvest, above-ground biomass and yield were weighed for all three species. Both variables were measured in grams per plant (for monocropping treatments these values were added and divided by the number of plants in the pot, so to get the average value per pot). Above-ground biomass included stem, leaves and branches. Yield fresh weight was done separately for ripe and immature tomato fruits, and for pea pods and pea seeds, where only the seeds were considered for yield analysis. Harvesting of pea yield was also done throughout the experiment, as the first pea seeds started to mature on day 65. The first tomato fruits only started to ripen two weeks before the harvest date, so they were left on the plants to be harvested all at once. For carrot yield, the whole carrot from the crown to the tap root was weighed. All pea roots were washed and visually assessed for the presence of rhizobia nodules, which were also dissected and checked for nodule activity using a light microscope (active nodules show an inner pink coloration (53))

All fresh weighed samples were put in ovens to dry at 70°C for 48 hours.

Yield of the three species was analysed for NPK content. Three replicas of each treatment were grinded and analysed using digestion $H_2SO_4/H_2O_2/Se$ + NP on SFA and $H_2SO_4/H_2O_2/Se$ + K on ICP-OES. For tomato, only ripe (red) tomato fruits were analysed.

Calculations

Harvest index (HI) is a measure of the reproductive efficiency, indicating how much of a plant's above-ground biomass is allocated to yield. It was calculated from dry weight data, for all species, according to equation 1:

$$HI\ (\%) = \frac{Y}{Y + ABG} * 100 \qquad (1)$$

Where Y is the yield (g), and ABG is the above ground biomass (g).

The division of the intercropping yield over the monocropping yield of the same species gives the relative yield (RY) for that species (equation 2). A RY of > 0.33 (0.33 being the ratio of each species present in the intercropping treatment) indicates that the species over-yielded in the intercropping treatment compared to its monocropping treatment.

$$RY = \frac{Y_i}{Y_m} \qquad (2)$$

Where Y_i is the yield from the intercropping treatment (per species) (g), and Y_m is the yield from the monocropping treatment (averaged between the number of plants in the pot) (g).

Relative yield total (RYT) is an index to assess whether there was any yield advantage in the intercropping system compared to monocropping. It is a useful measure to assess how efficiently the intercropping system performed as a whole (54). It was calculated according to equation 3:

$$RYT = RYt + RYp + RYc \qquad (3)$$

Where RYt, RYp and RYc are the relative yields of tomato, pea and carrot (respectively). If RYT > 1, this indicates relative yield (and hence resource use) advantage from intercropping, and RYT < 1 indicates relative yield disadvantage from intercropping.

Data analysis

All data was checked for normality and homogeneity using a W test for normality and a Barlett's test for homogeneity. When needed, LOG_{10} transformations were done to meet the two assumptions, and tests were then run on the transformed data. There were only three parameters that did not fit homogeneity, tomato yield P content (P=0.024), and harvest index for peas and for carrots (P=0.011), but those were still included in the analysis.

A two-way ANOVA was performed on the following variables: above-ground biomass dry weight, yield dry weight, harvest index, N content in yield (NY), P content in yield (PY) and K content in yield (KY). For tomato yield, the data also included the dry weight of the immature fruits harvested, since they would become ripe and consumable if harvested later. The ANOVA was run separately for each species, with cropping type and soil type at the interaction level, and block effects accounted for. A post-hoc Fisher's protected LSD test (P<0.05) was performed for significant effects of soil type and the interaction between soil type and cropping type.

An analysis of deviance was performed on the ratios between intercropping and monocropping for each species in every soil (calculated from LOG-transformed data), and the p-values were obtained for the null hypothesis that the ratio equals 1 (i.e. no difference between intercropping and monocropping treatments).

There was a missing value in one of the replicas sent for the NPK analysis of pea yield from the intercropping Mars regolith treatment due to insufficient pea material available. This means that for this treatment, only 2 replicas were analysed for NPK. There were no other missing or deleted values in the data set.

Results

Mars regolith

Tomato showed increased performance in the intercropping treatment compared to the monocropping treatment, with significantly higher above-ground (AG) biomass (P=0.037), yield (P=0.043), and Potassium content in yield (KY) (P<0.001, Table 2, Fig 2 and Fig 3).

Contrary to tomato, carrot showed decreased performance in the intercropping treatment compared to the monocropping treatment, with significantly lower AG biomass (P=0.023), yield (P=0.022), NY (P<0.001) and PY (P=0.011, Table 2).

Pea showed no significant difference in performance between the intercropping and monocropping treatments for any of the variables. However, like carrot, the observed trend showed lower values in the intercropping treatments compared to the monocropping treatments for all variables, with the p-value for N content close to the threshold (P=0.060, Table 2).

There was no significant difference in harvest index between intercropping and monocropping for any of the three species.

Table 2. Average values (N=5) of all six measured variables for tomato, pea and carrot for the intercropping and monocropping treatments, for each of the three soils (MMS-1 Mars regolith simulant, sand and potting soil).

	**Tomato**		**Pea**		**Carrot**	
	**Intercropping**	**Monocropping**	**Intercropping**	**Monocropping**	**Intercropping**	**Monocropping**
**AG[1] Biomass (g)[2]**						
Mars regolith	4.42 (±0.23)** a	2.15 (±0.35) a	0.45 (±0.10) a	1.01 (±0.14) a	0.19 (±0.08)** a	0.60 (±0.13) ab
Sand	6.76 (±3.92) * b	4.45 (±2.00) b	7.41 (±1.59)** c	4.01 (±0.74) b	1.07 (±0.06)** b	1.71 (±0.30) c
Potting soil	24.71 (±1.26)** c	15.68 (±0.28) c	7.72 (±1.11) c	8.18 (±0.88) c	0.79 (±0.26)*** b	4.62 (±0.15) d
**Yield (g)[2]**						
Mars regolith	2.47 (±0.41)** ab	1.29 (±0.37) a	0.25 (±0.14) a	0.46 (±0.08) a	0.36 (±0.15)** a	1.12 (±0.19) a
Sand	5.32 (±0.56)** c	3.26 (±0.45) b	8.82 (±1.89)** c	4.71 (±0.94) b	2.89 (±0.38)*** b	6.46 (±0.37) c
Potting soil	17.18 (±0.76)*** e	8.68 (±0.40) d	8.72 (±0.61) c	10.44 (±1.30) c	2.26 (±0.53)*** b	12.04 (±0.43) d
**Harvest index (%)**						

Mars regolith	34.84 (±3.44) a	34.98 (±3.82) a	22.34 (±10.20) b	30.93 (±4.79) b	63.92 (±8.17) a	65.75 (±2.74) a
Sand	45.85 (±5.31) a	41.73 (±3.24) a	55.24 (±1.20) a	50.17 (±1.65) a	73.80 (±2.73) b	78.98 (±1.90) b
Potting soil	42.80 (±3.41) a	36.66 (±3.22) a	52.74 (±2.86) a	56.07 (±8.29) a	73.04 (±1.62) ab	72.25 (±1.91) ab
**N content in yield (g/kg)**						
Mars regolith	21.93 (±1.16) b	18.47 (±3.51) b	28.35 (±5.10)* a	39.63 (±2.26) a	9.73 (±0.88)*** c	16.03 (±1.64) d
Sand	12.57 (±0.39) a	10.80 (±0.32) a	37.60 (±1.63) a	35.97 (±3.08) a	7.50 (±0.95) abc	6.80 (±0.62) ab
Potting soil	10.87 (±1.01) a	10.13 (±0.34) a	35.20 (±2.43) a	33.80 (±2.33) a	5.13 (±0.74)** a	8.27 (±0.24) bc
**P content in yield (g/kg)**						
Mars regolith	3.96 (±0.37)* ab	3.41 (±0.25) a	4.15 (±0.96) b	5.01 (±0.24) b	1.58 (±0.11)** a	2.74 (±0.22) a
Sand	3.95 (±0.11)* ab	4.58 (±0.05) b	2.90 (±0.11) a	3.25 (±0.54) a	2.45 (±0.32) a	3.01 (±0.14) a
Potting soil	4.40 (±0.04) b	4.36 (±0.11)b	2.90 (±0.20) a	3.75 (±0.62) a	2.30 (±0.52)** a	3.31 (±0.17) a
**K content in yield (g/kg)**						
Mars regolith	55.00 (±3.29)*** c	40.87 (±0.44) b	14.50 (±0.49) b	14.60 (±0.25) b	18.13 (±3.94) a	22.93 (±1.26) a
Sand	35.90 (±1.36)* a	39.70 (±0.38) ab	12.10 (±0.20) a	13.00 (±0.57) a	21.67 (±2.50) a	24.27 (±2.09) a
Potting soil	37.87 (±1.58) ab	35.93 (±0.49) a	12.47 (±0.32) ** a	13.77 (±0.49) a	15.60 (±4.74)* a	24.97 (±2.45) a

1. "AG" = Above-ground

2. Values refer to dry weight

Values in brackets refer to standard error (SE)

Asterisks (*) refer to significant differences between intercropping and monocropping treatments *within* each species from the *same* soil treatment, at the 0.1 level (*), 0.05 level (**) and 0.001 level (***) (Analysis of Deviance).

Letters refer to differences between *soil* treatments within each *variable* for each species. Different letters indicate a significant difference at the 0.05 level (Fisher's LSD test)

Fig 2. Comparison between intercropping and monocropping treatments in Mars regolith simulant. A: intercropping treatment (left) and monocropped tomato (right). B: intercropping treatment (left) and monocropped pea (right). C: intercropping treatment (left) and monocropped carrot (right). Arrows point to the small carrot leaves from the intercropping treatment. Pictures were taken on the day of harvest (day 105).

Comparison between soils

Potting soil produced the same pattern of results as those seen in the Mars regolith simulant, when comparing the intercropping and monocropping treatments (Table 2 and Fig 3). The only exception was KY, where in tomato it showed no significant difference, and in pea it was significantly lower in intercropping compared to monocropping (P=0.029).

Sand also produced a similar pattern of results to Mars regolith, when comparing the intercropping and monocropping treatments (Table 2 and Fig 3), with three exceptions where the values were inverted: Tomato showed no significant difference in KY, but showed an indication of *lower* K in intercropping; Pea showed significantly *higher* AG biomass (P=0.002) and yield (P=0.012) in intercropping compared to monocropping; and carrot showed no difference in NY, but with an indication of *higher* N in intercropping. A last exception was carrot PY, where there was no significant difference between cropping treatments, but the pattern followed being lower for intercropping compared to monocropping.

For all three species, AG biomass, yield, and harvest index mostly followed the pattern of being significantly the highest on potting soil, followed by sand, and lastly by Mars regolith (P<0.001). Where the difference was not significant (P>0.05), the values always followed the same pattern, except in the harvest indexes of tomato intercrop and monocrop, pea intercrop and carrot monocrop, where sand values were slightly higher than in potting soil (Fig 3A-C). The only significant exception to this pattern were carrot AG biomass and yield, where both were lower in the potting soil intercropping treatment compared to the sand monocropping treatment (P<0.001, Fig 4 and 5). A noteworthy change was seen in pea AG biomass and yield, where both went from being significantly lower in sand compared to potting soil in monocropping, to having no significant difference between the two soils in intercropping (AG biomass: P=0.030; yield: P=0.015) (Fig 3A-B).

For NPK content in yield, the pattern seen above was inverted, where Mars regolith was mostly found to give the highest yield NPK values, and potting soil the lowest values, for both cropping treatments. Mars regolith had significantly higher values than both sand and potting soil for tomato NY and KY (P<0.001), pea P (P=0.007) and K (P<0.001), and carrot N (P<0.011). No significant difference was found amongst any other treatments. The only exception to this pattern was tomato PY, where it was significantly higher in Potting soil compared to Mars regolith monocrop, and higher in sand monocrop compared to Mars regolith monocrop (P=0.041, Fig 3D-F).

Fig 3. Comparison between intercropping and monocropping for tomato, pea and carrot in all three soils (Mars regolith simulant, potting soil and sand). A: Above-ground biomass. B: Yield. C:

Harvest index. D: N content in yield (NY). E: P content in yield (PY). F: K content in yield (KY). "Inter" = Intercropping treatment, "Mono" = Monocropping treatment. Letters indicate significant differences between soils within the same cropping treatment and within the same species, at the 0.05 level (Fisher's LSD test). Asterisks (*) indicate significant differences between intercropping and monocropping treatments within the same soil and within the same species, at the 0.05 level (Analysis of Deviance). Error bars represent standard error (SE).

Fig 4: Comparison between intercropping treatment from the three soils. A: Sand. B: Mars regolith simulant. C: Potting soil.

Fig 5. Yield comparison between cropping treatments and between soils. Labels in pictures indicating "Organic soil" refer to the potting soil treatment. A: Intercropped tomato. B: Monocropped tomato. C: Intercropped peas. D: Monocropped peas. E: Intercropped carrots. F: Intercropped carrots.

Sand was the only soil to present an overall yield advantage in the intercropping system compared to monocropping (RYT = 1,32, Table 3 and Fig 6), where both tomato and pea showed yield advantage in intercropping over monocropping (tomato relative yield (RY) = 0.54; pea RY = 0,62), and carrot showed yield disadvantage (RY = 0,15). Potting soil showed no overall yield advantage (RYT = 1,00), where tomato over-yielded (RY = 0,66) but both pea and carrot under-yielded (pea RY = 0,28; carrot RY = 0,06). Mars regolith showed under-yielding in the intercropping system compared to monocropping (RYT = 0,93), where tomato over-yielded (RY = 0,64) but both pea and carrot under-yielded (pea RY = 0,18; carrot RY = 0,11).

Table 3. Relative yields for tomato, pea and carrot in each soil treatment, and the total relative yield (RYT) for the whole intercropping system when compared to monocropping, for each of the three soils (MMS-1 Mars regolith simulant, sand and potting soil).

**Soil**	**Relative yield**	**RYT**

	Tomato	Pea	Carrot	
**Mars regolith**	0,64	0,18	0,11	0,93
**Sand**	0,54	0,62	0,15	1,32
**Potting soil**	0,66	0,28	0,06	1,00

Fig 6. Comparison of intercropping to monocropping ratios and overall system performance for the three species in each of the three soils (Mars regolith simulant, sand and potting soil). Ratios above 1 indicate the variable had a higher value in intercropping relative to monocropping, ratios below 1 indicate the variable had a lower value in intercropping relative to monocropping. RYT (total relative yield) refers to overall yield advantage or disadvantage in the intercropping system. If RYT > 1, this indicates yield advantage from intercropping over monocropping, RYT < 1 indicates yield disadvantage from intercropping over monocropping, and RYT = 1 indicates no difference in yield between the two cropping systems. Variables indicated are above-ground biomass dry weight (g), yield dry weight (g), harvest index (%), and N, P and K content in yield (g/kg)) for each of the three species (tomato, pea and carrot). Asterisks (*) indicate significant differences between intercropping and monocropping values within the same variable, at the 0.05 level.

Presence of Rhizobia bacteria

Visual analysis of roots of peas in both monocropping and intercropping treatments revealed presence of Rhizobia infected nodules in sand and potting soil, but not in Mars regolith (except for one replica from a monocropping treatment which had 3 nodules). Between 17 and 150 nodules were found across the other treatments. There was no significant difference in the number of nodules between potting soil and sand (Fisher's post hoc test P<0.05), although there was on average more nodules found in sand than in potting soil (Fig 7). Both sand and potting soil showed a significantly higher number of root nodules than Mars regolith (P<0.001). There was also on average more nodules in intercropping compared to monocropping for both soils, although there was considerable variation between replicas, and thus this difference was not significant (P=0.100). Dissection of several nodules per replica showed a pink coloration inside, indicating that the nodules were active (Fig 8).

Fig 7. Average number of root nodules on pea roots (N=5) per plant. Values are indicated for the monocropping and intercropping treatments from each of the three soils (Mars regolith, potting soil and sand). Data labels indicate the average value. Letters indicate significant differences between cropping treatment and between soils, at the 0.05 level (Fisher's LSD test). Error bars represent standard error (SE)

Fig 8. Root and nodule comparison between soils. A: Pea roots from Potting soil monocropping treatment, with intact nodules. B: Pea roots from sand monocropping treatment, with nodules dissected, where a pink coloration can be seen, indicating the nodules were active in N-fixing. C: Pea roots from Mars regolith simulant (intercropping treatment), showing thicker and more tortuous roots. D: Pea roots from Mars regolith (monocropping treatment), from the only replica that showed the presence of nodules. These roots also appear thicker and more tortuous.

Discussion

Intercropping effects on RYT for Mars regolith

In Mars regolith, tomato was the only species in the intercropping treatment to show significantly higher performance in both above-ground biomass (AGB) and yield, compared to monocropping. Carrot showed significantly lower AGB and yield in intercropping compared to monocropping, and pea showed no significant difference between the two cropping treatments.

Overall, the intercropping system in Mars regolith showed yield disadvantage over monocropping, with an RYT (total relative yield) value of 0.93. Intercropping advantages or disadvantages can indicate the amount of interspecific competition or facilitation that is occurring within an intercropping system (54). An RYT of less than 1 means that interspecific competition in intercropping was higher than intraspecific competition in monocropping. This is very likely due to the fact that pea did not form root nodules in symbiosis with the Rhizobia in Mars regolith, thus negating its role as N-fixers and the key advantage of having a legume in an intercropping system. Many studies have demonstrated that when intercropped, pea can compensate for increased nutrient competition, as well as for N deficiency in the soil, by releasing root exudates (i.e. flavonoids) that can promote root nodule formation (47,55). This in turn increases fixation of atmospheric N and helps pea both escape the competitive pressure, as well as relieve the pressure for the other species by leaving more N available in the soil. Without the formation of nodules this was not possible, and instead of relieving the competitive pressure, pea became an added competitive load to the intercropping system in Mars regolith.

The contrast in performance between tomato and the other two species can be attributed to the fact that different crops often show different levels of competition strength when intercropped together. Tomato is a dominant species within intercropping systems, and has been shown to have yield advantages when intercropped with legumes (56). In another study by Wu et al. (36), where tomatoes were intercropped with potato onion, intercropping promoted the growth of tomato, whilst it inhibited the growth of potato onion.

Tomato is also generally known to be “heavy feeders” with a high demand for nutrient supply (57), which could mean that they monopolise the resource uptake in the pot. This higher competitive strength of tomato could explain why it had increased yield performance in intercropping compared to

monocropping, since it was put at an advantage compared to pea and carrot, whilst losing this advantage when grown with other tomato plants. It could also explain why in Mars regolith pea and carrot had lower performance in the intercropping treatment, as a higher resource uptake would confer the tomato more nutrients for growth, whilst leaving less resources available to the other two species. Carrot, which depends solely on the nutrients available in the soil, would naturally be at a disadvantage when grown with tomato.

Interestingly, in sand where we saw an adequate number of nodules in pea roots (“adequate” meaning an average of 30 or more nodules per plant (58)), there *was* an overall yield advantage in the intercropping system (RYT = 1.32), where although intercropped carrots underperformed, both pea and tomato showed significantly higher yield compared to monocropping. This higher RYT owes to the fact that pea performed particularly well when intercropped in sand, giving equal or higher values in all variables compared to monocropping.

The success of peas in sand is most likely due to the higher number of root nodules that were formed, particularly in the intercropping treatment, allowing for more atmospheric N-fixing. The higher number of root nodules in the sand treatment compared to potting soil can probably be explained by the lower N content available in sand, a factor that has been shown to promote nodulation (55). Lower N availability has also been linked to an increase in RYT before: in a pea-barley intercrop study, RYT was highest for treatments with no fertilizer compared to treatments with applied fertilizer (59). The high resource demand from the aggressive tomato in the sand intercropping treatment probably caused further decrease in N availability in the soil, which could explain the higher nodulation seen in this treatment (Fig 7).

Potential factors affecting yield performance on Mars regolith

There are a few reasons why nodulation in pea may have been hindered in both Mars treatments. Many studies have shown nodulation and *R. leguminosarum* survival to greatly decrease when under a variety of biotic and abiotic stresses, including high salinity (60) and soil sterility (61), both of which are features of the MMS-1 Mars regolith used in this study. A potentially aggravating condition was that the regolith was very compact in comparison to both potting soil and sand. Although it was of “unsorted grade”, it was composed mostly of very fine particles that gave it a clay-like texture when wet, and it did not drain

well. The roots of the peas in Mars regolith reflected this in that they were smaller, thicker and more tortuous than the roots of peas in sand (and also in potting soil) (Fig 8), which is a common response to denser and coarser soils (62). Since soil texture is known to affect salinity, where clay and moist soils tend to be more saline (63), this could have added a salt stress factor to the Mars regolith, negatively affecting rhizobia bacteria. Moreover, the Mars regolith was low in organic matter. Sterilised soils have been shown to severely hamper formation of nodules, when compared to unsterilised soils containing more varied microbial communities (61). The chemical composition of the Martian regolith has also been suggested to affect nodulation. A study involving legume-Rhizobia symbiosis in Mars regolith simulants showed more nodulation in the MMS-2 regolith simulant compared to MMS-1, where MMS-2 contains added compounds such as Iron Oxide, Magnesium Oxide, Sulfates and Silicates (64). Another similar study on legume-Rhizobia interactions in MMS-1 suggested that the high pH of the regolith may impair Fe uptake by the plants, Fe being a key factor for plants to sustain a healthy relationship with rhizobia, which would in turn negatively impact nodulation (65).

The compactness factor of the regolith could have also posed a problem for plants themselves, regardless of how it may or may not have affected Rhizobia nodulation. Compact soils allow for lower gas diffusion and water conductivity, which can lead to anaerobic conditions in the soil and significantly reduce N availability and nutrient uptake (62), with more compact soils being linked to lower yields (66). The organic matter present in Earth soils greatly contributes to soil quality, both in reducing soil compactness, as well as being rich in nutrients and the micro-organisms that play a part in making nutrients available for plant uptake (67). This could also explain why the absolute values for yield were the highest in potting soil, and why sand also showed higher yield values compared to Mars (although sand is also poor in organic matter, it is not as sterile as the Mars regolith, and may have harboured a wider microbiome that aided in plant performance). The sterile nature of Mars regolith means that, although it has the *chemical elements* necessary to meet the requirements of plant growth (Table 1), these elements could be mostly absent in the bio-available forms that are necessary for their uptake (68), which are often made available by the activity of micro-organisms in the soil.

Nutrient analysis

Potassium content in yield (KY) for Mars regolith intercropped tomato was significantly higher than KY for both sand and potting soil tomato, from both cropping treatments. This could be explained by the fact that optimum K uptake occurs only above 6.5 pH, and is increased further with increased soil moisture

(69), giving Mars regolith an advantageous condition for K uptake especially when compared to potting soil.

PY in Mars monocropped tomato was significantly lower than in Mars intercropped tomato. This could be explained by the fact that P uptake is severely compromised between pH 8 and pH 9, which is exactly where the Mars regolith falls within. However, it is unclear why this was also not the case for carrot and pea PY from Mars regolith, or for the Mars intercropped tomato treatment.

NY for both tomato and carrot were significantly higher in Mars regolith compared to potting soil, even though potting soil had vastly more nitrogen available (table 1). Although higher N availability in soil has been linked to higher yields (70), studies have also shown that this higher availability has either no correlation or a negative correlation (71) with N content in yield. This could explain why in potting soil we see the highest values for yield but also the lowest values for N content in yield, and vice-versa for Mars regolith.

Mars Regolith limitations and proposed soil ameliorations

Physical and chemical properties of the Mars regolith, such as soil compactness and high pH, may have made it a hostile environment for survival and nodulation of rhizobia bacteria, as well as causing reduced nutrient availability and bioavailability in the soil for ideal plant development, impeding the plants from taking full advantage of their complementarity properties in the intercropping system. For instance, when nodulation occurred and peas were optimised for N-fixing, such as in the sand treatment, the intercropping system as it was designed in this experiment gave significant overall yield advantages, indicating the potential for such a method to be used to optimise crop growth on Mars.

Therefore, a chief solution for the improvement of the system would be to work on the sustainable amelioration of the Mars regolith, taking into account a realistic starting scenario on future Martian colonies. For example, initially selecting a higher grain grade of the regolith on Mars, at the onset of the Martian agricultural system, could potentially already aid in reducing soil compactness, improving drainage, salinity conditions, gas diffusion and nutrient availability.

Following the first crop harvest, using inedible parts to mix in as compost to the regolith could also greatly improve soil conditions. The increased organic matter would not only improve conditions for the survival and nodulation of Rhizobia bacteria, but also support the growth of all other species present by improving the regolith's nutrient content, bioavailability and uptake. Adding compost to MMS-1 Mars regolith results in higher plant biomass, with the highest results at 30:70 simulant:compost mixture (43). Other combinations or varying ratios of candidate species can also be tested in order to find the optimum arrangement for the most efficient species complementarity. For example, a study by El-Gaid et al. (72) on intercropping tomato and bean found that the highest RYT values were achieved in a combination of 1 tomato plant : 3 bean plants (RYT = 1.26).

Of course, the absence of nodulation and reduced plant biomass and yield on the Mars regolith can be attributed not only to its physical properties and lack of micro-organisms and nutrients, but also to its intrinsic chemical composition. Further research would need to be conducted to isolate and identify which and how each of the chemical factors of MMS-1 would influence both in the nodulation of rhizobia as well as in the performance of plants.

Finally, actual Mars regolith and the initial conditions of a Martian colony would pose some additional challenges which, due to the emerging nature of this field of study, were purposefully "bypassed" here in order to reduce variables and produce more reliable results with respect to the focal study system. For example, we manually added a minimal amount of nutrients required for plant growth, disregarding the inherent sterility of the *in situ* regolith and presuming that such nutrients could be supplied from Earth or acquired through an established bioregenerative system on Mars. Furthermore, our simulant did not contain any perchlorates, a compound known to be present on the surface of Mars, nor did we analyse the effects of the regolith's heavy metals on plant growth. Although perchlorate and heavy metal presence can be remedied with the addition of plants or microbes to the soil (21,23), further research could include toxicity remediation and study the holistic effects that such measures would have on the microbial community and overall conditions for plant growth in the Martian regolith. As we build upon our collective knowledge for Martian agriculture, future studies can incorporate findings to produce a more overarching understanding of the complete scenario on Mars, including all its challenges and adopted solutions.

Conclusion

In this study we sought to simulate an agricultural scenario likely to be encountered by early Martian colonists, such as the use of small pots, a controlled greenhouse environment, and a nutrient poor Mars regolith simulant with only essential nutrients added. Under such circumstances, we found that an intercropping system can be successful in optimising resource use efficiency if soil conditions are favourable to plant growth and nodulation of N-fixing bacteria, as we saw in the sand treatment (RYT = 1.32). On Mars regolith simulant, intercropping had an overall yield disadvantage compared to monocropping (RYT = 0.93). We postulated that this was most likely due to the absence of rhizobia nodulation in the Mars regolith, which negated the role of pea as a Nitrogen-fixer, impeding crops in the intercropping system from taking full advantage of their complementary properties. We identified that some of the physical and chemical properties of the Mars regolith simulant, such as elevated compactness and high pH, may have created a hostile environment for the survival and nodulation of rhizobia bacteria, while also limiting nutrient availability and bioavailability in the soil necessary for normal crop development. As further research, we suggest considering our proposed soil ameliorations to the Mars regolith, such as utilizing a higher grain grade to reduce soil compactness, and simulate a cyclic system in order to use past harvest waste as compost to increase soil pH and nutrient availability. We acknowledge that certain challenges with the actual regolith and starting conditions on Mars, such as the presence of perchlorates and the initial absolute sterility of the regolith, have been purposefully bypassed in order to maintain the focal objective of the study. Although solutions to such challenges exist, future studies should aim to ultimately integrate all these factors into a single system. As we build towards a more complete and comprehensive knowledge of Martian agriculture, focusing on improving regolith conditions could be key to unlocking the potential of rhizobia-legume interactions and species complementarity, and advance intercropping as a leading method to optimise fresh food production in future Martian colonies.

Acknowledgements

The authors wish to thank Zoë Berkers for help with measurements and harvest, and Paul Goedhart for his advice on the statistics.

References

1. Slakey F, Spudis PD. Robots vs. Humans: Who Should Explore Space? Scientific American sp [Internet]. 2008 Feb [cited 2022 Apr 28];18(1):26–33. Available from: https://www.scientificamerican.com/article/robots-vs-humans-who-should-explore/

2. Graham T, Bamsey M. Editor's note for the topical issue "Agriculture in Space." Open Agric. 2016;1(1):68.

3. Guine RPF, Correia PMR, Correia AC, Goncalves F, Brito MFS, Ribeiro JRP. Effect of Drying Temperature on the Physical-chemical and Sensorial Properties of Eggplant (Solanum melongena L.). Curr Nutr Food Sci. 2017;14(1):28–39.

4. Mohammadi X, Deng Y, Matinfar G, Singh A, Mandal R, Pratap-Singh A. Impact of Three Different Dehydration Methods on Nutritional Values and Sensory Quality of Dried Broccoli, Oranges, and Carrots. Foods [Internet]. 2020 [cited 2022 May 19];9(10):1464. Available from: www.mdpi.com/journal/foods

5. Santos PHS, Silva MA. Retention of Vitamin C in Drying Processes of Fruits and Vegetables—A Review. https://doi-org.ezproxy.library.wur.nl/101080/07373930802458911 [Internet]. 2010 [cited 2022 May 19];26(12):1421–37. Available from: https://www-tandfonline-com.ezproxy.library.wur.nl/doi/abs/10.1080/07373930802458911

6. Pressman P, Clemens RA, Hayes AW. Bioavailability of micronutrients obtained from supplements and food. Toxicology Research and Application. 2017;1:239784731769636.

7. Kanas N, Sandal G, Boyd JE, Gushin VI, Manzey D, North R, et al. Psychology and culture during long-duration space missions. Acta Astronaut [Internet]. 2009 [cited 2022 May 19];64:659–77. Available from: www.elsevier.com/locate/actaastro

8. Morphew ME. Psychological and human factors in long duration spaceflight. McGill Journal of Medicine. 2001;6(1):74–80.

9. Aistis LE; A;, Sachs NA;, Williams M;, Roberts JD, Goldstein R, Gerdes ME, et al. Reducing Anxiety with Nature and Gardening (RANG): Evaluating the Impacts of Gardening and Outdoor Activities on Anxiety among U.S. Adults during the COVID-19 Pandemic. Int J Environ Res Public Health [Internet]. 2022;19:5121. Available from: https://doi.org/10.3390/ijerph19095121

10. Clatworthy J, Hinds J, Camic PM. Gardening as a mental health intervention: A review. Mental Health Review Journal. 2013;18(4):214–25.

11. Nelson M, Dempster WF, Allen JP. Integration of lessons from recent research for "Earth to Mars" life support systems. Advances in Space Research. 2008;41(5):675–83.

12. Maggi F, Pallud C. Space agriculture in micro- and hypo-gravity: A comparative study of soil hydraulics and biogeochemistry in a cropping unit on Earth, Mars, the Moon and the space station. Planet Space Sci. 2010 Dec 1;58(14–15):1996–2007.

13. Silverstone S, Nelson M, Alling A, Allen J. Development and research program for a soil-based bioregenerative agriculture system to feed a four person crew at a Mars base. Advances in Space Research. 2003;31(1):69–75.

14. ESA. Comparing the atmospheres of Mars and Earth [Internet]. 2018 [cited 2022 Aug 20]. Available from: https://www.esa.int/ESA_Multimedia/Images/2018/04/Comparing_the_atmospheres_of_Mars_and_Earth

15. Orosei R, Ding C, Fa W, Giannopoulos A, Hérique A, Kofman W, et al. The Global Search for Liquid Water on Mars from Orbit: Current and Future Perspectives. Life [Internet]. 2020 [cited 2022 Aug 20];10:120. Available from: www.mdpi.com/journal/life

16. Tack N, Wamelink GWW, Denkova AG, Schouwenburg M, Hilhorst H, Wolterbeek HT, et al. Influence of Martian Radiation-like Conditions on the Growth of Secale cereale and Lepidium sativum. Frontiers in Astronomy and Space Sciences. 2021;8(August):1–9.

17. NASA. NASA Science: Mars Exploration Program. 2022 [cited 2022 Aug 20]. Mars facts. Available from: https://mars.nasa.gov/all-about-mars/facts/

18. Liu J, Li H, Sun L, Guo Z, Harvey J, Tang Q, et al. In-situ resources for infrastructure construction on Mars: A review. International Journal of Transportation Science and Technology [Internet]. 2022;11(1):1–16. Available from: https://doi.org/10.1016/j.ijtst.2021.02.001

19. Mariani JF. Veggie Fact Sheet. The Encyclopedia of American Food and Drink. 2020;520–520.

20. Clark BC, Kounaves SP. Evidence for the distribution of perchlorates on Mars. Int J Astrobiol. 2015;15(4):311–8.

21. González Henao S, Ghneim-Herrera T. Heavy Metals in Soils and the Remediation Potential of Bacteria Associated With the Plant Microbiome. Front Environ Sci. 2021;9(April):1–17.

22. Koehle AP, Brumwell SL, Seto EP, Lynch AM, Urbaniak C. Microbial applications for sustainable space exploration beyond low Earth orbit. NPJ Microgravity. 2023;9(1).

23. Misra G, Smith W, Garner M, Loureiro R. Potential Biological Remediation Strategies for Removing Perchlorate from Martian Regolith. New Space. 2021;9(4).

24. Wilson EH, Atreya SK, Kaiser RI, Mahaffy PR. Perchlorate formation on Mars through surface radiolysis-initiated atmospheric chemistry: A potential mechanism. J Geophys Res Planets [Internet]. 2016;121:1472–87. Available from: doi:10.1002/2016JE005078

25. Xu D, Yu X, Chen J, Li X, Chen J, Li JH. Effects of compost as a soil amendment on bacterial community diversity in saline–alkali soil. Front Microbiol. 2023;14(September):1–11.

26. Wamelink GWW, Schug L, Frissel JY, Lubbers I. Growth of Rucola on Mars soil simulant under the influence of pig slurry and earthworms. Open Agric. 2022;7(1):238–48.

27. Ramírez DA, Kreuze J, Amoros W, Valdivia-Silva JE, Ranck J, Garcia S, et al. Extreme salinity as a challenge to grow potatoes under Mars-like soil conditions: targeting promising genotypes. Int J Astrobiol. 2017;18(1):1–7.

28. Wamelink GWW, Frissel JY, Krijnen WHJ, Verwoert MR, Goedhart PW. Can plants grow on mars and the moon: A growth experiment on mars and moon soil simulants. PLoS One. 2014;9(8).

29. Wamelink GWW, Frissel JY, Krijnen WHJ, Verwoert MR. Crop growth and viability of seeds on Mars and Moon soil simulants. Open Agric. 2019;4(1):509–16.

30. Mt.Pleasant J. Food yields and nutrient analyses of the three sisters: A Haudenosaunee cropping system. Ethnobiology Letters. 2016;7(1):87–98.

31. Jensen ES, Carlsson G, Hauggaard-Nielsen H. Intercropping of grain legumes and cereals improves the use of soil N resources and reduces the requirement for synthetic fertilizer N: A global-scale analysis. Agron Sustain Dev. 2020;40(1).

32. Latati M, Bargaz A, Belarbi B, Lazali M, Benlahrech S, Tellah S, et al. The intercropping common bean with maize improves the rhizobial efficiency, resource use and grain yield under low phosphorus availability. European Journal of Agronomy [Internet]. 2016;72:80–90. Available from: http://dx.doi.org/10.1016/j.eja.2015.09.015

33. Li X, Mu Y, Cheng Y, Liu X, Nian H. Effects of intercropping sugarcane and soybean on growth, rhizosphere soil microbes, nitrogen and phosphorus availability. Acta Physiol Plant. 2013;35(4):1113–9.

34. Nasar J, Shao Z, Arshad A, Jones FG, Liu S, Li C, et al. The effect of maize–alfalfa intercropping on the physiological characteristics, nitrogen uptake and yield of maize. Plant Biol. 2020 Nov 1;22(6):1140–9.

35. Patra DD, Subbiah B V. 15N studies on the transfer of legume-fixed nitrogen to associated cereals in intercropping systems. Biol Fertil Soils. 1986;2:165–71.

36. Wu X, Wu F, Zhou X, Fu X, Tao Y, Xu W, et al. Effects of intercropping with potato onion on the growth of tomato and rhizosphere alkaline phosphatase genes diversity. Front Plant Sci. 2016;7(JUNE2016):1–13.

37. Hauggaard-Nielsen H, Ambus P, Jensen ES. Interspecific competition, N use and interference with weeds in pea-barley intercropping. Field Crops Res. 2001;70(2):101–9.

38. Bybee-Finley KA, Ryan MR. Advancing intercropping research and practices in industrialized agricultural landscapes. Agriculture (Switzerland). 2018;8(6).

39. Stern WR. Nitrogen fixation and transfer in intercrop systems. Field Crops Res. 1993;34(3–4):335–56.

40. Gatsios A, Ntatsi G, Celi L, Said-Pullicino D, Tampakaki A, Giannakou I, et al. Nitrogen nutrition optimization in organic greenhouse tomato through the use of legume plants as green manure or intercrops. Agronomy. 2019;9(11).

41. Salgado GC, Ambrosano EJ, Rossi F, Otsuk IP, Ambrosano GMB, Santana CA, et al. Biological n fixation and n transfer in an intercropping system between legumes and organic cherry tomatoes in succession to green corn. Agriculture (Switzerland). 2021;11(8).

42. Peters GH, Abbey W, Bearman GH, Mungas GS, Smith JA, Anderson RC, et al. Mojave Mars simulant-Characterization of a new geologic Mars analog. Icarus. 2008;197(2):470–9.

43. Caporale AG, Vingiani S, Palladino M, El-Nakhel C, Duri LG, Pannico A, et al. Geo-mineralogical characterisation of Mars simulant MMS-1 and appraisal of substrate physico-chemical properties and crop performance obtained with variable green compost amendment rates. Science of the Total Environment [Internet]. 2020;720:137543. Available from: https://doi.org/10.1016/j.scitotenv.2020.137543

44. Anderson MS, Ewert MK, Keener JF, Wagner SA. Life Support Baseline Values and Assumptions Document. Nasa/Tp-2015-218570 [Internet]. 2015;(March):1–220. Available from: http://docs.lib.purdue.edu/cgi/viewcontent.cgi?article=1002&context=nasatr

45. Lipiec J, Medvedev V V., Birkas M, Dumitru E, Lyndina TE, Rousseva S, et al. Effect of soil compaction on root growth and crop yield in Central and Eastern Europe. Int Agrophys. 2003;17(2):61–9.

46. Riotte L. Carrots Love Tomatoes: Secrets of Companion Planting for Successful Gardening. 2nd ed. North Adams, MA: Storey Publishing, LLC; 1998.

47. Leoni F, Hazrati H, Fomsgaard IS, Moonen AC, Kudsk P. Determination of the Effect of Co-cultivation on the Production and Root Exudation of Flavonoids in Four Legume Species Using LC-MS/MS Analysis. J Agric Food Chem. 2021;69(32):9208–19.

48. Ho TTK, Tra VT, Le TH, Nguyen NKQ, Tran CS, Nguyen PT, et al. Compost to improve sustainable soil cultivation and crop productivity. Case Studies in Chemical and Environmental Engineering [Internet]. 2020;1(March):100211. Available from: https://doi.org/10.1016/j.cscee.2022.100211

49. Ali MY, Sina AAI, Khandker SS, Neesa L, Tanvir EM, Kabir A, et al. Nutritional composition and bioactive compounds in tomatoes and their impact on human health and disease: A review. Foods. 2021;10(1).

50. Arscott SA, Tanumihardjo SA. Carrots of many colors provide basic nutrition and bioavailable phytochemicals acting as a functional food. Compr Rev Food Sci Food Saf. 2010;9(2):223–39.

51. Kumari T, Deka SC. Potential health benefits of garden pea seeds and pods: A review. Legume Science. 2021;3(2):1–13.

52. Karunakaran R, Ramachandran VK, Seaman JC, East AK, Mouhsine B, Mauchline TH, et al. Transcriptomic analysis of Rhizobium leguminosarum biovar viciae in symbiosis with host plants Pisum sativum and Vicia cracca. J Bacteriol. 2009;191(12):4002–14.

53. Schumpp O, Deakin WJ. How inefficient rhizobia prolong their existence within nodules. Trends Plant Sci [Internet]. 2010;15(4):189–95. Available from: http://dx.doi.org/10.1016/j.tplants.2010.01.001

54. Fetene M. Intra- and inter-specific competition between seedlings of Acacia etbaica and a perennial grass (Hyparrenia hirta). J Arid Environ. 2003;55(3):441–51.

55. Li Y, Sun J, LI C, Li L, Cheng X, Zhang F. Effects of interspecific interactions and nitrogen fertilization rates on the agronomic and nodulation characteristics of intercropped faba bean. Scientia Agricultura Sinica. 2009;42:3467–3474.

56. Olowolaju ED, Okunlola GO. COMPARATIVE ADVANTAGE OF INTERCROPPING MAIZE, COWPEA AND TOMATO TO SOLE AND MIXED CULTURE. Annals of West University of Timişoara, ser Biology. 2017;20(1):31–8.

57. Traoré A, Bandaogo AA, Savadogo OM, Saba F, Ouédraogo AL, Sako Y, et al. Optimizing Tomato (Solanum lycopersicum L.) Growth With Different Combinations of Organo-Mineral Fertilizers. Front Sustain Food Syst. 2022;5(March):1–7.

58. Farquharson E, Ballard R, Herridge D, Ryder M, Denton M, Webste A, et al. INOCULATING LEGUMES : PRACTICE AND SCIENCE. Grains Research and Development Corporation. 2022;

59. Sahota TS, Malhi SS. Intercropping barley with pea for agronomic and economic considerations in northern Ontario. Agricultural Sciences. 2012;03(07):889–95.

60. Zahran HH. Rhizobium -Legume Symbiosis and Nitrogen Fixation under Severe Conditions and in an Arid Climate . Microbiology and Molecular Biology Reviews. 1999;63(4):968–89.

61. Kasper S, Christoffersen B, Soti P, Racelis A. Abiotic and biotic limitations to nodulation by leguminous cover crops in south Texas. Agriculture (Switzerland). 2019;9(10).

62. Correa J, Postma JA, Watt M, Wojciechowski T. Soil compaction and the architectural plasticity of root systems. J Exp Bot. 2019;70(21):6019–34.

63. Zaman M, Shahid SA, Heng L. Guideline for Salinity Assessment, Mitigation and Adaptation Using Nuclear and Related Techniques. Guideline for Salinity Assessment, Mitigation and Adaptation Using Nuclear and Related Techniques. 2018.

64. Rainwater R, Mukherjee A. The legume-rhizobia symbiosis can be supported on Mars soil simulants. PLoS One [Internet]. 2021;16(12 December):1–10. Available from: http://dx.doi.org/10.1371/journal.pone.0259957

65. Harris F, Dobbs J, Atkins D, Ippolito JA, Stewart JE. Soil fertility interactions with Sinorhizobiumlegume symbiosis in a simulated Martian regolith; effects on nitrogen content and plant health. PLoS One [Internet]. 2021;16(9 September):1–13. Available from: http://dx.doi.org/10.1371/journal.pone.0257053

66. Johansen TJ, Thomsen MG, Løes AK, Riley H. Root development in potato and carrot crops – influences of soil compaction. Acta Agric Scand B Soil Plant Sci [Internet]. 2015;65(2):182–92. Available from: http://dx.doi.org/10.1080/09064710.2014.977942

67. Kong AYY, Scow KM, Córdova-Kreylos AL, Holmes WE, Six J. Microbial community composition and carbon cycling within soil microenvironments of conventional, low-input, and organic cropping systems. Soil Biol Biochem [Internet]. 2011;43(1):20–30. Available from: https://www.ncbi.nlm.nih.gov/pmc/articles/PMC3624763/pdf/nihms412728.pdf

68. Duri LG, Caporale AG, Rouphael Y, Vingiani S, Palladino M, De Pascale S, et al. The Potential for Lunar and Martian Regolith Simulants to Sustain Plant Growth: A Multidisciplinary Overview. Frontiers in Astronomy and Space Sciences. 2022;8(January):1–16.

69. Kuchenbuch R, Claassen N, Jungk A. Potassium availability in relation to soil moisture - I. Effect of soil moisture on potassium diffusion, root growth and potassium uptake of onion plants. Plant Soil. 1986;95(2):221–31.

70. Walley F, Yates T, van Groenigen JW, van Kessel C. Relationships Between Soil Nitrogen Availability Indices, Yield, and Nitrogen Accumulation of Wheat. Soil Science Society of America Journal. 2002;66(5):1549–61.

71. Davis DR, Epp MD, Riordan HD, Davis DR. Changes in USDA Food Composition Data for 43 Garden Crops, 1950 to 1999. J Am Coll Nutr. 2004;23(6):669–82.

72. El-Gaid MAA, Al-Dokeshy MH, Nassef DMT. Effects of intercropping system of tomato and common bean on growth, yield components and land equivalent ratio in new valley governorate. Asian Journal of Crop Science [Internet]. 2014;6(3):254–61. Available from: 10.3923/AJCS.2014.254.261

73. Achilles CN, Downs RT, Ming DW, Rampe EB, Morris R V., Treiman AH, et al. Mineralogy of an active eolian sediment from the Namib dune, Gale crater, Mars. J Geophys Res Planets. 2017;122(11):2344–61.

74. Hecht MH, Kounaves SP, Quinn RC, West SJ, Young SMM, Ming DW, et al. Detection of Perchlorate and the Soluble Chemistry of Martian Soil at the Phoenix Lander Site. Science (1979). 2009;325(July).

Appendix 1

Comparison of the mineralogical, physical and chemical properties between the Mars regolith from the Rocknest eolian deposit in the Gale crater on Mars and the MMS-1 Mars regolith simulant. Values are given in weight percentage (wt%). The table contains compiled data taken from multiple sources and own measurements.

**Concentration (wt%)**	**Mars regolith (Rocknest bulk)**	**Mars regolith simulant (MMS-1)**
SiO_2	43	49.4
TiO_2	1.2	1.09
Al_2O_3	9.4	17.1
Cr_2O_3	0.5	0.05
Fe_2O_3	–	10.87
FeO	19.2	–
MnO	0.4	0.17
MgO	8.7	6.08
CaO	7.3	10.45
Na_2O	2.7	3.28
K_2O	0.5	0.48
P_2O_5	1	0.17
SO_3	5.5	0.1
Cl	0.7	–
Total composition %	100	100
pH	7.7 ± 0.5[1]	7.28 +/− 0.1[2]
Particle Size	<2mm	<1mm
Source	(73)	(42)

[1]The pH value is absent from Gale crater data. The value of 7.7± 0.5 is from data collected at the Mars Phoenix Lander site (74).

[2]This pH value was measured directly from the regolith that we used, before the start of the experiment.

Appendix 2.

pH, EC and nutrient contents of Hoagland nutrient solution.

**pH**	**EC**	**NH_4**	**K**	**Na**	**Ca**	**Mg**	**NO3**	**Cl**	**S**	**HCO_3**	**P**	**Si**	**Fe**	**Mn**	**Zn**	**B**	**Cu**	**Mo**
	(mS/cm)	(mmol/l)											(µmol/l)					
6,0	1,9	0,8	6,4	0,6	3,6	1,2	10,2	0,4	1,8	0,9	2,07	0,15	51	13	5,2	28	1,0	0,5

Figure 1

Click here to access/download;Figure;Fig1.tif

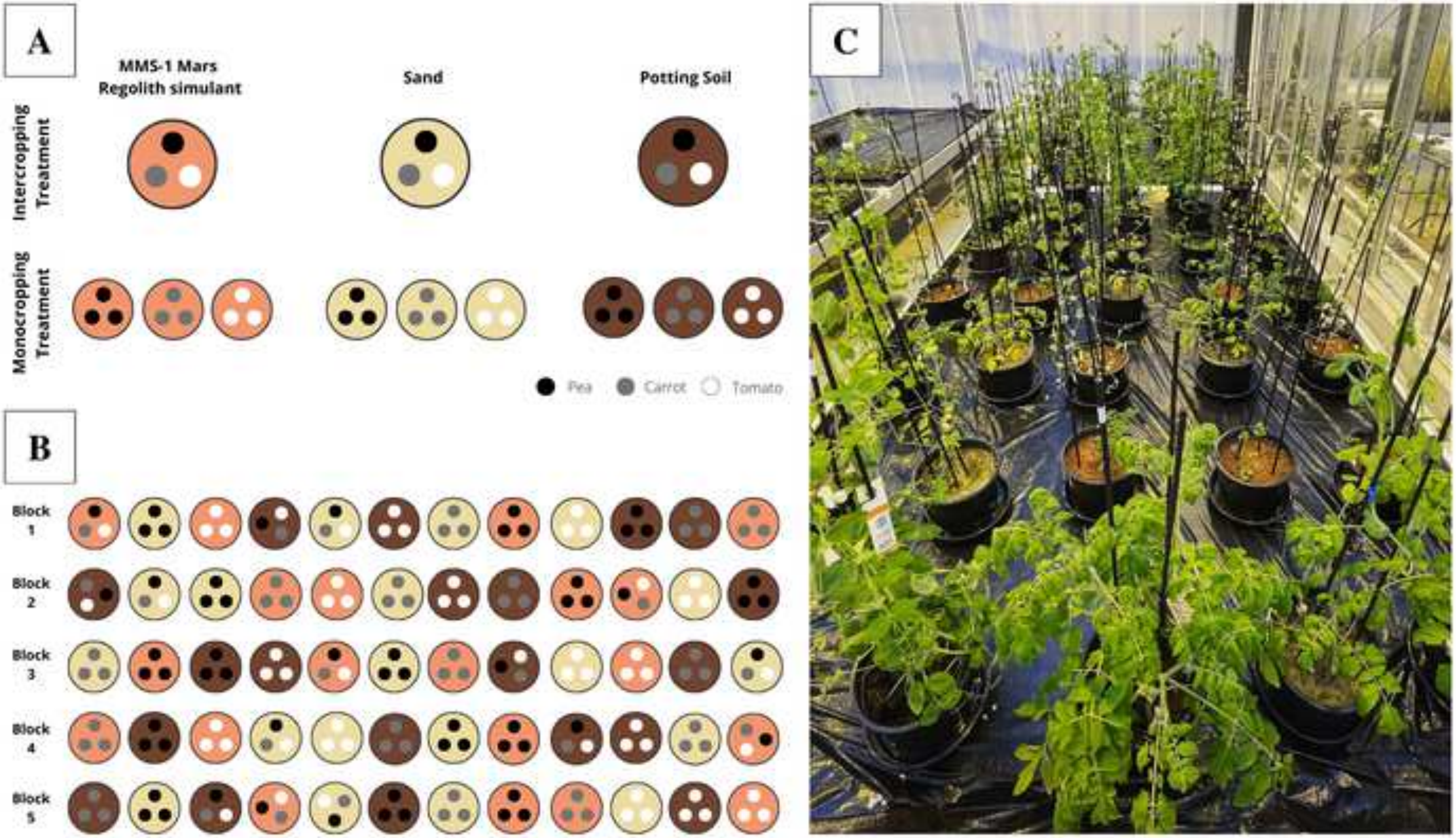

Figure 2

Click here to access/download;Figure;Fig2.tif

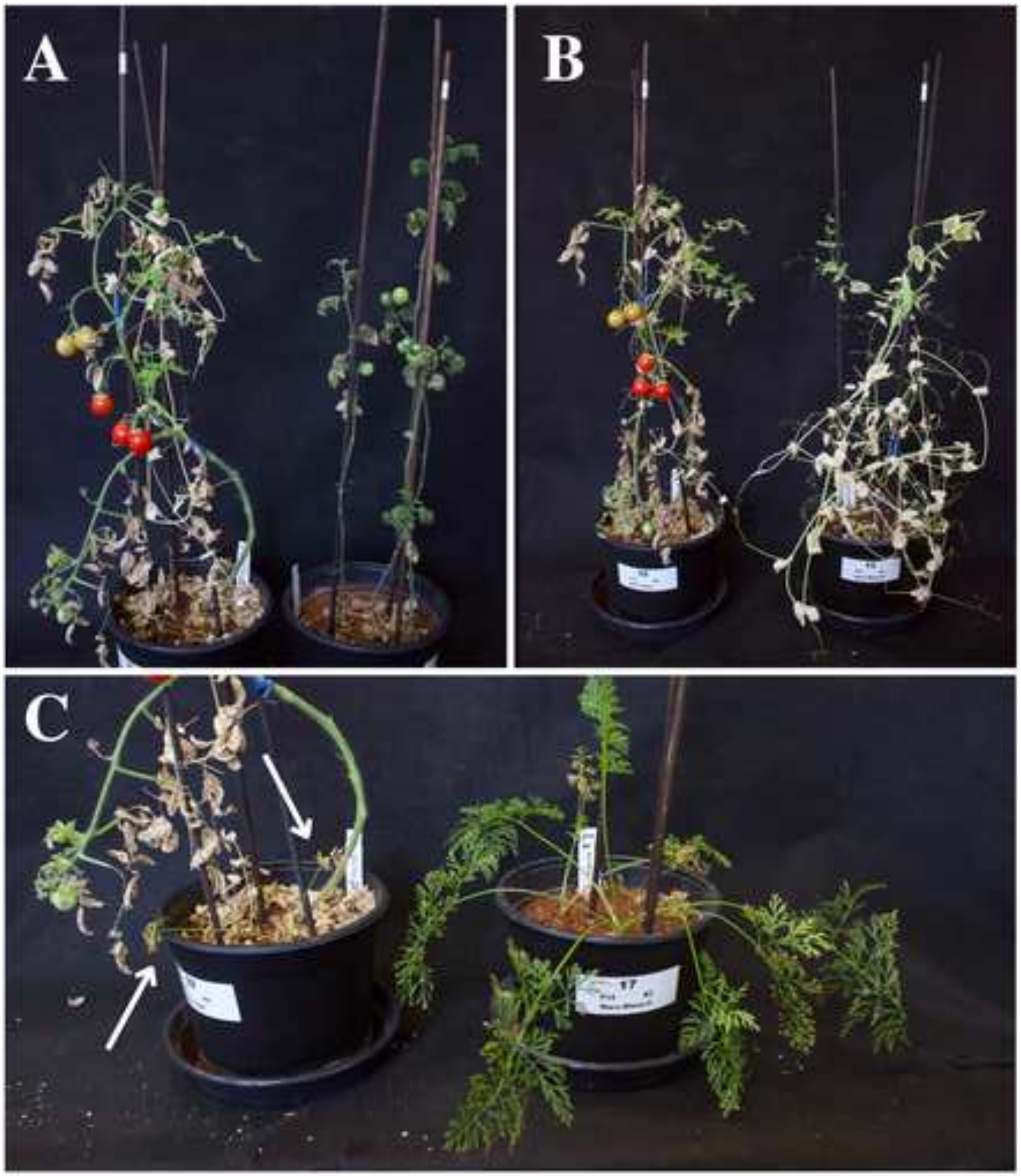

Figure 3

Click here to access/download;Figure;Fig3.tif

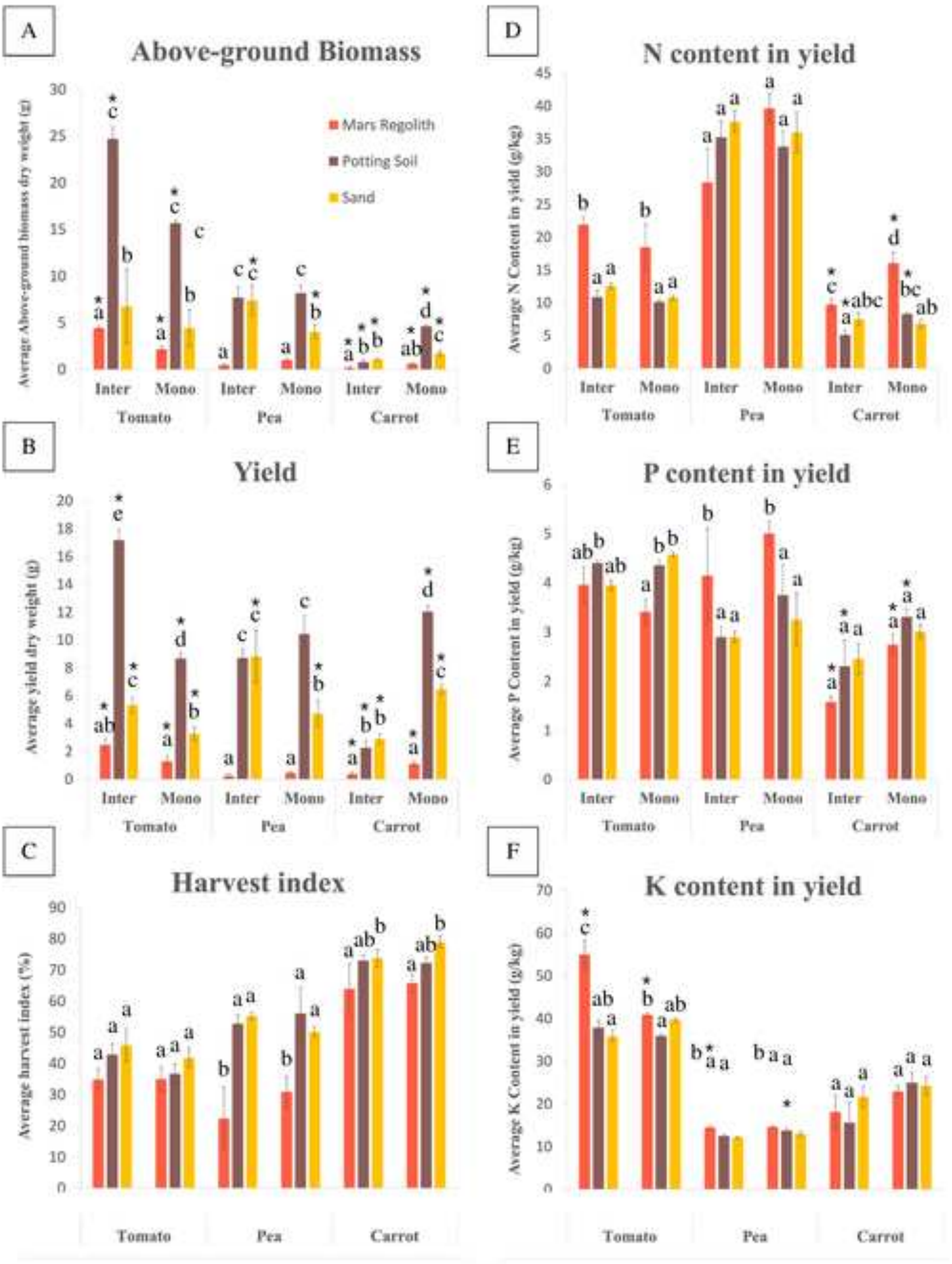

Figure 4

Click here to access/download;Figure;Fig4.tif

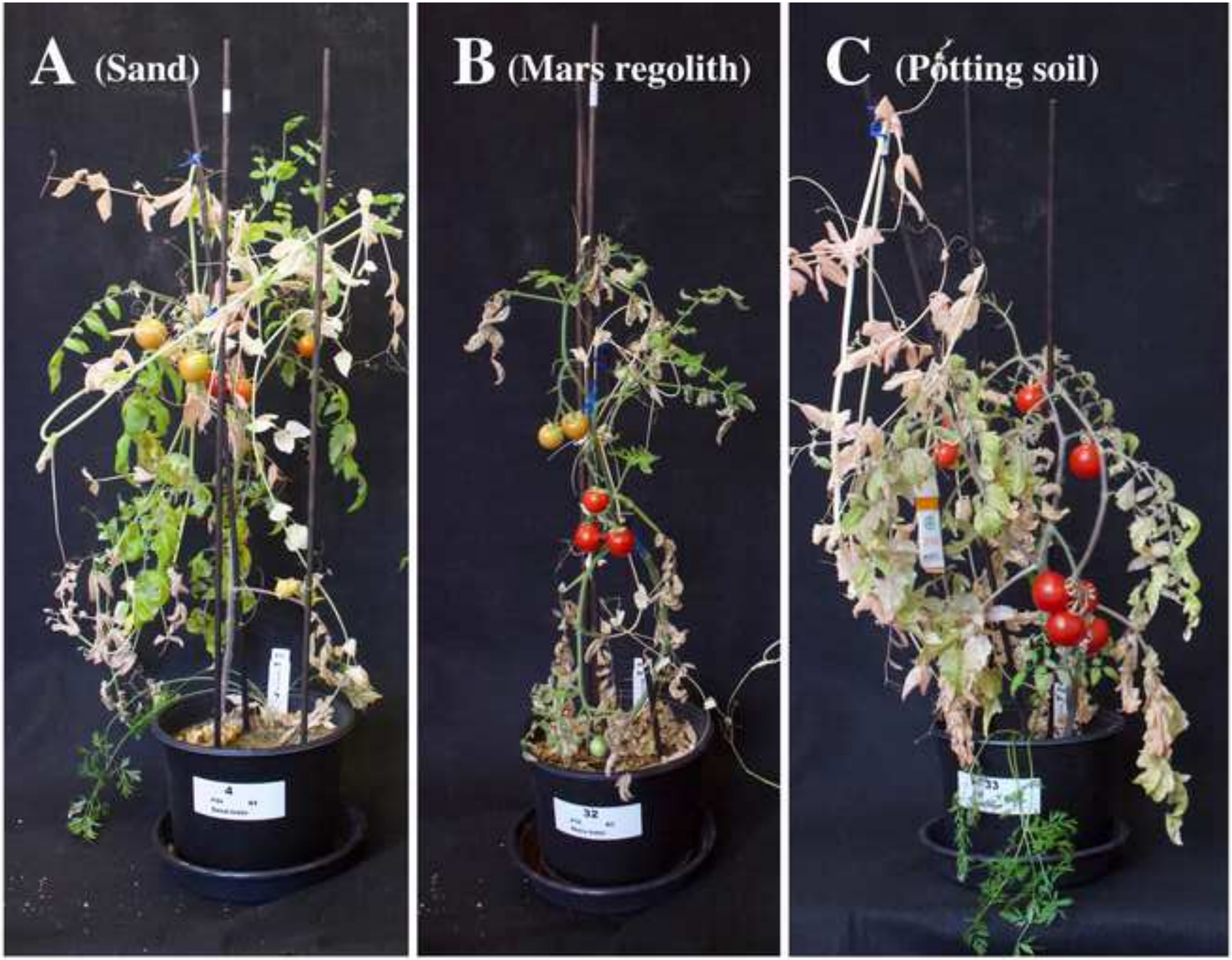

Figure 5

Click here to access/download;Figure;Fig5.tif

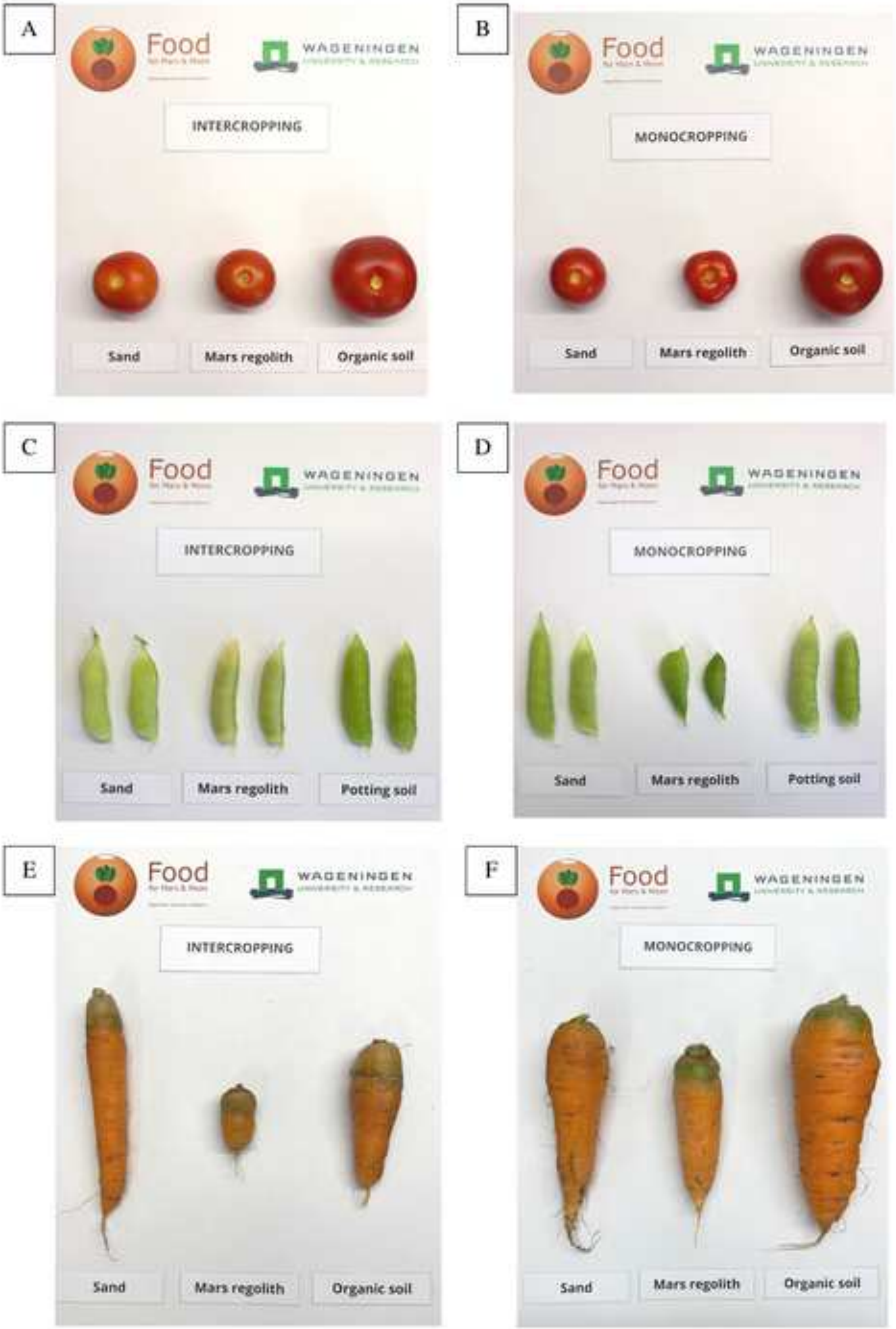

Figure 6

Click here to access/download;Figure;Fig6.tif

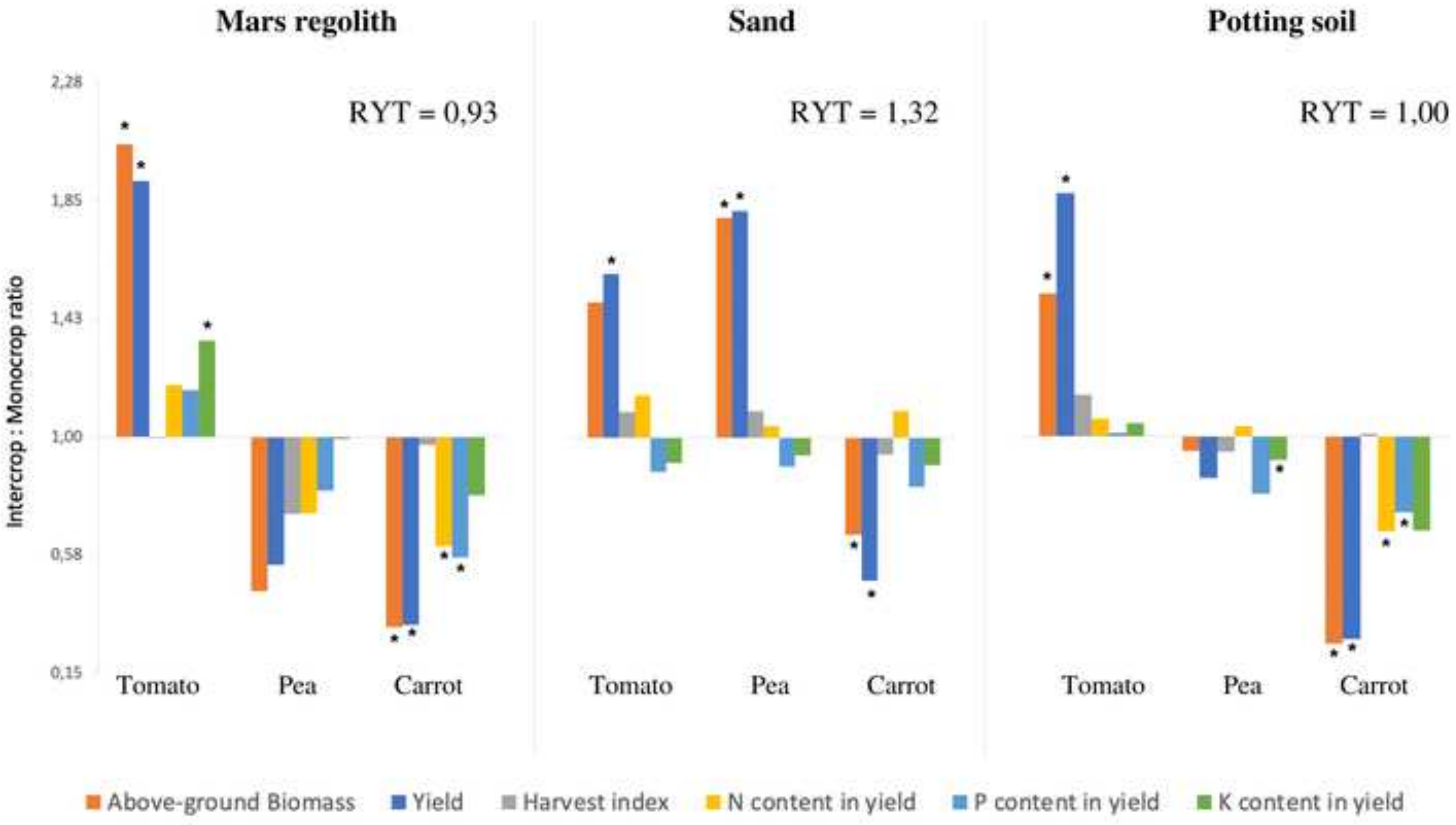

Figure 7

Click here to access/download;Figure;Fig7.tif

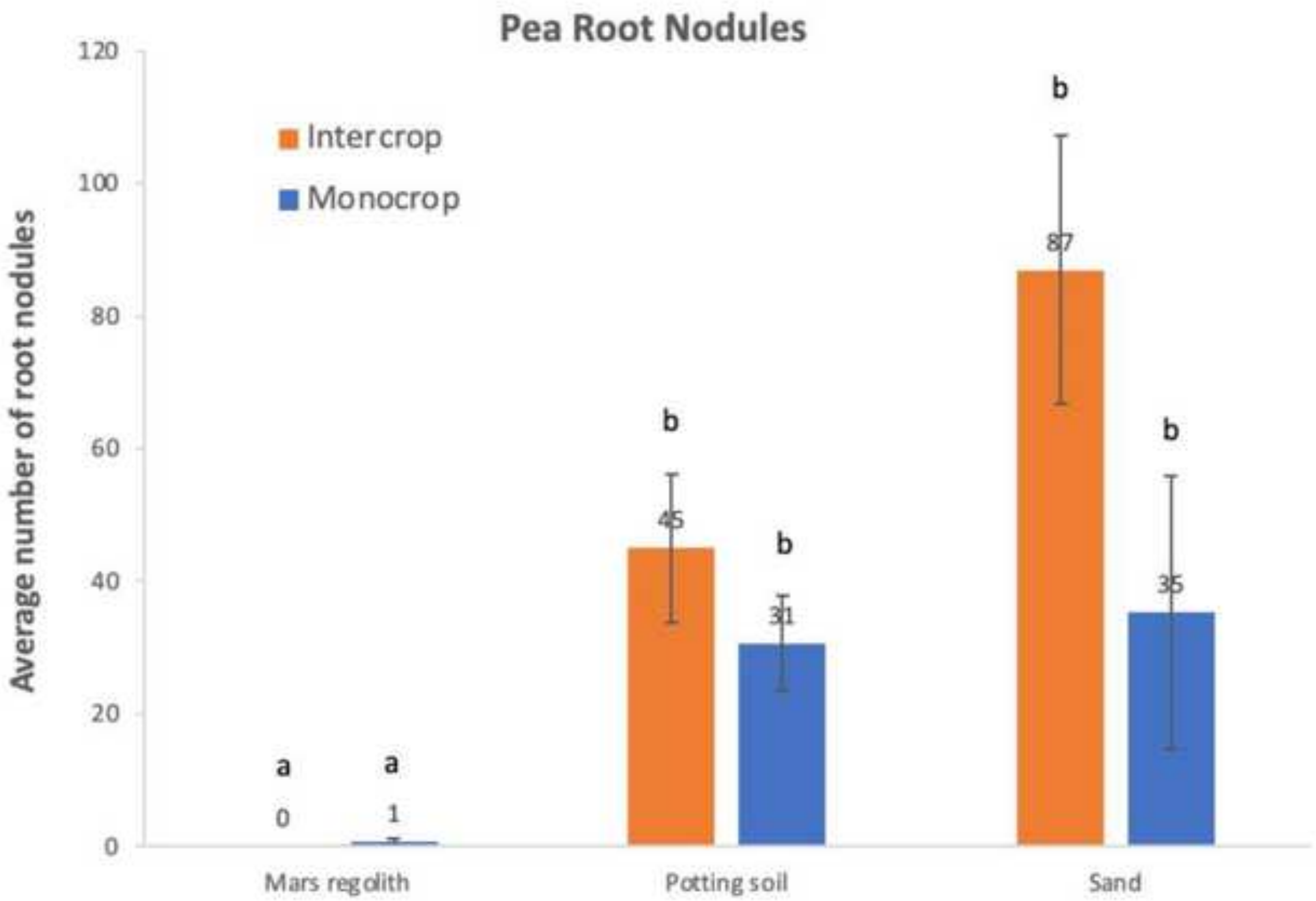

Figure 8

Click here to access/download;Figure;Fig8.tif

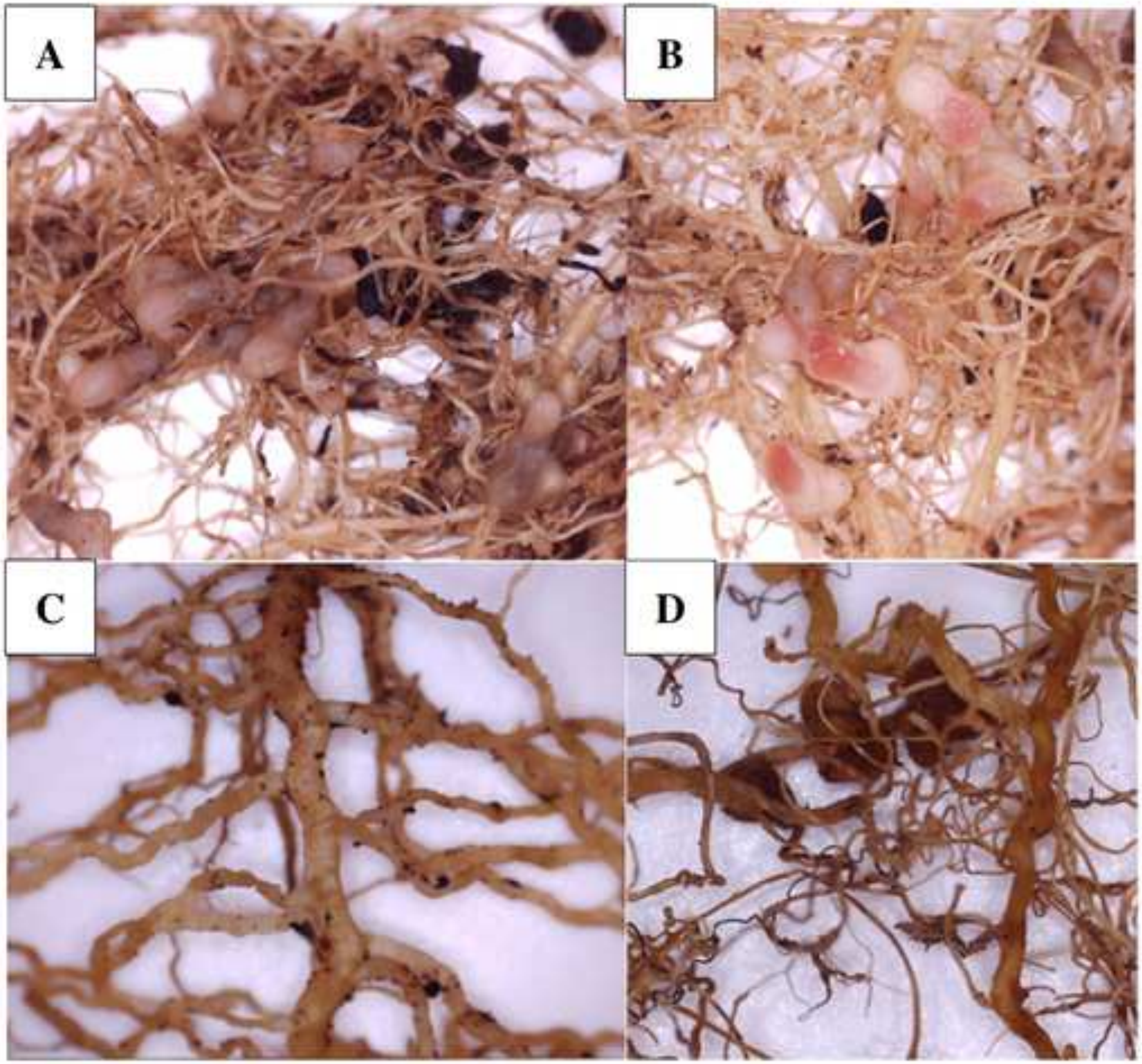

Intercropping on Mars: A Promising System to Optimis~~z~~e Fresh Food Production in Future Martian Colonies

~~Short Title: Intercropping on Mars~~

Rebeca Gonçalves[1]*, G.W. Wieger Wamelink[2], Peter van der Putten[3], Jochem B. Evers[4].

[1] Centre for Crop System Analysis, Wageningen University & Research, Wageningen, The Netherlands

[2] Wageningen Environmental Research, Wageningen University & Research, Wageningen, The Netherlands. Wieger.wamelink@wur.nl

[3] Centre for Crop System Analysis, Wageningen University & Research, Wageningen, The Netherlands

[4] Centre for Crop System Analysis, Wageningen University & Research, Wageningen, The Netherlands

*Corresponding author

E-mail: rebeca.rgoncalves@gmail.com

Abstract

Future ~~human~~ colonists on Mars will need to produce fresh food ~~*in situ*~~locally to acquire key nutrients ~~which are~~ lost in ~~the process of~~ food dehydration, the ~~chief~~ primary technique ~~of~~ for sending food to space. Martian colonies will operate under limited space, energy, and ~~reliability on resupplies from~~ Earth supplies. ~~The aim of this study was~~We aimed to test the viability and prospect of applying an intercropping system as a ~~potential~~ method for soil-based food production in Martian colonies, to optimis~~z~~e ~~the use of~~ resource uses and enhance colony ~~the~~ self-sustainability ~~of the colony~~. A likely early Martian agricultural setting was simulated using small ~~greenhouse~~ pots~~pot~~, a controlled greenhouse environment, and species compliant with space mission requirements. ~~experiment was conducted using three species (P~~Pea (*Pisum sativum*), carrot (*Daucus carota*) and tomato (*Solanum lycopersicum*) were grown in three types of soil ~~-~~ ("MMS-1" Mars regolith simulant, potting soil and sand), planted either ~~together~~ mixed (intercropping) or separate~~ly~~ (monocropping). Rhizobia bacteria (*Rhizobium leguminosarum*) was added as the pea symbiont for Nitrogen-fixing. Plant performance was measured as

above-ground biomass, yield, harvest index, and Nitrogen, Phosphorus and Potassium content in yield. The overall intercropping system performance was calculated as total relative yield (RYT). Intercropping had clear effects on ~~on the~~ plant performance in Mars regolith, being beneficial for tomato but mostly detrimental for pea and carrot, ultimately giving an overall yield disadvantage compared to monocropping (RYT = 0.93). This ~~overall~~ effect ~~was most likely due to~~likely resulted from the observed absence of ~~nodulation by rhizobia bacteria~~Rhizobia nodulation ~~in the pea roots~~ in Mars regolith, negating Nitrogen-fixation and preventing intercropped plants from fully leveraging their complementary properties. Adverse regolith conditions—high pH, elevated compactness and nutrient deficiencies—presumably restricted Rhizobia survival/nodulation. In sand, where more favourable soil conditions promoted extremely effective nodulation, intercropping significantly outperformed monocropping ~~nodulation was the most effective, the intercropping system gave extra yield when compared to monocropping~~ (RYT = 1.32). Given this, we suggest that with simple improvements to the regolith, enhancing conditions for nodulation, intercropping's potential could be effectively unlocked, showing promise for the use of this method to optimise food production in Martian colonies. Specific regolith ameliorations are proposed for future research. ~~Therefore, when peas were optimized for N-fixing in Martian soil, the intercropping system as it was designed gave overall yield advantages, showing promise for the use of this method for optimizing crop growth on Mars. Key features of the MMS-1 simulant were identified for soil amelioration, which can be applied in further research to improve intercropping systems on Mars regolith.~~

Key words: Mixed cropping, resource use efficiency, resource optimization, astrobiology, exobiology

Introduction

As we enter a new age of space exploration, having a permanent settlement on Mars is now a reality in the not-so-distant future. Conquering the red planet is an exciting endeavour, but also one that brings several scientific, economic, and strategic benefits to our own planet and species.

Whilst rovers have begun to pave our way on Mars, the next stage of exploration and research would need to involve sending humans to perform the work themselves, as humans are much more autonomous and efficient at accomplishing field tasks and carrying out research (1)(1). However, having humans on Mars means they need to have their basic necessities covered, and this includes food. To supply and resupply from Earth all the food needs of a long-term settlement is both impractical as well as economically inviable, which means that the crew will have to make use of local Martian resources to produce at least part of their food needs locally (2)(2).

The production of fresh food would in fact be an indispensable factor for any Martian colony. A bioregenerative food system, one where all resources are provided and produced *in situ* with minimal or no resupply from Earth, carries many advantages over the pre-packaged dehydrated foods that astronauts usually eat. As well as having the need for less food to be brought aboard the spacecraft (a factor that also significantly reduces the financial cost of the mission), a chief advantage of fresh food over dehydrated food is the retainment of nutrients essential to human health, especially antioxidants such as vitamin C and β-carotene, both of which are partially reduced or completely destroyed in the process of dehydration (3–5)(3–5). (Guine et al., 2017; Mohammadi et al., 2020; Santos & Silva, 2010). Although supplementary pills could be considered, fresh food provides a much better bioavailability of nutrients in the body, mediated via a complex array of other compounds present (6)(6), (Pressman et al., 2017), and it further decreases the need to rely on resupplies from Earth for essential dietary requirements. The act of growing fresh food itself can also have a great positive impact on the mental well-being of colonists. Research on long-duration space missions, as well as analogue isolation simulations on Earth, have identified psychological and psychosocial factors as two of the most critical problems astronauts will face in their off-world missions (7,8)(7,8). (Kanas et al., 2009; Morphew, 2001). Practising gardening activities has been repeatedly shown to be a significant and effective reliever of stress, anxiety and depression (9,10)(9,10) (Aistis et al., 2022; Clatworthy et al., 2013,), and

Formatted: Font color: Black, English (United States)

Formatted: Font color: Black, English (United States)

Formatted: Font color: Black, English (United States)

Formatted: Font color: Black, English (United States)

astronauts themselves have in the past expressed deep satisfaction at tending to plants in space (11)(11). (Nelson et al., 2008).

Prospects and challenges of Martian agriculture

Several methods for implementing bioregenerative food systems on Mars have been studied, most of which were focused on artificial growing media such as hydroponics and aquaponics. However, soil-based systems, which would involve making use of the available *in situ* Martian regolith, have recently come to new light as having fundamental features for the long-term sustainability of a colony on Mars. Such a system confers many practical and economic advantages over artificial growing media, such as producing higher yields (Barta et al. 2001), offering better buffering capacity for operational errors and technical breaks (12) (Maggi & Pallud, 2010), and harbouring a microbiome environment that could compensate for toxic build-up of trace gases in the growth chamber (11). (Nelson et al., 2008). Moreover, inedible crop parts and food waste, as well as human waste, can be composted and added to the soil to provide nutrients for the next harvest. This adds a circularity factor to the system that would minimize, and eventually eliminate, any need for expensive resupplies of nutrients from Earth (13).(Silverstone et al., 2003).

Gardening on Mars *is* viable. Although Mars has a very thin atmosphere (1% that of Earth), it contains the necessary elements for plant growth, such as carbon dioxide (CO_2) and nitrogen (N_2) (14) (ESA, 2018), and the planet is abundant in (frozen) water present both in the polar caps and in several places under the Martian surface (15) (Orosei et al., 2020). Because of its thin atmosphere, Mars has high radiation levels on the surface which can be harmful to both plants and humans (16) (Tack et al., 2021), and the planet holds an average global temperature of -65°C (17) (NASA, 2022). To counteract these conditions, colonies on Mars will likely consist of closed habitats that can shield both plants and humans from harmful radiation, and that can maintain the optimum temperature and atmospheric conditions for life within the colony (18) (Liu et al., 2022). Gravity on Mars is about 38% that of Earth (17)(NASA, 2022). However, experiments done on plant growth under microgravity conditions aboard the International Space Station (ISS) showed that plants, including crop species such as lettuce, cabbage and mustard, could be successfully grown, harvested and safely consumed by astronauts (19) (Mariani, 2020), showing promise that the lower gravity will not be a major impediment for normal plant development on Mars.

Formatted: Font color: Black, English (United States)

Formatted: Font color: Black, English (United States)

Formatted: Font color: Black, English (United States)

Formatted: Font color: Black, English (United States)

Formatted: Font color: Black, English (United States)

Formatted: Font color: Black, English (United States)

Formatted: Font color: Black, English (United States)

Formatted: Font color: Black, English (United States)

Formatted: Font color: Black, English (United States)

Formatted: Font color: Black, English (United States)

Formatted: Font color: Black, English (United States)

There are some potential obstacles with the Mars regolith that need to be overcome in order to successfully grow plants using *in situ* resources. For example, samples from Mars missions have shown the presence of heavy metals and perchlorates in the Martian regolith across different landing sites (20), harmful to both plants and humans. However, phytoremediation and bioremediation offer potential solutions to this, where specific plants or microbes can be cultivated to remove such elements from the soil with up to 100% efficacy (21–23). Moreover, there's the suggestion that perchlorates would not be present in deeper layers of Martian regolith, since their formation on Mars is proposed to be largely due to the regolith interaction with cosmic radiation and other atmospheric processes (24). Additional concerns include the regolith's high pH (~8.5) and nutrient deficiency (especially of nitrogen), both detrimental to plant growth and health. To address these, the addition of compost to the soil could lower the pH (25) while also providing the lacking nutrients. An initial batch of fertilizer could be incorporated in first missions' cargo supply, until a cyclic bioregenerative system could be established. Such system could use inedible parts from the crops grown *in situ* as compost for the next crop generation, or make use of human faeces as manure (26).

Optimizing resource use in Martian colonies

On Earth, it is possible to test the viability of using Martian soil (and thus of having a Martian soil-based system) for crop growth using Mars regolith simulants available. Several crop growth experiments have been successfully carried out on Earth using such simulants, with over 20 different species of crops reported to be able to grow and produce yield (27–29). (Ramírez et al., 2017; Wamelink et al., 2014; Wamelink et al., 2019). However, the main focus of these pioneering experiments was to test whether plants *could* germinate, grow and produce yield in the Mars regolith. No attention has yet been given to developing the *efficiency* of the system. Since colonies on Mars will probably operate under a limited amount of energy, space and other resources, any crop growth method that increases the productivity and circularity of the system, in a way that optimizes the use of such space and resources, will be paramount to the success and viability of the mission.

Intercropping is an ancient technique used on Earth that involves growing two or more crop species simultaneously in the same field (30) (Mt.Pleasant, 2016). This differs from the "traditional" monocropping system, where only a single crop species is planted in the same field. Intercropping has long been known to produce several beneficial effects compared to monocropping, such as increasing plant biomass and yield of one or more of the component species, improving the nutritional value of

Formatted: Font: Italic

Formatted: Font: Italic

Formatted: Font color: Black, English (United States)

Formatted: Font color: Black, English (United States)

Formatted: Font color: Black, English (United States)

Formatted: Font color: Black, English (United States)

Formatted: Font color: Black, English (United States)

Formatted: Font color: Black, English (United States)

Formatted: Font color: Black, English (United States)

yield, and optimizing the use of resources in the system (31–33). ~~(Jensen et al., 2020; Latati et al., 2016; X. Li et al., 2013; Jensen et al., 2020).~~ Such effects have been observed both in the field as well as in greenhouse pot experiments (34–36). ~~(Nasar et al., 2020; Patra & Subbiah, 1986; Wu et al., 2016).~~ The beneficial effects of intercropping are often measured as a more efficient use of resources compared to the performance of each separate species in a monocropping system.

One way intercrop productivity benefits can arise is when the interspecific competition (competition between different species) in the intercropping system is *lower* than the intraspecific competition (competition amongst the same species) (37) ~~(Hauggaard-Nielsen et al., 2001)~~. Another way is by means of *species complementarity*, where both (or all) intercropped species can mutually benefit each other within the system. Complementarity can work either by 1) *niche differentiation*, where companion species that are adapted to different niches (i.e. a shallow root species together with a deep root species) can make a more complete use of all available resources in the system, resulting in less competition for resources; or by 2) *resource facilitation*, where one companion species provides or facilitates the up-take of a limiting resource to the other companion species (38) ~~(Bybee-Finley & Ryan, 2018)~~. A classic example of resource facilitation in intercropping is seen through legumes, which can provide nitrogen (N) into the intercropping system thanks to their symbiotic relationship with rhizobia bacteria. The bacteria infect the legume´s roots to form "root nodules", wherein they turn atmospheric nitrogen (N_2) into the usable form of ammonia (NH_3) via a process called N fixation (39) ~~(Stern, 1993)~~. This process helps relieve the competitive pressure on the intercropping system, either via the addition of N in soil (40) ~~(Gatsios et al., 2019)~~ or via direct N transfer from the legume to the non-legume (41) ~~(Salgado et al., 2021)~~, and often results in the optimization of resources such as lower N fertilizer requirements (31)~~(Jensen et al., 2020)~~.

The benefits of intercropping towards optimizing yield and resource use make it a prime candidate method to be applied on a soil-based system on Mars. To date, no experiments have ever been done to investigate the effects of intercropping on crops grown in Mars regolith. In this study, we aim to test the viability and prospect of implementing an intercropping system as a potential food production method in future Mars colonies.

Formatted: Font color: Black, English (United States)

Formatted: Font color: Black, English (United States)

Formatted: Font color: Black, English (United States)

Formatted: Font color: Black, English (United States)

Formatted: Font color: Black, English (United States)

Formatted: Font color: Black, English (United States)

Formatted: Font color: Black, English (United States)

Formatted: Font color: Black, English (United States)

Study objective and research questions

Our study objective is to investigate the effects of intercropping on crop performance grown in a Mars regolith simulant (from now on also referred to as just “Mars regolith”). Here we quantify plant performance as above-ground biomass, yield, harvest index, and nutritional content of yield as levels of nitrogen (N), phosphorus (P) and potassium (K). We chose three companion species, namely pea (*Pisum sativum*), carrot (*Daucus carota*) and tomato (*Solanum lycopersicum*), for their complementary and nutritional properties, and for the fact that they have all been previously shown to be able to grow and produce yield in Mars regolith simulant (29) (Wamelink et al., 2019). Nodulation by rhizobia bacteria in pea roots will also be a key variable observed in this experiment, to interpret the effects that an N-fixing species would have on the interspecific competition within the intercropping system.

Our main research question was whether we would see any beneficial effects of intercropping on the performance of pea, carrot and tomato grown in Mars regolith simulant. If plants perform better when intercropped, by making use of the exact same resource input as when they are monocropped, this could prove the efficacy of intercropping as a method for increasing the efficiency of the system.

Formatted: Font color: Black, English (United States)

~~Additionally, we will test whether pea root nodulation by rhizobia bacteria (*Rhizobium leguminosarum*) can occur in the specific Mars regolith simulant used in this experiment (MMS-1). Previous research have successfully seen nodulation in MMS-1, but only for the bacteria species *Sinorhizobium meloti* and *Sinorhizobium medicae* (Rainwater & Mukherjee, 2021).~~

Materials and Methods

Greenhouse conditions

We performed a pot experiment in a greenhouse on Earth, in Wageningen (51.9692° N, 5.6654° E). During the experimental period, average temperature in the greenhouse was 20.4 ± 1.7°C, relative humidity was 60.9 ± 15%, and day/night cycle was 16/8h. Ambient air was used and no extra CO_2 was added. Lamps yielding 600 watt (HPS 230 volt) were switched on if sunlight intensity was below 150 watt/m^2, and switched off if sunlight intensity was above 250 watt/m^2.

Formatted: English (United States)

Mars regolith simulant

The regolith analogue used in this experiment was the MMS-1 Mars Regolith Simulant, of unsorted grade (grain <3.17mm, density 1.25g/cm^3), provided by The Martian Garden Company (Austin, USA). This simulant was manufactured under commission by researchers at the National Aeronautics and Space Administration (NASA)'s Jet Propulsion Laboratory (JPL) (42). ~~(Peters et al., 2008).~~ MMS-1 was designed to be the closest simulate of the regolith found on the Martian surface, especially when in contact with water. For a comparison of physical and chemical properties of the MMS-1 simulant and actual Martian regolith see Appendix 1.

Formatted: Font color: Black, English (United States)

Earth soils and soil preparation

Two "Earth" soils were included in the experimental design: (1) common potting soil, composed mainly of peat with fertilizer (PGmix 15%N-10%P2O5-20%K2O), and (2) river sand. The potting soil was sieved to remove lumpy matter and improve homogeneity. Sand was specially chosen for it being nutrient poor similar to Mars regolith (see also (28) ~~Wamelink et al. 2014~~), to test if they would produce comparable results.

Formatted: Font color: Black, English (United States)

For the Mars regolith and the sand treatments, a small amount of organic soil (derived from the sieved potting soil) was mixed at 10% of the total volume, to improve root aggregation and water retention (43)~~(Caporale et al., 2020)~~. Table 1 shows the pH and Nutrient analysis from samples of the three soils.

Table 1. Nutrient composition and pH for the three soil treatments (MMS-1 Mars regolith simulant, sand and potting soil), collected at the start of the experiment (day 0). Results shown for Mars regolith simulant and sand include a 10% volume of organic matter mixed with each.

**Nutrient (mg/kg)**	**Mars regolith simulant (MMS-1)**	**Sand**	**Potting soil**
N-NH4	4,34	3,71	343,16
P-PO4	0,1	0,1	41,77
N-NO3	15,94	5,27	180,70
Nmin	20,3	9	523,9
pH	8.5 ± 0.2	7.5 ± 0.2	5.5 ± 0.2

Species selection

The species selected for this experiment were pea (*Pisum sativum* cv. 'Prince Albert', Tuin Plus), carrot (*Daucus carota* cv. 'Nantes 2', Buzzy), and dwarf cherry tomato (*Solanum lycopersicum* cv. 'Tiny Tim', Buzzy). These species represent 3 out of the 27 potential food candidates to be incorporated into a space habitat, as proposed in NASA's Life Support Values and Assumptions Document (44) ~~(Anderson et al., 2015)~~, based on various selection criteria such as potential yield, harvest index, horticultural requirements and macronutrient content needed to supplement a crew's diet.

These species have also been chosen for their particular complementary properties which can be mutually beneficial to each other. As a legume, pea can serve as N-fixers in the system in symbiosis with rhizobia bacteria. ~~(*Rhizobium leguminosarum*).~~ Carrot helps aerate the soil, which can improve water and nutrient uptake by companion plants (45) ~~(Lipiec et al., 2003)~~. Tomato can provide shade for the temperature sensitive carrot and support for the climbing pea (46) ~~(Riotte, 1998)~~, and both carrot and tomato release root exudates such as flavonoids that can promote root nodule formation in pea (47) ~~(Leoni et al., 2021)~~. Furthermore, all three species have compostable crop waste that can be mixed with

Formatted: Font color: Black, English (United States)

Formatted Table

Formatted: English (United States)

Formatted: Font color: Black, English (United States)

Formatted: Font color: Black, English (United States)

Formatted: Font color: Black, English (United States)

Formatted: Font color: Black, English (United States)

the soil to provide key nutrients to the subsequent crop, reducing the need for fertilizers and promoting circularity in the system (48) ~~(Ho et al., 2020)~~.

Finally, these species were chosen for their nutrient composition. All three species, in particular carrot and tomato, are high in antioxidants such as vitamin C and β-carotene (49–51). ~~(Ali et al., 2021; Arscott & Tanumihardjo, 2010; Kumari & Deka, 2021).~~ These are an essential part of the human diet, but most importantly are amongst the few key nutrients that are mostly or completely lost in dehydrated foods (4).~~(Mohammadi et al., 2020).~~

Rhizobia bacteria addition

To provide the peas with their symbionts for N-fixing, 0,75ml of *Rhizobium leguminosarum* (biovar *viciae* strain 248 OD600 ~ 0.784) was added to every pot, including pots not containing pea plants. Inoculation was done 19 days after sowing. The selection of *R. leguminosarum* was based on its well-documented symbiotic relationship with *P. sativum* (52). This particular strain was chosen due to its prior successful nodulation with *P. sativum* in a previous in-house experiment, where the same Mars regolith simulant (MMS-1) was used, thus aiming to control the potential variable of the bacteria not adapting to Martian regolith conditions.

Experimental design

We used a randomis~~z~~ed complete block design with the three experimental species (pea, carrot and tomato) grown in three types of soil (MMS-1 Mars regolith simulant, sand and potting soil), divided into two cropping systems (intercropping and monocropping). This gave 12 different treatments with five replicas for each treatment, resulting in 60 pots. The experimental design and treatments can be seen in Fig 1.

Formatted: Font color: Black, English (United States)

Formatted: Font color: Black, English (United States)

Formatted: Font color: Black, English (United States)

Formatted: Font: Italic

Formatted: Font color: Black, English (United States)

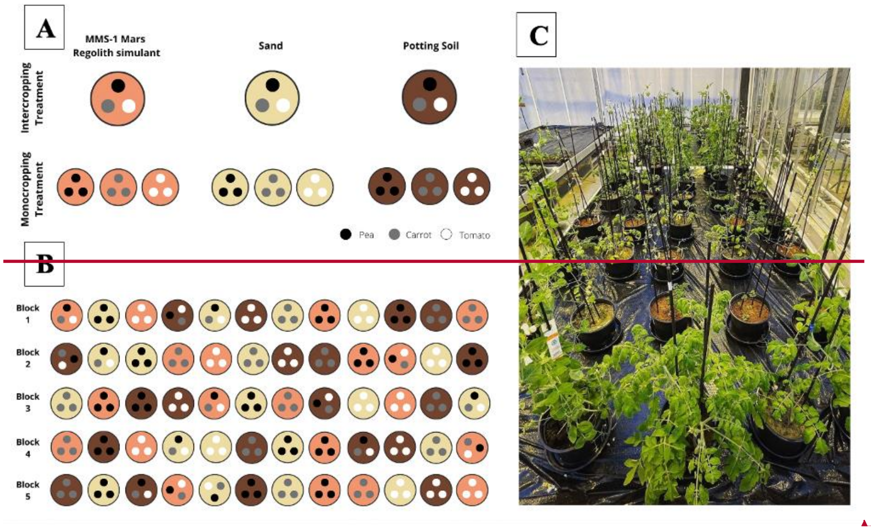

Fig 1. Experimental design. A: Overview of experimental treatments. B: Randomised complete block design with five replicas, and intercropping treatment pots randomly rotated. C: Photo of the experiment (day 48).

For the monocropping treatments, three plants of the same species were grown in each pot, whilst for the intercropping treatments, one plant of each species was grown in each pot. 5L Pots were used, 16cm deep and 20cm wide at the top. Pots were filled to a 13cm depth, with 4.55Kg Mars regolith, 5Kg sand and 1.3Kg potting soil. A mesh was placed on the bottom of each pot to keep the soil in place. Stakes were placed to provide climbing support for the peas, with three stakes placed in every pot.

Seeds were sown on 1st December 2021 and the experiment ran for 105 days, with the harvest taking place on 16th March 2022. Seeds were over-sown to ensure the required germination. Thinning of germinated seeds was carried out 10-14 days later. The configuration of the intercropping sowing was always the same, but pots were then randomly rotated, so that the final configuration was fully random.

Watering and nutrient solution

Formatted: English (United States)

Water saturation point for each soil was measured before the start of the experiment (Mars regolith = 35.7%, potting soil = 119%, sand = 27%). Watering was done once a day, every day, by pouring water on the saucer beneath the pot. For this, a random sample of three pots from each soil type was weighed and the amount of water to be given was determined to be just below the saturation point for that soil. Since the soils had different water retention properties, the amount of water needed per soil differed, and this was done in order to ensure that every treatment received the same amount of water relative to its saturation point.

100ml of 2EC Hoa~~o~~gland nutrient solution was given once a week for the first 68 days, then increased to twice a week for the remainder of days to compensate for the increased biomass from the growing plants. The solution was poured on the topsoil surface. Solution contents can be found in Appendix 2.

Harvesting and Measurements

At harvest, above-ground biomass and yield were weighed for all three species. Both variables were measured in grams per plant (for monocropping treatments these values were added and divided by the number of plants in the pot, so to get the average value per pot). Above-ground biomass included stem, leaves and branches. Yield fresh weight was done separately for ripe and immature tomato fruits, and for pea pods and pea seeds, where only the seeds were considered for yield analysis. Harvesting of pea yield was also done throughout the experiment, as the first pea seeds started to mature on day 65. The first tomato fruits only started to ripen two weeks before the harvest date, so they were left on the plants to be harvested all at once. For carrot yield, the whole carrot from the crown to the tap root was weighed. All pea roots were washed and visually assessed for the presence of rhizobia nodules, which were also dissected and checked for nodule activity using a light microscope (active nodules show an inner pink coloration (53)) ~~(Schumpp & Deakin, 2010)).~~

Formatted: Font color: Black, English (United States)

All fresh weighed samples were put in ovens to dry at 70°C for 48 hours.

Yield of the three species was analysed for NPK content. Three replicas of each treatment were grinded and analysed using digestion $H_2SO_4/H_2O_2/Se$ + NP on SFA and $H_2SO_4/H_2O_2/Se$ + K on ICP-OES. For tomato, only ripe (red) tomato fruits were analysed.

Calculations

Harvest index (HI) is a measure of the reproductive efficiency, indicating how much of a plant's above-ground biomass is allocated to yield. It was calculated from dry weight data, for all species, according to equation 1:

$$HI\,(\%) = \frac{Y}{Y + ABG} * 100 \qquad (1)$$

Where Y is the yield (g), and ABG is the above ground biomass (g).

The division of the intercropping yield over the monocropping yield of the same species gives the relative yield (RY) for that species (equation 2). A RY of > 0.33 (0.33 being the ratio of each species present in the intercropping treatment) indicates that the species over-yielded in the intercropping treatment compared to its monocropping treatment.

$$RY = \frac{Y_i}{Y_m} \qquad (2)$$

Where Y_i is the yield from the intercropping treatment (per species) (g), and Y_m is the yield from the monocropping treatment (averaged between the number of plants in the pot) (g).

Relative yield total (RYT) is an index to assess whether there was any yield advantage in the intercropping system compared to monocropping. It is a useful measure to assess how efficiently the intercropping system performed as a whole (54). (Fetene, 2003). It was calculated according to equation 3:

Formatted: Font color: Black, English (United States)

$$RYT = RYt + RYp + RYc \qquad (3)$$

Where RYt, RYp and RYc are the relative yields of tomato, pea and carrot (respectively). If RYT > 1, this indicates relative yield (and hence resource use) advantage from intercropping, and RYT < 1 indicates relative yield disadvantage from intercropping.

Data analysis

All data was checked for normality and homogeneity using a W test for normality and a Barlett's test for homogeneity. When needed, LOG_{10} transformations were done to meet the two assumptions, and tests were then run on the transformed data. There were only three parameters that did not fit homogeneity, tomato yield P content (P=0.024), and harvest index for peas and for carrots (P=0.011), but those were still included in the analysis.

A two-way ANOVA was performed on the following variables: above-ground biomass dry weight, yield dry weight, harvest index, N content in yield (NY), P content in yield (PY) and K content in yield (KY). For tomato yield, the data also included the dry weight of the immature fruits harvested, since they would become ripe and consumable if harvested later. The ANOVA was run separately for each species, with cropping type and soil type at the interaction level, and block effects accounted for. A post-hoc Fisher's protected LSD test ($P<0.05$) was performed for significant effects of soil type and the interaction between soil type and cropping type.

An analysis of deviance was performed on the ratios between intercropping and monocropping for each species in every soil (calculated from LOG-transformed data), and the p-values were obtained for the null hypothesis that the ratio equals 1 (i.e. no difference between intercropping and monocropping treatments).

There was a missing value in one of the replicas sent for the NPK analysis of pea yield from the intercropping Mars regolith treatment due to insufficient pea material available. This means that for this treatment, only 2 replicas were analysed for NPK. There were no other missing or deleted values in the data set.

Results

Mars regolith

Tomato showed increased performance in the intercropping treatment compared to the monocropping treatment, with significantly higher above-ground (AG) biomass (P=0.037), yield (P=0.043), and Potassium content in yield (KY) (P<0.001, Table 2, Fig 2 and Fig 3).

Contrary to tomato, carrot showed decreased performance in the intercropping treatment compared to the monocropping treatment, with significantly lower AG biomass (P=0.023), yield (P=0.022), NY (P<0.001) and PY (P=0.011, Table 2).

Pea showed no significant difference in performance between the intercropping and monocropping treatments for any of the variables. However, like carrot, the observed trend showed lower values in the intercropping treatments compared to the monocropping treatments for all variables, with the p-value for N content close to the threshold (P=0.060, Table 2).

There was no significant difference in harvest index between intercropping and monocropping for any of the three species.

Table 2. Average values (N=5) of all six measured variables for tomato, pea and carrot for the intercropping and monocropping treatments, for each of the three soils (MMS-1 Mars regolith simulant, sand and potting soil).

	**Tomato**		**Pea**		**Carrot**	
	**Intercropping**	**Monocropping**	**Intercropping**	**Monocropping**	**Intercropping**	**Monocropping**
**AG[1] Biomass (g)[2]**						
Mars regolith	4.42 (±0.23)** a	2.15 (±0.35) a	0.45 (±0.10) a	1.01 (±0.14) a	0.19 (±0.08)** a	0.60 (±0.13) ab
Sand	6.76 (±3.92) * b	4.45 (±2.00) b	7.41 (±1.59)** c	4.01 (±0.74) b	1.07 (±0.06)** b	1.71 (±0.30) c
Potting soil	24.71 (±1.26)** c	15.68 (±0.28) c	7.72 (±1.11) c	8.18 (±0.88) c	0.79 (±0.26)*** b	4.62 (±0.15) d
**Yield (g)[2]**						
Mars regolith	2.47 (±0.41)** ab	1.29 (±0.37) a	0.25 (±0.14) a	0.46 (±0.08) a	0.36 (±0.15)** a	1.12 (±0.19) a
Sand	5.32 (±0.56)** c	3.26 (±0.45) b	8.82 (±1.89)** c	4.71 (±0.94) b	2.89 (±0.38)*** b	6.46 (±0.37) c

Potting soil	17.18 (±0.76)*** e	8.68 (±0.40) d	8.72 (±0.61) c	10.44 (±1.30) c	2.26 (±0.53)*** b	12.04 (±0.43) d
**Harvest index (%)**						
Mars regolith	34.84 (±3.44) a	34.98 (±3.82) a	22.34 (±10.20) b	30.93 (±4.79) b	63.92 (±8.17) a	65.75 (±2.74) a
Sand	45.85 (±5.31) a	41.73 (±3.24) a	55.24 (±1.20) a	50.17 (±1.65) a	73.80 (±2.73) b	78.98 (±1.90) b
Potting soil	42.80 (±3.41) a	36.66 (±3.22) a	52.74 (±2.86) a	56.07 (±8.29) a	73.04 (±1.62) ab	72.25 (±1.91) ab
**N content in yield (g/kg)**						
Mars regolith	21.93 (±1.16) b	18.47 (±3.51) b	28.35 (±5.10)* a	39.63 (±2.26) a	9.73 (±0.88)*** c	16.03 (±1.64) d
Sand	12.57 (±0.39) a	10.80 (±0.32) a	37.60 (±1.63) a	35.97 (±3.08) a	7.50 (±0.95) abc	6.80 (±0.62) ab
Potting soil	10.87 (±1.01) a	10.13 (±0.34) a	35.20 (±2.43) a	33.80 (±2.33) a	5.13 (±0.74)** a	8.27 (±0.24) bc
**P content in yield (g/kg)**						
Mars regolith	3.96 (±0.37)* ab	3.41 (±0.25) a	4.15 (±0.96) b	5.01 (±0.24) b	1.58 (±0.11)** a	2.74 (±0.22) a
Sand	3.95 (±0.11)* ab	4.58 (±0.05) b	2.90 (±0.11) a	3.25 (±0.54) a	2.45 (±0.32) a	3.01 (±0.14) a
Potting soil	4.40 (±0.04) b	4.36 (±0.11)b	2.90 (±0.20) a	3.75 (±0.62) a	2.30 (±0.52)** a	3.31 (±0.17) a
**K content in yield (g/kg)**						
Mars regolith	55.00 (±3.29)*** c	40.87 (±0.44) b	14.50 (±0.49) b	14.60 (±0.25) b	18.13 (±3.94) a	22.93 (±1.26) a
Sand	35.90 (±1.36)* a	39.70 (±0.38) ab	12.10 (±0.20) a	13.00 (±0.57) a	21.67 (±2.50) a	24.27 (±2.09) a
Potting soil	37.87 (±1.58) ab	35.93 (±0.49) a	12.47 (±0.32) ** a	13.77 (±0.49) a	15.60 (±4.74)* a	24.97 (±2.45) a

1. "AG" = Above-ground

2. Values refer to dry weight

Values in brackets refer to standard error (SE)

Asterisks (*) refer to significant differences between intercropping and monocropping treatments *within* each species from the *same* soil treatment, at the 0.1 level (*), 0.05 level (**) and 0.001 level (***) (Analysis of Deviance).

Letters refer to differences between *soil* treatments within each *variable* for each species. Different letters indicate a significant difference at the 0.05 level (Fisher's LSD test)

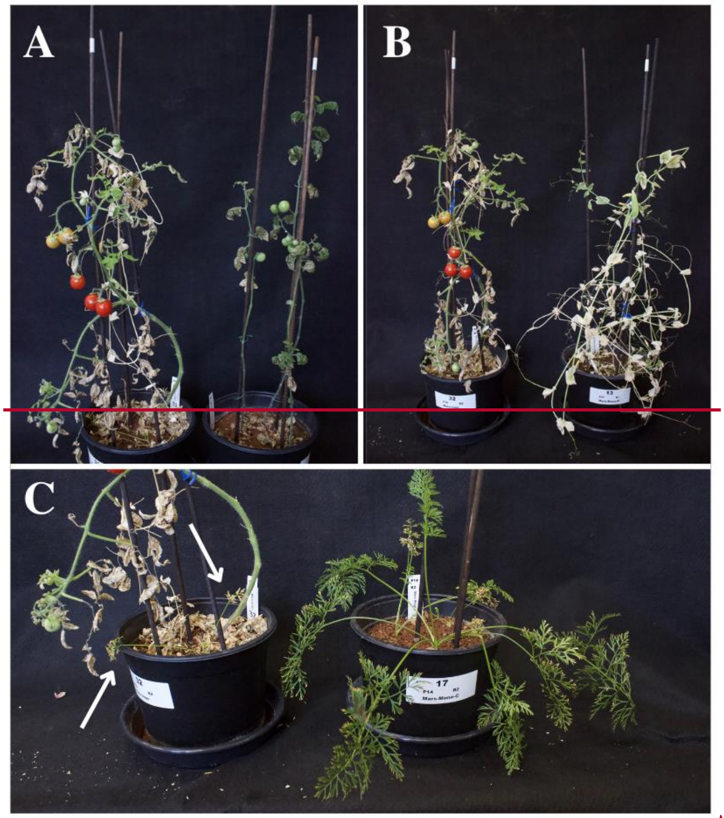

Fig 2. Comparison between intercropping and monocropping treatments in Mars regolith simulant. A: intercropping treatment (left) and monocropped tomato (right). B: intercropping treatment (left) and monocropped pea (right). C: intercropping treatment (left) and monocropped carrot (right). Arrows point to the small carrot leaves from the intercropping treatment. Pictures were taken on the day of harvest (day 105).

Comparison between soils

Potting soil produced the same pattern of results as those seen in the Mars regolith simulant, when comparing the intercropping and monocropping treatments (Table 2 and Fig 3). The only exception was

Formatted: English (United States)

KY, where in tomato it showed no significant difference, and in pea it was significantly lower in intercropping compared to monocropping (P=0.029).

Sand also produced a similar pattern of results to Mars regolith, when comparing the intercropping and monocropping treatments (Table 2 and Fig 3), with three exceptions where the values were inverted: Tomato showed no significant difference in KY, but showed an indication of *lower* K in intercropping; Pea showed significantly *higher* AG biomass (P=0.002) and yield (P=0.012) in intercropping compared to monocropping; and carrot showed no difference in NY, but with an indication of *higher* N in intercropping. A last exception was carrot PY, where there was no significant difference between cropping treatments, but the pattern followed being lower for intercropping compared to monocropping.

For all three species, AG biomass, yield, and harvest index mostly followed the pattern of being significantly the highest on potting soil, followed by sand, and lastly by Mars regolith (P<0.001). Where the difference was not significant (P>0.05), the values always followed the same pattern, except in the harvest indexes of tomato intercrop and monocrop, pea intercrop and carrot monocrop, where sand values were slightly higher than in potting soil (Fig 3A-C). The only significant exception to this pattern were carrot AG biomass and yield, where both were lower in the potting soil intercropping treatment compared to the sand monocropping treatment (P<0.001, Fig 4 and 5). A noteworthy change was seen in pea AG biomass and yield, where both went from being significantly lower in sand compared to potting soil in monocropping, to having no significant difference between the two soils in intercropping (AG biomass: P=0.030; yield: P=0.015) (Fig 3A-B).

For NPK content in yield, the pattern seen above was inverted, where Mars regolith was mostly found to give the highest yield NPK values, and potting soil the lowest values, for both cropping treatments. Mars regolith had significantly higher values than both sand and potting soil for tomato NY and KY (P<0.001), pea P (P=0.007) and K (P<0.001), and carrot N (P<0.011). No significant difference was found amongst any other treatments. The only exception to this pattern was tomato PY, where it was significantly higher in Potting soil compared to Mars regolith monocrop, and higher in sand monocrop compared to Mars regolith monocrop (P=0.041, Fig 3D-F).

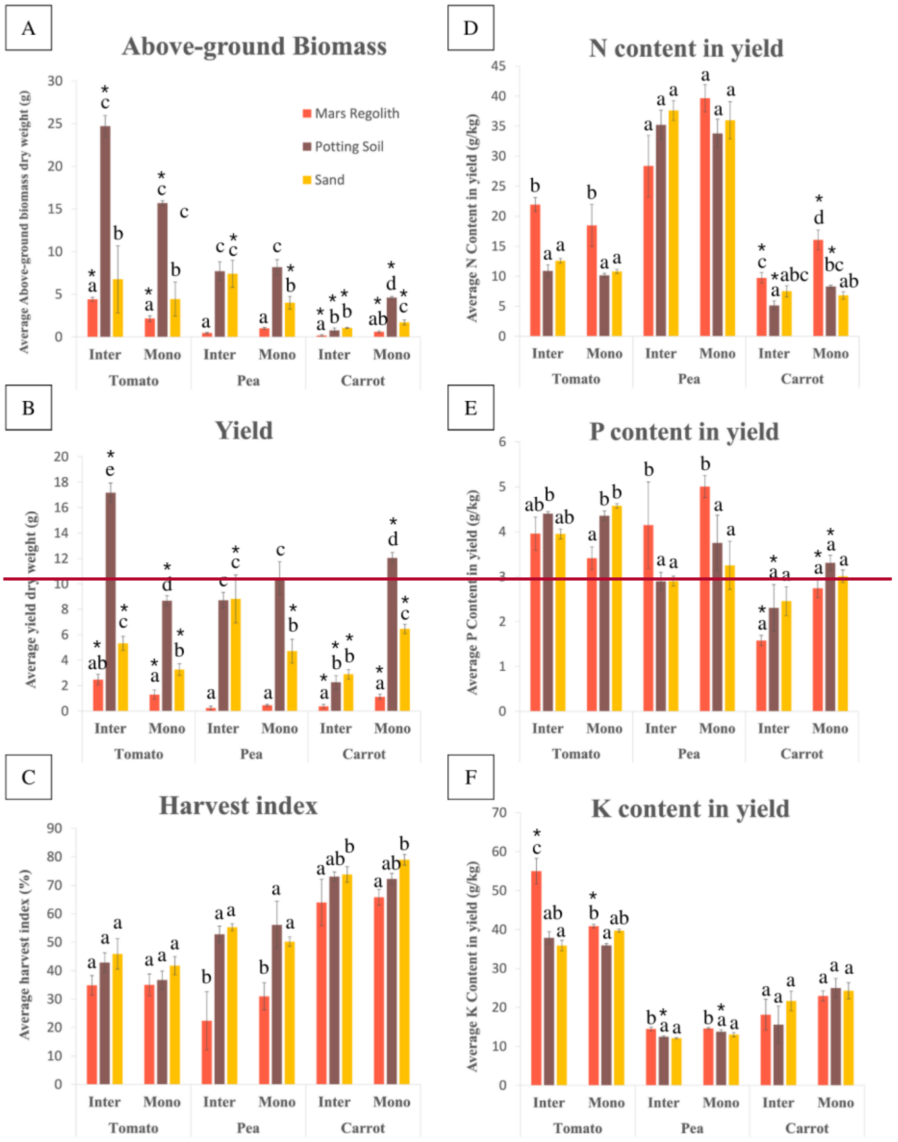

Formatted: English (United States)

Formatted: English (United States)

Fig 3. Comparison between intercropping and monocropping for tomato, pea and carrot in all three soils (Mars regolith simulant, potting soil and sand). A: Above-ground biomass. B: Yield. C: Harvest index. D: N content in yield (NY). E: P content in yield (PY). F: K content in yield (KY). "Inter" = Intercropping treatment, "Mono" = Monocropping treatment. Letters indicate significant differences

between soils within the same cropping treatment and within the same species, at the 0.05 level (Fisher's LSD test). Asterisks (*) indicate significant differences between intercropping and monocropping treatments within the same soil and within the same species, at the 0.05 level (Analysis of Deviance). Error bars represent standard error (SE).

Formatted: English (United States)

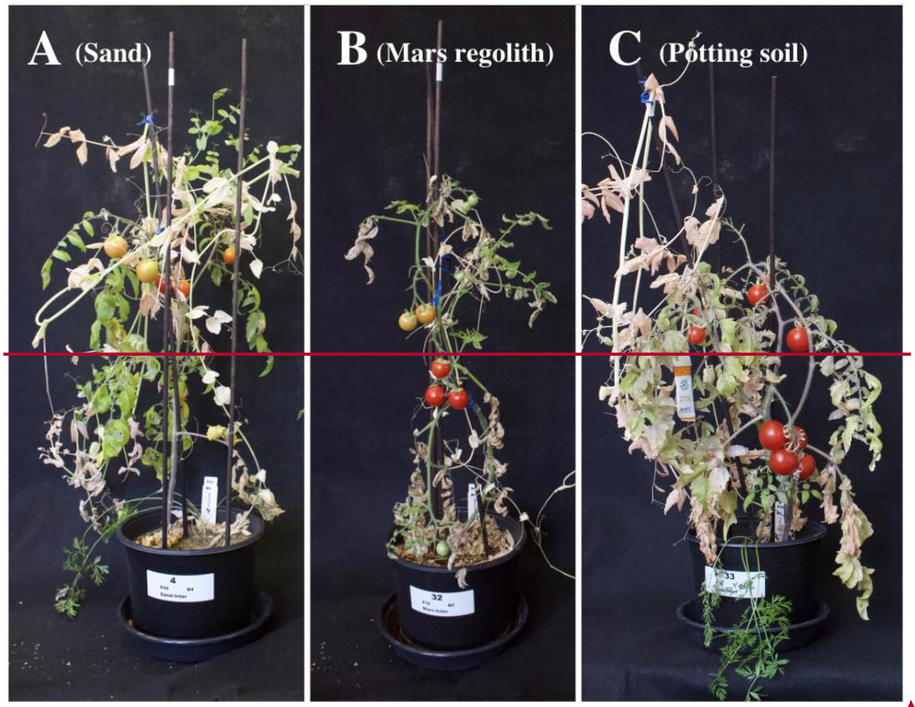

Formatted: English (United States)

Fig 4: Comparison between intercropping treatment from the three soils. A: Sand. B: Mars regolith simulant. C: Potting soil.

Formatted: English (United States)

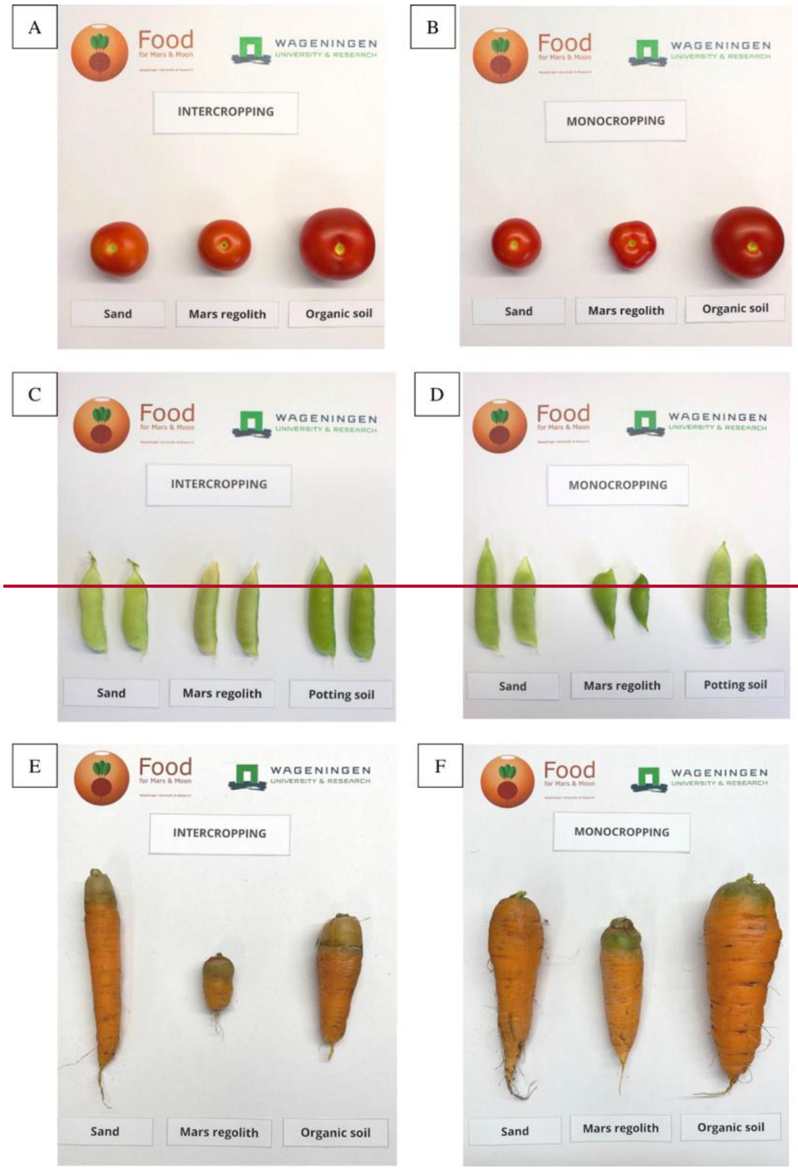

Formatted: English (United States)

Fig 5. Yield comparison between cropping treatments and between soils. Labels in pictures indicating "Organic soil" refer to the potting soil treatment. A: Intercropped tomato. B: Monocropped tomato. C: Intercropped peas. D: Monocropped peas. E: Intercropped carrots. F: Intercropped carrots.

Sand was the only soil to present an overall yield advantage in the intercropping system compared to monocropping (RYT = 1,32, Table 3 and Fig 6), where both tomato and pea showed yield advantage in intercropping over monocropping (tomato relative yield (RY) = 0.54; pea RY = 0,62), and carrot showed yield disadvantage (RY = 0,15). Potting soil showed no overall yield advantage (RYT = 1,00), where tomato over-yielded (RY = 0,66) but both pea and carrot under-yielded (pea RY = 0,28; carrot RY = 0,06). Mars regolith showed under-yielding in the intercropping system compared to monocropping (RYT = 0,93), where tomato over-yielded (RY = 0,64) but both pea and carrot under-yielded (pea RY = 0,18; carrot RY = 0,11).

Table 3. Relative yields for tomato, pea and carrot in each soil treatment, and the total relative yield (RYT) for the whole intercropping system when compared to monocropping, for each of the three soils (MMS-1 Mars regolith simulant, sand and potting soil).

Formatted Table

**Soil**	**Relative yield**			**RYT**
	**Tomato**	**Pea**	**Carrot**	
**Mars regolith**	0,64	0,18	0,11	0,93
**Sand**	0,54	0,62	0,15	1,32
**Potting soil**	0,66	0,28	0,06	1,00

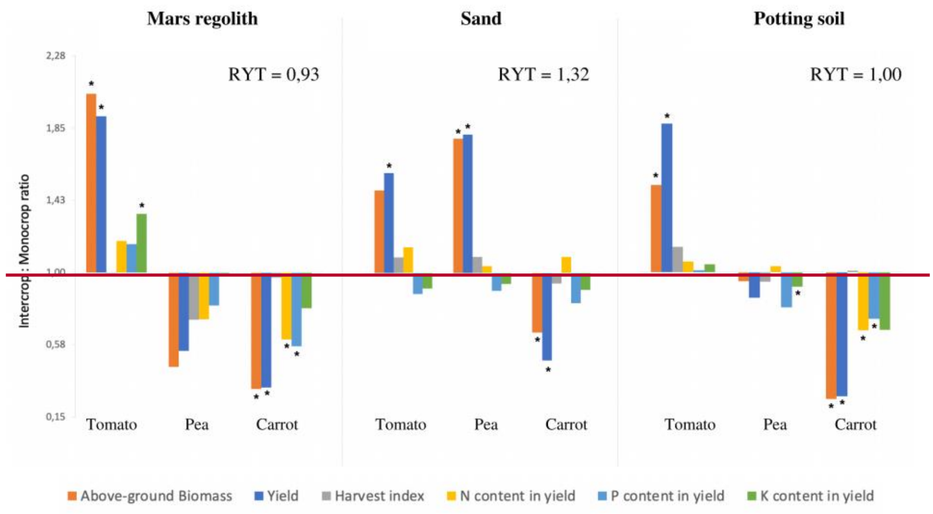

Fig 6. Comparison of intercropping to monocropping ratios and overall system performance for the three species in each of the three soils (Mars regolith simulant, sand and potting soil). Ratios above 1 indicate the variable had a higher value in intercropping relative to monocropping, ratios below 1 indicate the variable had a lower value in intercropping relative to monocropping. RYT (total relative yield) refers to overall yield advantage or disadvantage in the intercropping system. If RYT > 1, this indicates yield advantage from intercropping over monocropping, RYT < 1 indicates yield disadvantage from intercropping over monocropping, and RYT = 1 indicates no difference in yield between the two cropping systems. Variables indicated are above-ground biomass dry weight (g), yield dry weight (g), harvest index (%), and N, P and K content in yield (g/kg)) for each of the three species (tomato, pea and carrot). Asterisks (*) indicate significant differences between intercropping and monocropping values within the same variable, at the 0.05 level.

Presence of Rhizobia bacteria

Visual analysis of roots of peas in both monocropping and intercropping treatments revealed presence of Rhizobia infected nodules in sand and potting soil, but not in Mars regolith (except for one replica from a monocropping treatment which had 3 nodules). Between 17 and 150 nodules were found across the other treatments. There was no significant difference in the number of nodules between potting soil and

sand (Fisher's post hoc test P<0.05), although there was on average more nodules found in sand than in potting soil (Fig 7). Both sand and potting soil showed a significantly higher number of root nodules than Mars regolith (P<0.001). There was also on average more nodules in intercropping compared to monocropping for both soils, although there was considerable variation between replicas, and thus this difference was not significant (P=0.100). Dissection of several nodules per replica showed a pink coloration inside, indicating that the nodules were active (Fig 8).

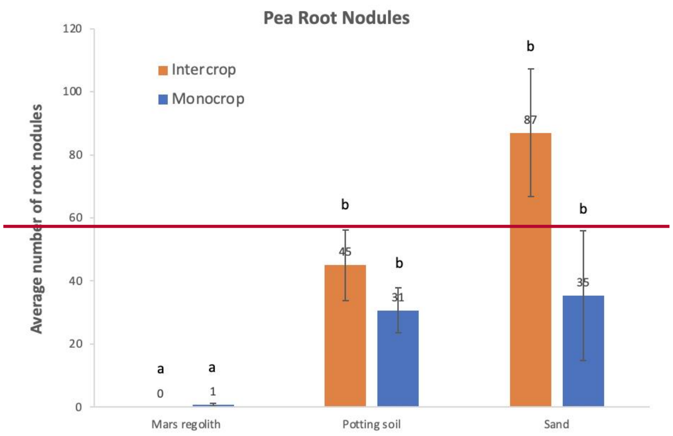

Formatted: English (United States)

Fig 7. Average number of root nodules on pea roots (N=5) per plant. Values are indicated for the monocropping and intercropping treatments from each of the three soils (Mars regolith, potting soil and sand). Data labels indicate the average value. Letters indicate significant differences between cropping treatment and between soils, at the 0.05 level (Fisher's LSD test). Error bars represent standard error (SE)

Formatted: English (United States)

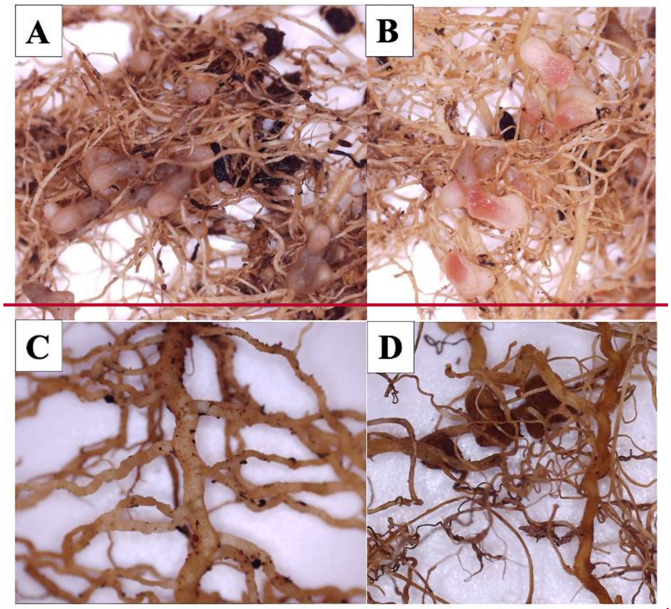

Fig 8. Root and nodule comparison between soils. A: Pea roots from Potting soil monocropping treatment, with intact nodules. B: Pea roots from sand monocropping treatment, with nodules dissected, where a pink coloration can be seen, indicating the nodules were active in N-fixing. C: Pea roots from Mars regolith simulant (intercropping treatment), showing thicker and more tortuous roots. D: Pea roots from Mars regolith (monocropping treatment), from the only replica that showed the presence of nodules. These roots also appear thicker and more tortuous.

Formatted: English (United States)

Discussion

Intercropping effects on RYT for Mars regolith

In Mars regolith, tomato was the only species in the intercropping treatment to show significantly higher performance in both above-ground biomass (AGB) and yield, compared to monocropping. Carrot showed significantly lower AGB and yield in intercropping compared to monocropping, and pea showed no significant difference between the two cropping treatments.

Overall, the intercropping system in Mars regolith showed yield disadvantage over monocropping, with an RYT (total relative yield) value of 0.93. Intercropping advantages or disadvantages can indicate the amount of interspecific competition or facilitation that is occurring within an intercropping system (54)(Fetene, 2003). An RYT of less than 1 means that interspecific competition in intercropping was higher than intraspecific competition in monocropping. This is very likely due to the fact that pea did not form root nodules in symbiosis with the Rhizobia in Mars regolith, thus negating its role as N-fixers and the key advantage of having a legume in an intercropping system. Many studies have demonstrated that when intercropped, pea can compensate for increased nutrient competition, as well as for N deficiency in the soil, by releasing root exudates (i.e. flavonoids) that can promote root nodule formation (47,55) (Leoni et al., 2021; Li et al., 2009). This in turn increases fixation of atmospheric N and helps pea both escape the competitive pressure, as well as relieve the pressure for the other species by leaving more N available in the soil. Without the formation of nodules this was not possible, and instead of relieving the competitive pressure, pea became an added competitive load to the intercropping system in Mars regolith.

The contrast in performance between tomato and the other two species can be attributed to the fact that different crops often show different levels of competition strength when intercropped together. Tomato is a dominant species within intercropping systems, and has been shown to have yield advantages when intercropped with legumes (56). (Olowolaju & Okunlola, 2017). In another study by Wu et al. ((36)2016), where tomatoes were intercropped with potato onion, intercropping promoted the growth of tomato, whilst it inhibited the growth of potato onion.

Tomato is also generally known to be "heavy feeders" with a high demand for nutrient supply (57), (Traoré et al., 2022), which could mean that they monopolisze the resource uptake in the pot. This

Formatted: Font color: Black, English (United States)

Formatted: Font color: Black, English (United States)

Formatted: Font color: Black, English (United States)

Formatted: English (United States)

Formatted: Font color: Black, English (United States)

Formatted: English (United States)

Formatted: English (United States)

Formatted: Font color: Black, English (United States)

higher competitive strength of tomato could explain why it had increased yield performance in intercropping compared to monocropping, since it was put at an advantage compared to pea and carrot, whilst losing this advantage when grown with other tomato plants. It could also explain why in Mars regolith pea and carrot had lower performance in the intercropping treatment, as a higher resource uptake would confer the tomato more nutrients for growth, whilst leaving less resources available to the other two species. Carrot, which depends solely on the nutrients available in the soil, would naturally be at a disadvantage when grown with tomato.

Interestingly, in sand where we saw an adequate number of nodules in pea roots ("adequate" meaning an average of 30 or more nodules per plant (58)), (Farquharson et al., 2022)), there *was* an overall yield advantage in the intercropping system (RYT = 1.32), where although intercropped carrots underperformed, both pea and tomato showed significantly higher yield compared to monocropping. This higher RYT owes to the fact that pea performed particularly well when intercropped in sand, giving equal or higher values in all variables compared to monocropping.

The success of peas in sand is most likely due to the higher number of root nodules that were formed, particularly in the intercropping treatment, allowing for more atmospheric N-fixing. The higher number of root nodules in the sand treatment compared to potting soil can probably be explained by the lower N content available in sand, a factor that has been shown to promote nodulation (55) (Li et al., 2009). Lower N availability has also been linked to an increase in RYT before: in a pea-barley intercrop study, RYT was highest for treatments with no fertilizer compared to treatments with applied fertilizer (59) (Sahota & Malhi, 2012). The high resource demand from the aggressive tomato in the sand intercropping treatment probably caused further decrease in N availability in the soil, which could explain the higher nodulation seen in this treatment (Fig 7).

Potential factors affecting yield performance on Mars regolith

There are a few reasons why nodulation in pea may have been hindered in both Mars treatments. Many studies have shown nodulation and *R. leguminosarum* survival to greatly decrease when under a variety of biotic and abiotic stresses, including high salinity (60) (Zahran, 1999) and soil sterility (61) (Kasper et al., 2019), both of which are features of the MMS-1 Mars regolith used in this study. A potentially aggravating condition was that the regolith was very compact in comparison to both potting soil and

Formatted: Font color: Black, English (United States)

Formatted: Font color: Black, English (United States)

Formatted: Font color: Black, English (United States)

Formatted: Font color: Black, English (United States)

Formatted: Font color: Black, English (United States)

sand. Although it was of "unsorted grade", it was composed mostly of very fine particles that gave it a clay-like texture when wet, and it did not drain well. The roots of the peas in Mars regolith reflected this in that they were smaller, thicker and more tortuous than the roots of peas in sand (and also in potting soil) (Fig 8), which is a common response to denser and coarser soils (62) (Correa et al., 2019). Since soil texture is known to affect salinity, where clay and moist soils tend to be more saline (63) (Zaman et al., 2018), this could have added a salt stress factor to the Mars regolith, negatively affecting rhizobia bacteria. Moreover, the Mars regolith was low in organic matter. Sterilisized soils have been shown to severely hamper formation of nodules, when compared to unsterilisized soils containing more varied microbial communities (61) (Kasper et al., 2019). The chemical composition of the Martian regolith has also been suggested to affect nodulation. A study involving legume-Rhizobia symbiosis in Mars regolith simulants showed more nodulation in the MMS-2 regolith simulant compared to MMS-1, where MMS-2 contains added compounds such as Iron Oxide, Magnesium Oxide, Sulfates and Silicates (64). Another similar study on legume-Rhizobia interactions in MMS-1 suggested that the high pH of the regolith may impair Fe uptake by the plants, Fe being a key factor for plants to sustain a healthy relationship with rhizobia, which would in turn negatively impact nodulation (65).

Formatted: Font color: Black, English (United States)

Formatted: Font color: Black, English (United States)

Formatted: Font color: Black, English (United States)

Formatted: Font color: Black, English (United States)

Formatted: Font color: Black, English (United States)

Another problem with The compactness factor of the regolith could have also posed a problem for plants themselves, regardless of how it may or may not have affected Rhizobia nodulation. Compact soilscompact soils is that it allows for lower gas diffusion and water conductivity, which can lead to anaerobic conditions in the soil and significantly reduce N availability and nutrient uptake (62) (Correa et al., 2019), with more compact soils being linked to lower yields (66). (Johansen et al., 2015). The organic matter present in Earth soils greatly contributes to soil quality, both in reducing soil compactness, as well as being rich in nutrients and the micro-organisms that play a part in making nutrients available for plant uptake (67). (Kong et al., 2011). This could also explain why the absolute values for yield were the highest in potting soil, and why sand also showed higher yield values compared to Mars (although sand is also poor in organic matter, it is not as sterile as the Mars regolith, and may have harboured a wider microbiome that aided in plant performance). The sterile nature of Mars regolith means that, although it has the *chemical elements* necessary to meet the requirements of plant growth (Table 1), these elements are could be mostly absent in the bio-available forms that are necessary for their uptake (68), (Duri et al., 2022), which are often made available by the activity of micro-organisms in the soil.

Formatted: Font color: Black, English (United States)

Formatted: Font color: Black, English (United States)

Formatted: Font color: Black, English (United States)

Formatted: Font color: Black, English (United States)

Nutrient analysis

Potassium content in yield (KY) for Mars regolith intercropped tomato was significantly higher than KY for both sand and potting soil tomato, from both cropping treatments. This could be explained by the fact that optimum K uptake occurs only above 6.5 pH, and is increased further with increased soil moisture (69), (Kuchenbuch et al., 1986), giving Mars regolith an advantageous condition for K uptake especially when compared to potting soil.

PY in Mars monocropped tomato was significantly lower than in Mars intercropped tomato. This could be explained by the fact that P uptake is severely compromised between pH 8 and pH 9, which is exactly where the Mars regolith falls within. However, it is unclear why this was also not the case for carrot and pea PY from Mars regolith, or for the Mars intercropped tomato treatment.

NY for both tomato and carrot were significantly higher in Mars regolith compared to potting soil, even though potting soil had vastly more nitrogen available (table 1). Although higher N availability in soil has been linked to higher yields (70) (Walley et al., 2002), studies have also shown that this higher availability has either no correlation or a negative correlation (71) (Davis et al., 2004) with N content in yield. This could explain why in potting soil we see the highest values for yield but also the lowest values for N content in yield, and vice-versa for Mars regolith.

Mars Regolith limitations and proposed soil ameliorations

Physical and chemical properties of the Mars regolith, such as soil compactness and high pH, may have made it a hostile environment for survival and nodulation of rhizobia bacteria, as well as causing reduced nutrient availability and bioavailability in the soil for ideal plant development, impeding the plants from taking full advantage of their complementarity properties in the intercropping system. For instance, when nodulation occurred and peas were optimiszed for N-fixing, such as in the sand treatments, the intercropping system as it was designed in this experiment gave significant overall yield advantages, indicating the potential for such a method to be used to optimisze crop growth on Mars.

Therefore, a chief solution for the improvement of the system would be to work on the sustainable amelioration of the Mars regolith, taking into account a realistic starting scenario on future Martian

colonies. For example, initially ~~simply~~ selecting a higher grain grade of the regolith on Mars, at the onset of the Martian agricultural system, ~~collected from the Martian surface~~ could potentially already aid in reducing soil compactness, improving drainage, salinity conditions, gas diffusion and nutrient availability.

Following the first crop harvest, using inedible parts to mix in as compost to the regolith could also greatly improve soil conditions. The increased organic matter would not only improve conditions for the survival and nodulation of Rhizobia bacteria, but also support the growth of all other species present by improving the regolith's nutrient content, bioavailability and uptake. Adding compost to MMS-1 Mars regolith results in higher plant biomass, with the highest results at 30:70 simulant:compost mixture (43) ~~(Caporale et al., 2020)~~. Other combinations or varying ratios of candidate species can also be tested in order to find the optimum arrangement for the most efficient species complementarity. For example, a study by El-Gaid et al. (72) ~~(2014)~~ on intercropping tomato and bean found that the highest RYT values were achieved in a combination of 1 tomato plant : 3 bean plants (RYT = 1.26).

Of course, the absence of nodulation and reduced plant biomass and yield on the Mars regolith can be attributed not only to its physical properties and lack of micro-organisms and nutrients, but also to its intrinsic chemical composition. F~~However, f~~urther research would need to be conducted to isolate and identify which and how each of the chemical factors of MMS-1 would influence both in the nodulation of rhizobia as well as in the performance of plants.

Finally, actual Mars regolith and the initial conditions of a Martian colony would pose some additional challenges which, due to the emerging nature of this field of study, were purposefully "bypassed" here in order to reduce variables and produce more reliable results with respect to the focal study system. For example, we manually added a minimal amount of nutrients required for plant growth, disregarding the inherent sterility of the *in situ* regolith and presuming that such nutrients could be supplied from Earth or acquired through an established bioregenerative system on Mars. Furthermore, our simulant did not contain any perchlorates, a compound known to be present on the surface of Mars, nor did we analyse the effects of the regolith's heavy metals on plant growth. Although perchlorate and heavy metal presence can be remedied with the addition of plants or microbes to the soil (21,23), further research could include toxicity remediation and study the holistic effects that such measures would have on the microbial community and overall conditions for plant growth in the Martian regolith. As we build upon our collective knowledge for Martian agriculture, future studies can incorporate findings to produce a more

Formatted: Font color: Black, English (United States)

Formatted: English (United States)

Formatted: Font color: Black, English (United States)

Formatted: English (United States)

Formatted: Font color: Black, English (United States)

overarching understanding of the complete scenario on Mars, including all its challenges and adopted solutions.

Conclusion

In this study we sought to simulate an agricultural scenario likely to be encountered by early Martian colonists, such as the use of small pots, a controlled greenhouse environment, and a nutrient poor Mars regolith simulant with only essential nutrients added. Under such circumstances, we found that an intercropping system can be successful in optimising resource use efficiency if soil conditions are favourable to plant growth and nodulation of N-fixing bacteria, as we saw in the sand treatment (RYT = 1.32). On Mars regolith simulant, intercropping had an overall yield disadvantage compared to monocropping (RYT = 0.93). We postulated that this was most likely due to the absence of rhizobia nodulation in the Mars regolith, which negated the role of pea as a Nitrogen-fixer, impeding crops in the intercropping system from taking full advantage of their complementary properties. We identified that some of the physical and chemical properties of the Mars regolith simulant, such as elevated compactness and high pH, may have created a hostile environment for the survival and nodulation of rhizobia bacteria, while also limiting nutrient availability and bioavailability in the soil necessary for normal crop development. As further research, we suggest considering our proposed soil ameliorations to the Mars regolith, such as utilizing a higher grain grade to reduce soil compactness, and simulate a cyclic system in order to use past harvest waste as compost to increase soil pH and nutrient availability. We acknowledge that certain challenges with the actual regolith and starting conditions on Mars, such as the presence of perchlorates and the initial absolute sterility of the regolith, have been purposefully bypassed in order to maintain the focal objective of the study. Although solutions to such challenges exist, future studies should aim to ultimately integrate all these factors into a single system. As we build towards a more complete and comprehensive knowledge of Martian agriculture, focusing on improving regolith conditions could be key to unlocking the potential of rhizobia-legume interactions and species complementarity, and advance intercropping as a leading method to optimise fresh food production in future Martian colonies.

~~This research showed that intercropping species in small pots, as would be the most likely initial set up in Martian colonies, can work well if optimized for nodulation of N-fixing bacteria, as we saw on the sand treatment where RYT > 1.32. We identified that some of the particular physical and chemical properties~~

of the Mars regolith may have made it a hostile environment for survival and nodulation of rhizobia bacteria, as well as providing reduced nutrient availability and bioavailability in the soil for all species, which would have impeded the plants from taking full advantage of their complementarity properties in the intercropping system. As further research, we suggest considering our proposed ameliorations to the soil properties of the Mars regolith, and reconducting the experiment with the same or a variation of species. Focusing on improving the regolith conditions could be the key to unlock the potential of species complementarity in such a set up, and prove intercropping to be a promising system for optimizing fresh food production in future colonies on Mars.

Formatted: Line spacing: Double

Acknowledgements

The authors wish to thank Zoëe Berkers for help with measurements and harvest, and Paul Goedhart for his advice on the statistics.

Formatted: Line spacing: Double

References

1. Slakey F, Spudis PD. Robots vs. Humans: Who Should Explore Space? Scientific American sp [Internet]. 2008 Feb [cited 2022 Apr 28];18(1):26–33. Available from: https://www.scientificamerican.com/article/robots-vs-humans-who-should-explore/

2. Graham T, Bamsey M. Editor's note for the topical issue "Agriculture in Space." Open Agric. 2016;1(1):68.

3. Guine RPF, Correia PMR, Correia AC, Goncalves F, Brito MFS, Ribeiro JRP. Effect of Drying Temperature on the Physical-chemical and Sensorial Properties of Eggplant (Solanum melongena L.). Curr Nutr Food Sci. 2017;14(1):28–39.

4. Mohammadi X, Deng Y, Matinfar G, Singh A, Mandal R, Pratap-Singh A. Impact of Three Different Dehydration Methods on Nutritional Values and Sensory Quality of Dried Broccoli, Oranges, and Carrots. Foods [Internet]. 2020 [cited 2022 May 19];9(10):1464. Available from: www.mdpi.com/journal/foods

5. Santos PHS, Silva MA. Retention of Vitamin C in Drying Processes of Fruits and Vegetables—A Review. https://doi-org.ezproxy.library.wur.nl/101080/07373930802458911 [Internet]. 2010 [cited 2022 May 19];26(12):1421–37. Available from: https://www-tandfonline-com.ezproxy.library.wur.nl/doi/abs/10.1080/07373930802458911

6. Pressman P, Clemens RA, Hayes AW. Bioavailability of micronutrients obtained from supplements and food. Toxicology Research and Application. 2017;1:239784731769636.

7. Kanas N, Sandal G, Boyd JE, Gushin VI, Manzey D, North R, et al. Psychology and culture during long-duration space missions. Acta Astronaut [Internet]. 2009 [cited 2022 May 19];64:659–77. Available from: www.elsevier.com/locate/actaastro

8. Morphew ME. Psychological and human factors in long duration spaceflight. McGill Journal of Medicine. 2001;6(1):74–80.

9. Aistis LE; A;, Sachs NA;, Williams M;, Roberts JD, Goldstein R, Gerdes ME, et al. Reducing Anxiety with Nature and Gardening (RANG): Evaluating the Impacts of Gardening and Outdoor Activities on Anxiety among U.S. Adults during the COVID-19 Pandemic. Int J Environ Res Public Health [Internet]. 2022;19:5121. Available from: https://doi.org/10.3390/ijerph19095121

10. Clatworthy J, Hinds J, Camic PM. Gardening as a mental health intervention: A review. Mental Health Review Journal. 2013;18(4):214–25.

11. Nelson M, Dempster WF, Allen JP. Integration of lessons from recent research for "Earth to Mars" life support systems. Advances in Space Research. 2008;41(5):675–83.

12. Maggi F, Pallud C. Space agriculture in micro- and hypo-gravity: A comparative study of soil hydraulics and biogeochemistry in a cropping unit on Earth, Mars, the Moon and the space station. Planet Space Sci. 2010 Dec 1;58(14–15):1996–2007.

13. Silverstone S, Nelson M, Alling A, Allen J. Development and research program for a soil-based bioregenerative agriculture system to feed a four person crew at a Mars base. Advances in Space Research. 2003;31(1):69–75.

14. ESA. Comparing the atmospheres of Mars and Earth [Internet]. 2018 [cited 2022 Aug 20]. Available from: https://www.esa.int/ESA_Multimedia/Images/2018/04/Comparing_the_atmospheres_of_Mars_and_Earth

Formatted: English (United States)

15. Orosei R, Ding C, Fa W, Giannopoulos A, Hérique A, Kofman W, et al. The Global Search for Liquid Water on Mars from Orbit: Current and Future Perspectives. Life [Internet]. 2020 [cited 2022 Aug 20];10:120. Available from: www.mdpi.com/journal/life

16. Tack N, Wamelink GWW, Denkova AG, Schouwenburg M, Hilhorst H, Wolterbeek HT, et al. Influence of Martian Radiation-like Conditions on the Growth of Secale cereale and Lepidium sativum. Frontiers in Astronomy and Space Sciences. 2021;8(August):1–9.

17. NASA. NASA Science: Mars Exploration Program. 2022 [cited 2022 Aug 20]. Mars facts. Available from: https://mars.nasa.gov/all-about-mars/facts/

18. Liu J, Li H, Sun L, Guo Z, Harvey J, Tang Q, et al. In-situ resources for infrastructure construction on Mars: A review. International Journal of Transportation Science and Technology [Internet]. 2022;11(1):1–16. Available from: https://doi.org/10.1016/j.ijtst.2021.02.001

19. Mariani JF. Veggie Fact Sheet. The Encyclopedia of American Food and Drink. 2020;520–520.

20. Clark BC, Kounaves SP. Evidence for the distribution of perchlorates on Mars. Int J Astrobiol. 2015;15(4):311–8.

21. González Henao S, Ghneim-Herrera T. Heavy Metals in Soils and the Remediation Potential of Bacteria Associated With the Plant Microbiome. Front Environ Sci. 2021;9(April):1–17.

22. Koehle AP, Brumwell SL, Seto EP, Lynch AM, Urbaniak C. Microbial applications for sustainable space exploration beyond low Earth orbit. NPJ Microgravity. 2023;9(1).

23. Misra G, Smith W, Garner M, Loureiro R. Potential Biological Remediation Strategies for Removing Perchlorate from Martian Regolith. New Space. 2021;9(4).

24. Wilson EH, Atreya SK, Kaiser RI, Mahaffy PR. Perchlorate formation on Mars through surface radiolysis-initiated atmospheric chemistry: A potential mechanism. J Geophys Res Planets [Internet]. 2016;121:1472–87. Available from: doi:10.1002/2016JE005078

25. Xu D, Yu X, Chen J, Li X, Chen J, Li JH. Effects of compost as a soil amendment on bacterial community diversity in saline–alkali soil. Front Microbiol. 2023;14(September):1–11.

26. Wamelink GWW, Schug L, Frissel JY, Lubbers I. Growth of Rucola on Mars soil simulant under the influence of pig slurry and earthworms. Open Agric. 2022;7(1):238–48.

27. Ramírez DA, Kreuze J, Amoros W, Valdivia-Silva JE, Ranck J, Garcia S, et al. Extreme salinity as a challenge to grow potatoes under Mars-like soil conditions: targeting promising genotypes. Int J Astrobiol. 2017;18(1):1–7.

28. Wamelink GWW, Frissel JY, Krijnen WHJ, Verwoert MR, Goedhart PW. Can plants grow on mars and the moon: A growth experiment on mars and moon soil simulants. PLoS One. 2014;9(8).

29. Wamelink GWW, Frissel JY, Krijnen WHJ, Verwoert MR. Crop growth and viability of seeds on Mars and Moon soil simulants. Open Agric. 2019;4(1):509–16.

30. Mt.Pleasant J. Food yields and nutrient analyses of the three sisters: A Haudenosaunee cropping system. Ethnobiology Letters. 2016;7(1):87–98.

31. Jensen ES, Carlsson G, Hauggaard-Nielsen H. Intercropping of grain legumes and cereals improves the use of soil N resources and reduces the requirement for synthetic fertilizer N: A global-scale analysis. Agron Sustain Dev. 2020;40(1).

32. Latati M, Bargaz A, Belarbi B, Lazali M, Benlahrech S, Tellah S, et al. The intercropping common bean with maize improves the rhizobial efficiency, resource use and grain yield under low phosphorus availability. European Journal of Agronomy [Internet]. 2016;72:80–90. Available from: http://dx.doi.org/10.1016/j.eja.2015.09.015

Formatted: English (United States)

Formatted: English (United States)

Formatted: English (United States)

Formatted: English (United States)

33. Li X, Mu Y, Cheng Y, Liu X, Nian H. Effects of intercropping sugarcane and soybean on growth, rhizosphere soil microbes, nitrogen and phosphorus availability. Acta Physiol Plant. 2013;35(4):1113–9.

34. Nasar J, Shao Z, Arshad A, Jones FG, Liu S, Li C, et al. The effect of maize–alfalfa intercropping on the physiological characteristics, nitrogen uptake and yield of maize. Plant Biol. 2020 Nov 1;22(6):1140–9.

35. Patra DD, Subbiah B V. 15N studies on the transfer of legume-fixed nitrogen to associated cereals in intercropping systems. Biol Fertil Soils. 1986;2:165–71.

36. Wu X, Wu F, Zhou X, Fu X, Tao Y, Xu W, et al. Effects of intercropping with potato onion on the growth of tomato and rhizosphere alkaline phosphatase genes diversity. Front Plant Sci. 2016;7(JUNE2016):1–13.

37. Hauggaard-Nielsen H, Ambus P, Jensen ES. Interspecific competition, N use and interference with weeds in pea-barley intercropping. Field Crops Res. 2001;70(2):101–9.

38. Bybee-Finley KA, Ryan MR. Advancing intercropping research and practices in industrialized agricultural landscapes. Agriculture (Switzerland). 2018;8(6).

39. Stern WR. Nitrogen fixation and transfer in intercrop systems. Field Crops Res. 1993;34(3–4):335–56.

40. Gatsios A, Ntatsi G, Celi L, Said-Pullicino D, Tampakaki A, Giannakou I, et al. Nitrogen nutrition optimization in organic greenhouse tomato through the use of legume plants as green manure or intercrops. Agronomy. 2019;9(11).

41. Salgado GC, Ambrosano EJ, Rossi F, Otsuk IP, Ambrosano GMB, Santana CA, et al. Biological n fixation and n transfer in an intercropping system between legumes and organic cherry tomatoes in succession to green corn. Agriculture (Switzerland). 2021;11(8).

42. Peters GH, Abbey W, Bearman GH, Mungas GS, Smith JA, Anderson RC, et al. Mojave Mars simulant-Characterization of a new geologic Mars analog. Icarus. 2008;197(2):470–9.

43. Caporale AG, Vingiani S, Palladino M, El-Nakhel C, Duri LG, Pannico A, et al. Geo-mineralogical characterisation of Mars simulant MMS-1 and appraisal of substrate physico-chemical properties and crop performance obtained with variable green compost amendment rates. Science of the Total Environment [Internet]. 2020;720:137543. Available from: https://doi.org/10.1016/j.scitotenv.2020.137543

44. Anderson MS, Ewert MK, Keener JF, Wagner SA. Life Support Baseline Values and Assumptions Document. Nasa/Tp-2015-218570 [Internet]. 2015;(March):1–220. Available from: http://docs.lib.purdue.edu/cgi/viewcontent.cgi?article=1002&context=nasatr

45. Lipiec J, Medvedev V V., Birkas M, Dumitru E, Lyndina TE, Rousseva S, et al. Effect of soil compaction on root growth and crop yield in Central and Eastern Europe. Int Agrophys. 2003;17(2):61–9.

46. Riotte L. Carrots Love Tomatoes: Secrets of Companion Planting for Successful Gardening. 2nd ed. North Adams, MA: Storey Publishing, LLC; 1998.

47. Leoni F, Hazrati H, Fomsgaard IS, Moonen AC, Kudsk P. Determination of the Effect of Co-cultivation on the Production and Root Exudation of Flavonoids in Four Legume Species Using LC-MS/MS Analysis. J Agric Food Chem. 2021;69(32):9208–19.

48. Ho TTK, Tra VT, Le TH, Nguyen NKQ, Tran CS, Nguyen PT, et al. Compost to improve sustainable soil cultivation and crop productivity. Case Studies in Chemical and Environmental Engineering [Internet]. 2020;1(March):100211. Available from: https://doi.org/10.1016/j.cscee.2022.100211

Formatted: English (United Kingdom)

Formatted: English (United States)

49. Ali MY, Sina AAI, Khandker SS, Neesa L, Tanvir EM, Kabir A, et al. Nutritional composition and bioactive compounds in tomatoes and their impact on human health and disease: A review. Foods. 2021;10(1).

50. Arscott SA, Tanumihardjo SA. Carrots of many colors provide basic nutrition and bioavailable phytochemicals acting as a functional food. Compr Rev Food Sci Food Saf. 2010;9(2):223–39.

51. Kumari T, Deka SC. Potential health benefits of garden pea seeds and pods: A review. Legume Science. 2021;3(2):1–13.

52. Karunakaran R, Ramachandran VK, Seaman JC, East AK, Mouhsine B, Mauchline TH, et al. Transcriptomic analysis of Rhizobium leguminosarum biovar viciae in symbiosis with host plants Pisum sativum and Vicia cracca. J Bacteriol. 2009;191(12):4002–14.

53. Schumpp O, Deakin WJ. How inefficient rhizobia prolong their existence within nodules. Trends Plant Sci [Internet]. 2010;15(4):189–95. Available from: http://dx.doi.org/10.1016/j.tplants.2010.01.001

54. Fetene M. Intra- and inter-specific competition between seedlings of Acacia etbaica and a perennial grass (Hyparrenia hirta). J Arid Environ. 2003;55(3):441–51.

55. Li Y, Sun J, LI C, Li L, Cheng X, Zhang F. Effects of interspecific interactions and nitrogen fertilization rates on the agronomic and nodulation characteristics of intercropped faba bean. Scientia Agricultura Sinica. 2009;42:3467−3474.

56. Olowolaju ED, Okunlola GO. COMPARATIVE ADVANTAGE OF INTERCROPPING MAIZE, COWPEA AND TOMATO TO SOLE AND MIXED CULTURE. Annals of West University of Timişoara, ser Biology. 2017;20(1):31–8.

57. Traoré A, Bandaogo AA, Savadogo OM, Saba F, Ouédraogo AL, Sako Y, et al. Optimizing Tomato (Solanum lycopersicum L.) Growth With Different Combinations of Organo-Mineral Fertilizers. Front Sustain Food Syst. 2022;5(March):1–7.

58. Farquharson E, Ballard R, Herridge D, Ryder M, Denton M, Webste A, et al. INOCULATING LEGUMES : PRACTICE AND SCIENCE. Grains Research and Development Corporation. 2022;

59. Sahota TS, Malhi SS. Intercropping barley with pea for agronomic and economic considerations in northern Ontario. Agricultural Sciences. 2012;03(07):889–95.

60. Zahran HH. Rhizobium -Legume Symbiosis and Nitrogen Fixation under Severe Conditions and in an Arid Climate . Microbiology and Molecular Biology Reviews. 1999;63(4):968–89.

61. Kasper S, Christoffersen B, Soti P, Racelis A. Abiotic and biotic limitations to nodulation by leguminous cover crops in south Texas. Agriculture (Switzerland). 2019;9(10).

62. Correa J, Postma JA, Watt M, Wojciechowski T. Soil compaction and the architectural plasticity of root systems. J Exp Bot. 2019;70(21):6019–34.

63. Zaman M, Shahid SA, Heng L. Guideline for Salinity Assessment, Mitigation and Adaptation Using Nuclear and Related Techniques. Guideline for Salinity Assessment, Mitigation and Adaptation Using Nuclear and Related Techniques. 2018.

64. Rainwater R, Mukherjee A. The legume-rhizobia symbiosis can be supported on Mars soil simulants. PLoS One [Internet]. 2021;16(12 December):1–10. Available from: http://dx.doi.org/10.1371/journal.pone.0259957

65. Harris F, Dobbs J, Atkins D, Ippolito JA, Stewart JE. Soil fertility interactions with Sinorhizobiumlegume symbiosis in a simulated Martian regolith; effects on nitrogen content and plant health. PLoS One [Internet]. 2021;16(9 September):1–13. Available from: http://dx.doi.org/10.1371/journal.pone.0257053

Formatted: English (United States)

66. Johansen TJ, Thomsen MG, Løes AK, Riley H. Root development in potato and carrot crops – influences of soil compaction. Acta Agric Scand B Soil Plant Sci [Internet]. 2015;65(2):182–92. Available from: http://dx.doi.org/10.1080/09064710.2014.977942

67. Kong AYY, Scow KM, Córdova-Kreylos AL, Holmes WE, Six J. Microbial community composition and carbon cycling within soil microenvironments of conventional, low-input, and organic cropping systems. Soil Biol Biochem [Internet]. 2011;43(1):20–30. Available from: https://www.ncbi.nlm.nih.gov/pmc/articles/PMC3624763/pdf/nihms412728.pdf

68. Duri LG, Caporale AG, Rouphael Y, Vingiani S, Palladino M, De Pascale S, et al. The Potential for Lunar and Martian Regolith Simulants to Sustain Plant Growth: A Multidisciplinary Overview. Frontiers in Astronomy and Space Sciences. 2022;8(January):1–16.

69. Kuchenbuch R, Claassen N, Jungk A. Potassium availability in relation to soil moisture - I. Effect of soil moisture on potassium diffusion, root growth and potassium uptake of onion plants. Plant Soil. 1986;95(2):221–31.

70. Walley F, Yates T, van Groenigen JW, van Kessel C. Relationships Between Soil Nitrogen Availability Indices, Yield, and Nitrogen Accumulation of Wheat. Soil Science Society of America Journal. 2002;66(5):1549–61.

71. Davis DR, Epp MD, Riordan HD, Davis DR. Changes in USDA Food Composition Data for 43 Garden Crops, 1950 to 1999. J Am Coll Nutr. 2004;23(6):669–82.

72. El-Gaid MAA, Al-Dokeshy MH, Nassef DMT. Effects of intercropping system of tomato and common bean on growth, yield components and land equivalent ratio in new valley governorate. Asian Journal of Crop Science [Internet]. 2014;6(3):254–61. Available from: 10.3923/AJCS.2014.254.261

73. Achilles CN, Downs RT, Ming DW, Rampe EB, Morris R V., Treiman AH, et al. Mineralogy of an active eolian sediment from the Namib dune, Gale crater, Mars. J Geophys Res Planets. 2017;122(11):2344–61.

74. Hecht MH, Kounaves SP, Quinn RC, West SJ, Young SMM, Ming DW, et al. Detection of Perchlorate and the Soluble Chemistry of Martian Soil at the Phoenix Lander Site. Science (1979). 2009;325(July).

Formatted: English (United States)

Appendix 1

Comparison of the mineralogical, physical and chemical properties between the Mars regolith from the Rocknest eolian deposit in the Gale crater on Mars and the MMS-1 Mars regolith simulant. Values are given in weight percentage (wt%). The table contains compiled data taken from multiple sources and own measurements.

Formatted Table

**Concentration (wt%)**	**Mars regolith (Rocknest bulk)**	**Mars regolith simulant (MMS-1)**
SiO2	43	49.4
TiO2	1.2	1.09
Al2O3	9.4	17.1
Cr2O3	0.5	0.05
Fe2O3	–	10.87
FeO	19.2	–
MnO	0.4	0.17
MgO	8.7	6.08
CaO	7.3	10.45
Na2O	2.7	3.28
K2O	0.5	0.48
P2O5	1	0.17
SO3	5.5	0.1
Cl	0.7	–
Total composition %	100	100
pH	7.7 ± 0.5[1]	7.28 +/− 0.1[2]
Particle Size	<2mm	<1mm
Source	(73)	(42)

[1]The pH value is absent from Gale crater data. The value of 7.7± 0.5 is from data collected at the Mars Phoenix Lander site (74). ~~(Hecht et al., 2009).~~

[2]This pH value was measured directly from the regolith that we used, before the start of the experiment.

Appendix 2.

pH, EC and nutrient contents of Hoagland nutrient solution.

**pH**	**EC**	**NH_4**	**K**	**Na**	**Ca**	**Mg**	**NO3**	**Cl**	**S**	**HCO_3**	**P**	**Si**	**Fe**	**Mn**	**Zn**	**B**	**Cu**	**Mo**
	(mS/cm)	(mmol/l)											(µmol/l)					
6,0	1,9	0,8	6,4	0,6	3,6	1,2	10,2	0,4	1,8	0,9	2,07	0,15	51	13	5,2	28	1,0	0,5

Formatted Table

Peer review for Manuscript PONE-D-23-12961

Dear Dr. Harris,

Thank you for your kind feedback on our submitted manuscript. Below, we have indicated in blue the actions taken to address each of your suggested revisions.

Line numbers mentioned here refer to the line numbers on the document with tracked changes on ("Revised Manuscript with Track Changes").

We hope to have properly addressed your concerns.

Kind regards,
Rebeca Goncalves

Overall, the authors present a novel study with an interesting premise, good study design, and overall suitable metrics to measure their results. The growth of plants in-situ in regolith is a complex and relatively new topic and with limited studies on this subject, these studies will lay groundwork for later experiments. The use of agricultural metrics with intercropping in this paper bring a fresh approach to regolith studies and the use of a plant-rhizobium symbiosis builds on existing literature. The question and build up is mostly there to give context for intercropping in the introduction and the sources cited are relevant articles. However, one concern I have with this sort of research is that it does not address some of the crucial issues that growing things in-situ on Mars would encounter. That does not make this any less useful, but it maybe good to mention some of the other things that would need to be dealt with just to highlight where the focus of this research is. The data provided adequately supports the authors claims, but the discussion could use some elaboration to better understand how it fits the context of the field as a whole. The paper is an important contribution to a growing field and with a few minor revisions I would recommend this paper for publication. I have below outlined proposed revisions and additions to the article.

Major revisions:

None

Minor revisions:

Introduction

Generally speaking, it may be good to address some of the current literature on problems with simulant experiments that have been brought up. Issues like presence of perchlorates and other obstacles that maybe an issue should be briefly mentioned. On this note, addressing the soil properties of regolith a bit more in depth would help readers to understand why it is important to reestablish agricultural knowledge in this medium. Challenges with Martian regolith, including presence of perchlorates, were added in lines 179-215.

Line	Revisions recommended

Peer review for Manuscript PONE-D-23-12961

160-163	A justification for why Rhizobium Leguminosarum (RL) was used maybe helpful. This study does not address the specifics of the symbiosis nor study it in detail. There is nothing wrong with this as the focus of the study was elsewhere, but I would alter the language and explain the choice of using RL as a symbiote over something like Sinorhizobium meliloti which has been tested in multiple studies. Also, the species “meliloti” is spelled incorrectly in the manuscript.

The justification was moved to the methods section (lines 367-371). Indeed it seemed more relevant to omit the mention of the other two species and highlight that RL was chosen due to its nodulation success that we saw in a previous in-house experiment using the same MMS-1 regolith.

Methods

Line	Revisions recommended
257	Hoagland solution misspelled as Hoogland solution

Fixed, it’s now on line 410.

Discussion

One thing to acknowledge in the discussion is the use of Hoagland solution and addition of soil to the regolith and sand. Native Martian regolith (and most simulants) have no nitrogen in them. Adding fertilizer like this inherently alters the fertility of the regolith and thus the focal study system. This maybe needed to support the targets of your study, but it needs to be acknowledged. The bypassing of some of the regolith challenges, including the addition of fertilizer and presence of perchlorates in the regolith, is now acknowledged at the end of the discussion, in lines 806-819.

Line	Revisions recommended
524-543	Finding some more sources about bacteria or bacterial symbiosis in regolith to help support this section would be excellent. It has a good start and I would say it is almost there but I think there are a few key aspects missing such as pH and presence of toxins in regolith.

Other studies involving rhizobia-legume interactions in mars regolith have been mentioned, expanding a little the discussion on reasons why nodulation may have been impaired, which includes the high pH factor of the regolith (lines 713-719).

Conclusions

Line	Revisions recommended
596-598	I think this line needs some qualifying elements to it, as it is it overextends a bit. Under what conditions did this experiment occur? This is not regolith as such, there are issues to overcome. Something along the lines of "with the addition of X nutrients".

The conclusion was rewritten and includes the suggestions mentioned above. Lines 827-849.

Appendix

Line	Revisions recommended
883	Table unreadable in PDF format

Fixed font size.

Additional revisions:

The abstract was slightly reworded to make some aspects of it clearer.

Reference style has been changed to Vancouver according to journal guidelines. You'll see the revisions throughout the text. Also, additional references have been included in the reference list.

Tables font have been formatted to match font of text.

Text was standardised to UK English spelling.
