# Supplementary material for: Intercropping on Mars: A promising system to optimise fresh food production in future martian colonies
Source: PLoS One. 2024 May 1;19(5):e0302149. doi: 10.1371/journal.pone.0302149 (PMC11062560; doi:10.1371/journal.pone.0302149)
Supplement: Attachment — Submitted filename: Reviewer.pdf [file pone.0302149.s006.pdf]

Reviewer:

- **Comments to the Author:**
- The article is interesting and the results look original, but serious deficiencies have been observed:
- **Title** is acceptable.
- **Abstract**:
- 1) Underscore the scientific value-added to your paper in your abstract. Your abstract should clearly state the essence of the problem you are addressing, what you did and what you found and recommend. That will help a prospective reader of the abstract to decide if they wish to read the entire article.
- 2) Please add the values of the traits mentioned in the abstract section.

-Keywords is acceptable

Introduction:

- The introduction need more focus on mechanisms instead of narration.

- Clearly state whether your objective is novel or no.

- What is your research innovation and novelty?

- The introduction need more focus on mechanisms instead of narration.

- Please write the research hypotheses at the end of the introduction.

Material and method:

- In the materials and methods section, please remove irrelevant and redundant descriptions. Some of the mentioned explanations should be moved to the introduction section. For example, delete lines 245 to 249.
- Add references to the material and method of the traits being measured.

Results and discussion:

- Do not re-report the results in the discussion section.

- **Conclusion** is acceptable.

References

- The number of references is too high, please remove some references.
  Kind regards,
  Reviewer

To finish, I think that the work is very interesting and it could be accepted for publication after some corrections and amendments.

- Why did you choose two irrigation levels?

- The distance between two irrigation levels is more and longer.

- In the abstract, you wrote the years of the experiment as 2017 to 2018, but in the materials and methods, you wrote 2017 to 2019. Please correct it.

- In line 123, this fertilizer is Mycoroot brand, which is not in liquid form. Why did you write 10 mL?

- In line 127, Write the complete specifications of liquid fertilizer.

- Sage is a perennial plant. What year did your moisture stress start?

- Insufficient description of experimental design and material and methods generally.

- Add references to the material and method of the traits being measured.

- Write down how to irrigate, fertilize, planting and harvesting dates, control weeds, pests, etc.

- Write down how to apply irrigation stress treatments completely.

--The method of irrigation is not clear.

- Report the amount of water used in each treatment.

- Also, report the water use efficiency.

- The information about the brand, model and country of several analytical equipment are missed in the section "material and methods".

- Please also report the percentage of colonization.

- Please also report the percentage of colonization.

- Please also write the formula for calculating the essential oil content and the essential oil yield.

- The number of traits reported in this paper is very small. Please be sure to report the physiological characteristics.

- The amount of phosphorus in the soil of your study, which is reported in table number one, is too high. Probably misreported. Please correct it.

- Tables 3 and 5 should be given as supplementary tables.

-It is a very weak article and is not recommended for publication at all.

Results and discussion:

- Insufficient presentation (particularly comparison of means) and description of the results. Too many illustrations, which are moreover insufficiently annotated.

- Section of the result and discussion are the most important of paper section. The result and discussion of this paper are too weak and should be rewritten. The authors should be added the main reasons for increasing and decreasing of measured traits.

Conclusion

-Provide recommendations for future studies based on the results obtained in this study.

Kind regards,

Reviewer

To finish, the paper is not recommended for publication for the following reasons:

1- The number of measured and reported attributes is not enough.

2. Qualitative traits have not been measured and reported.

3. The percentage of oil and peanut oil compounds are not reported.

4. The treatments are not chosen correctly. Peanut cannot compete with sorghum, but the treatments have been selected in rows and one by one.

5. The quality level of the article is low.

Kind regards,

Reviewer
