# Supplementary material for: Intercropping on Mars: A promising system to optimise fresh food production in future martian colonies
Source: PLoS One. 2024 May 1;19(5):e0302149. doi: 10.1371/journal.pone.0302149 (PMC11062560; doi:10.1371/journal.pone.0302149)
Supplement: Attachment — Submitted filename: Response to Reviewer 2.pdf [file pone.0302149.s007.pdf]

Dear Dr. Rezaei-Chiyaneh,

Thank you very much for your kind feedback on our submitted manuscript.

We have indicated in the following document the actions taken to address each of your suggested revisions.

These are right next to your highlighted points under “item 6 – Review Comments to the Author”.

Please note that the line numbers refer to the document with *track changes.*

We hope to have properly addressed your concerns.

Kind regards,

Rebeca Gonçalves

Cancel Save and Close

PONE-D-23-12961R1
"Intercropping on Mars: A Promising System to Optimise Fresh Food Production in Future Martian Colonies"
Revision 1

Esmaeil Rezaei-Chiyaneh (Reviewer 2)

**Reviewer Recommendation Term:**	Minor Revision
**Rate Review:**	0
**Custom Review Question(s):**	**Response**
**Comments to the Author**<br><br>1. If the authors have adequately addressed your comments raised in a previous round of review and you feel that this manuscript is now acceptable for publication, you may indicate that here to bypass the "Comments to the Author" section, enter your conflict of interest statement in the "Confidential to Editor" section, and submit your "Accept" recommendation.	All comments have been addressed
2. Is the manuscript technically sound, and do the data support the conclusions?<br><br>The manuscript must describe a technically sound piece of scientific research with data that supports the conclusions. Experiments must have been conducted rigorously, with appropriate controls, replication, and sample sizes. The conclusions must be drawn appropriately based on the data presented.	Yes
3. Has the statistical analysis been performed appropriately and rigorously?	Yes
4. Have the authors made all data underlying the findings in their manuscript fully available?<br><br>The PLOS Data policy requires authors to make all data underlying the findings described in their manuscript fully available without restriction, with rare exception (please refer to the Data Availability Statement in the manuscript PDF file). The data should be provided as part of the manuscript or its supporting information, or deposited to a public repository. For example, in addition to summary statistics, the data points behind means, medians and variance measures should be available. If there are restrictions on publicly sharing data—e.g. participant privacy or use of data from a third party—those must be specified.	Yes

5. Is the manuscript presented in an intelligible fashion and written in standard English?<br><br>PLOS ONE does not copyedit accepted manuscripts, so the language in submitted articles must be clear, correct, and unambiguous. Any typographical or grammatical errors should be corrected at revision, so please note any specific errors here.	Yes
6. Review Comments to the Author<br><br>Please use the space provided to explain your answers to the questions above. You may also include additional comments for the author, including concerns about dual publication, research ethics, or publication ethics. (Please upload your review as an attachment if it exceeds 20,000 characters)	Reviewer:<br>- Comments to the Author:<br>- The article is interesting and the results look original, but serious deficiencies have been observed:<br>- Title is acceptable.<br>- Abstract:<br>-1) Underscore the scientific value-added to your paper in your abstract. Your abstract should clearly state the essence of the problem you are addressing, what you did and what you found and recommend. That will help a prospective reader of the abstract to decide if they wish to read the entire article.<br>-2) Please add the values of the traits mentioned in the abstract section.<br>-Keywords is acceptable<br><br>Introduction:<br>-The introduction need more focus on mechanisms instead of narration.<br>-Clearly state whether your objective is novel or no.<br>-What is your research innovation and novelty?<br>- The introduction need more focus on mechanisms instead of narration.<br>-Please write the research hypotheses at the end of the introduction.<br>Material and method:<br>-In the materials and methods section, please remove irrelevant and redundant descriptions. Some of the mentioned explanations should be moved to the introduction section. For example, delete lines 245 to 249.<br>-Add references to the material and method of the traits being measured.<br><br>Results and discussion:<br>-Do not re-report the results in the discussion section.<br><br>- Conclusion is acceptable.<br>References<br>-The number of references is too high, please remove some references.<br>Kind regards,<br>Reviewer
7. PLOS authors have the option to publish the peer review history of their article (what does this mean?). If published, this will include your full peer review and any attached files.<br><br>If you choose "no", your identity will remain anonymous but your review may still be made public.<br><br>**Do you want your identity to be public for this peer review?** For information about this choice, including consent withdrawal, please see our Privacy Policy.	**Yes:** Esmaeil Rezaei-Chiyaneh

A line has been added to highlight the value the study adds to the challenges of this field of research, and to highlight the novelty of the study

The traits values have been added

Due to the multidisciplinary nature of this study, we indeed felt the need to add a slightly more ample background to both sides of the field, since the targeted audience are people from both the space sector and the biology/agriculture sector. This is hence why the introduction may feel slightly more "narrated". This was also done following the 1st reviewer's suggestions to add more information on certain topics.

A line has been added about the novelty of the study (lines 182 - 184).

The hypotheses have been put forward in lines 201 - 205

All irrelevant or redundant information have been removed. Lines 245-249 have been left as it is the only time the actual complementarity properties of each species are mentioned.

A reference is given for the RYT calculations in line 349.

You're right, the reinstatement of results was removed from the beginning of the discussion. Only a very short statement was left for context before each new discussion point.

Right again, yet unfortunately we couldn't identify any references that were irrelevant or redundant... This longer list is probably due again to the "double explanation" factor that was needed throughout the paper to give sufficient background to both the space sector readers and the science readers.

**Confidential to Editor**<br><br>1. Do you have any potential or perceived competing interests that may influence your review? Please review our Competing Interests policy and declare any potential interests that you feel the Editor should be aware of when considering your review. If you have no competing interests, please write "I have no competing interests."	I have no competing interests
2. Did you receive any assistance in preparing this review (e.g. from a post-doc or graduate student)? If yes, please include their name below.	
3. If accepted, do you think this submission should be highlighted on the PLOS ONE website? PLOS ONE does not evaluate manuscripts based on perceived significance or readership. We aim to provide tools for readers to filter and evaluate our publications. (optional)	Yes, on the homepage
**Do you want to get recognition for this review on a Web of Science researcher profile?**<br><br>If you opt in, your Web of Science profile will automatically be updated to show a verified record of this review in full compliance with the journal's review policy. If you don't have a Web of Science profile, you will be prompted to create a free account.	Yes

Attachments:

Action	Description	File Name	Size	Last Modified
Download<br>Download Sanitized Copy	PONE-D-23-12961_R1_reviewer	PONE-D-23-12961_R1_reviewer.pdf	3.6 MB	Jan 16 2024 02:25PM
Download<br>Download Sanitized Copy	Reviewer: - Comments to the Author: - The article is interesting	Reviewer.pdf	256 KB	Jan 16 2024 02:28PM

More Reviewer Details

Cancel Save and Close
