# Supplementary material for: Intercropping on Mars: A promising system to optimise fresh food production in future martian colonies
Source: PLoS One. 2024 May 1;19(5):e0302149. doi: 10.1371/journal.pone.0302149 (PMC11062560; doi:10.1371/journal.pone.0302149)
Supplement: Attachment — Submitted filename: Response to Reviewers_R_Goncalves.docx [file pone.0302149.s004.docx]

Dear Dr. Harris,

Thank you for your kind feedback on our submitted manuscript. Below, we have indicated in blue the actions taken to address each of your suggested revisions.

Line numbers mentioned here refer to the line numbers on the document with tracked changes on (“Revised Manuscript with Track Changes”).

We hope to have properly addressed your concerns.

Kind regards,

Rebeca Goncalves

Overall, the authors present a novel study with an interesting premise, good study design, and overall suitable metrics to measure their results. The growth of plants in-situ in regolith is a complex and relatively new topic and with limited studies on this subject, these studies will lay groundwork for later experiments. The use of agricultural metrics with intercropping in this paper bring a fresh approach to regolith studies and the use of a plant-rhizobium symbiosis builds on existing literature. The question and build up is mostly there to give context for intercropping in the introduction and the sources cited are relevant articles. However, one concern I have with this sort of research is that it does not address some of the crucial issues that growing things in-situ on Mars would encounter. That does not make this any less useful, but it maybe good to mention some of the other things that would need to be dealt with just to highlight where the focus of this research is. The data provided adequately supports the authors claims, but the discussion could use some elaboration to better understand how it fits the context of the

field as a whole. The paper is an important contribution to a growing field and with a few minor revisions I would recommend this paper for publication. I have below outlined proposed revisions and additions to the article.

Major revisions:

None

Minor revisions:

Introduction

Generally speaking, it may be good to address some of the current literature on problems with simulant experiments that have been brought up. Issues like presence of perchlorates and other obstacles that maybe an issue should be briefly mentioned. On this note, addressing the soil properties of regolith a bit more in depth would help readers to understand why it is important to reestablish

agricultural knowledge in this medium. Challenges with Martian regolith, including presence of perchlorates, were added in lines 179-215.

| Line | Revisions recommended |
| --- | --- |
| 160-163 | A justification for why Rhizobium Leguminosarum (RL) was used maybe helpful. This study does not address the specifics of the symbiosis nor study it in detail. There is nothing wrong with this as the focus of the study was elsewhere, but I would alter  the language and explain the choice of using RL as a symbiote over something like Sinorhizobium meliloti which has been tested in multiple studies. Also, the species  “meliloti” is spelled incorrectly in the manuscript. |

The justification was moved to the methods section (lines 367-371). Indeed it seemed more relevant to omit the mention of the other two species and highlight that RL was chosen due to its nodulation success that we saw in a previous in-house experiment using the same MMS-1 regolith.

Methods

| Line | Revisions recommended |
| --- | --- |
| 257 | Hoagland solution misspelled as Hoogland solution |

Fixed, it’s now on line 410.

One thing to acknowledge in the discussion is the use of Hoagland solution and addition of soil to the regolith and sand. Native Martian regolith (and most simulants) have no nitrogen in them.

Adding fertilizer like this inherently alters the fertility of the regolith and thus the focal study system. This maybe needed to support the targets of your study, but it needs to be acknowledged. The bypassing of some of the regolith challenges, including the addition of fertilizer and presence of perchlorates in the regolith, is now acknowledged at the end of the discussion, in lines 806-819.

| Line | Revisions recommended |
| --- | --- |
| 524-543 | Finding some more sources about bacteria or bacterial symbiosis in regolith to help support this section would be excellent. It has a good start and I would say it is almost there but I think there are a few key aspects missing such as pH and presence  of toxins in regolith. |

Other studies involving rhizobia-legume interactions in mars regolith have been mentioned, expanding a little the discussion on reasons why nodulation may have been impaired, which includes the high pH factor of the regolith (lines 713-719).

Conclusions

| Line | Revisions recommended |
| --- | --- |
| 596-598 | I think this line needs some qualifying elements to it, as it is it overextends a bit.  Under what conditions did this experiment occur? This is not regolith as such, there are issues to overcome. Something along the lines of “with the addition of X  nutrients”. |

The conclusion was rewritten and includes the suggestions mentioned above. Lines 827-849.

Appendix

| Line | Revisions recommended |
| --- | --- |
| 883 | Table unreadable in PDF format |

Fixed font size.

Additional revisions:

The abstract was slightly reworded to make some aspects of it clearer.

Reference style has been changed to Vancouver according to journal guidelines. You’ll see the revisions throughout the text. Also, additional references have been included in the reference list.

Tables font have been formatted to match font of text.

Text was standardised to UK English spelling.
